# Truncated variants of MAGEL2 are involved in the etiologies of the Schaaf-Yang and Prader-Willi syndromes

**DOI:** 10.1016/j.ajhg.2025.09.005

**Published:** 2025-09-12

**Authors:** David Heimdörfer, Alexander Vorleuter, Alexander Eschlböck, Angeliki Spathopoulou, Marta Suarez-Cubero, Hesso Farhan, Veronika Reiterer, Melanie Spanjaard, Christian P. Schaaf, Lukas A. Huber, Leopold Kremser, Bettina Sarg, Frank Edenhofer, Stephan Geley, Mariana E.G. de Araujo, Alexander Huettenhofer

## Main text

(The American Journal of Human Genetics *111*, 1383–1404; July 11, 2024)

In the originally published Figure 5D, we reported "RPL15" in the list of proteins. This is a typo and has been corrected to be "MRPL15." Additionally, the original legend for Figure 5C read "...and those identified in 2 of 3 replicates of the BioID or GFP-CoIP ..." This has been corrected to be "...and those identified in at least 2 of 3 replicates of the BioID or GFP-CoIP ..."

**Figure 5:**
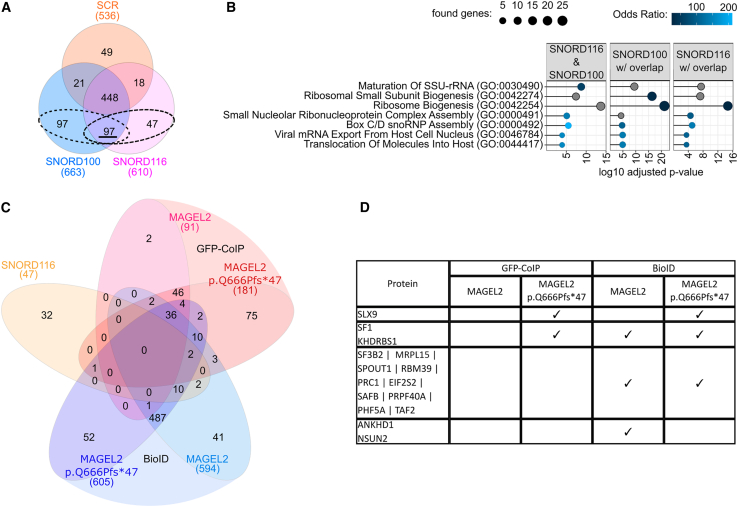
*SNORD116* does not directly interact with WT MAGEL2 or p.Gln666Profs^∗^47, corrected

