# Supplementary material for: Truncated variants of MAGEL2 are involved in the etiologies of the Schaaf-Yang and Prader-Willi syndromes
Source: Am J Hum Genet. 2024 Jun 21;111(7):1383–404. doi: 10.1016/j.ajhg.2024.05.023 (PMC11267527; doi:10.1016/j.ajhg.2024.05.023)
Supplement: Documrnt S1. Figures S1–S6 and Tables S1–S9 [file mmc1.pdf]

The American Journal of Human Genetics, Volume *111*

Supplemental information

Truncated variants of MAGEL2 are involved in the etiologies of the Schaaf-Yang and Prader-Willi syndromes

David Heimdörfer, Alexander Vorleuter, Alexander Eschlböck, Angeliki Spathopoulou, Marta Suarez-Cubero, Hesso Farhan, Veronika Reiterer, Melanie Spanjaard, Christian P. Schaaf, Lukas A. Huber, Leopold Kremser, Bettina Sarg, Frank Edenhofer, Stephan Geley, Mariana E.G. de Araujo, and Alexander Huettenhofer

Supplemental Material

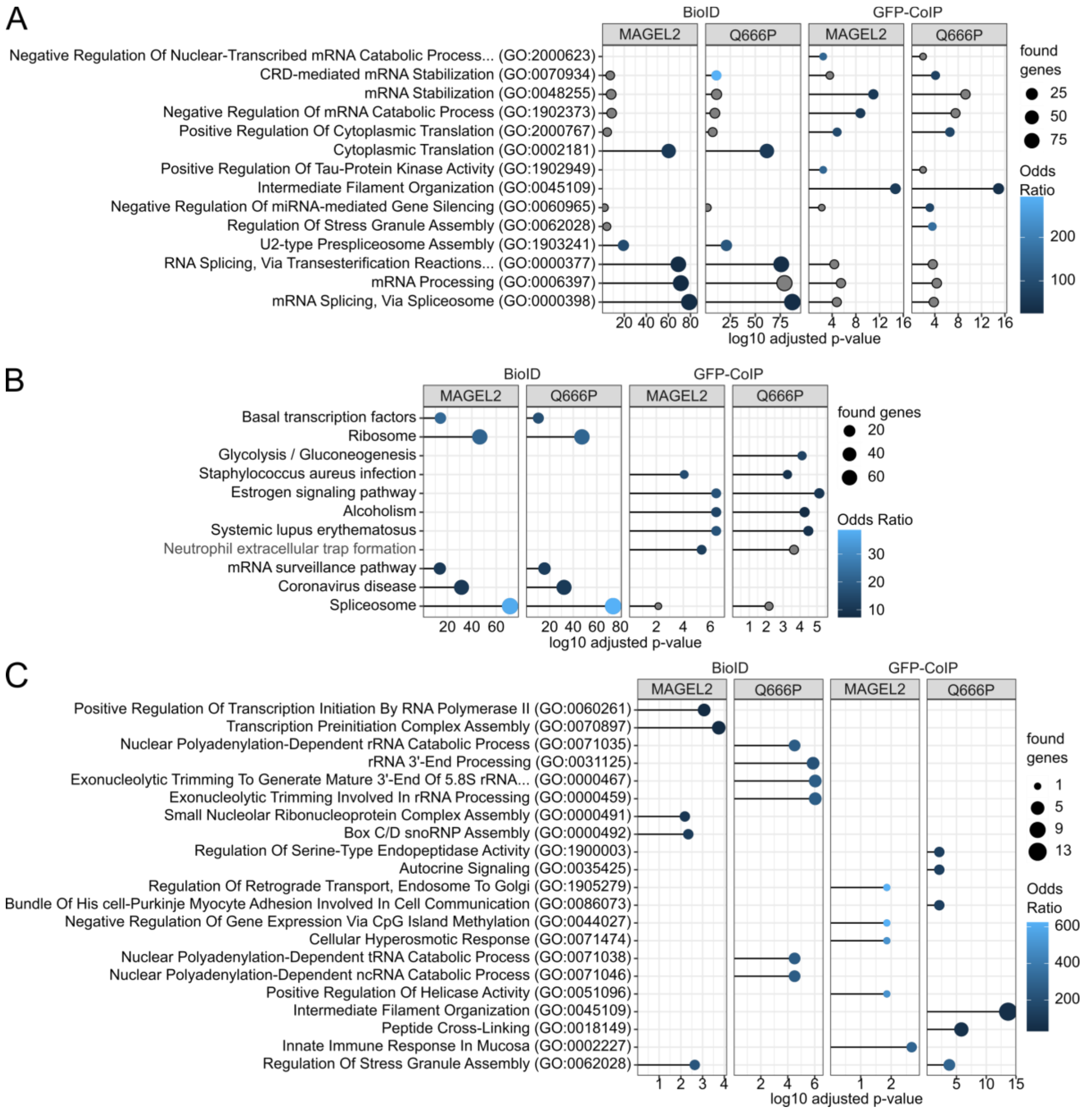

Figure S1: GO enrichment analysis reveals putative interactors of MAGEL2 and MAGEL2 p.(Gln666Profs*47) (referred to as Q666P in the figure) to be involved in transcription, mRNA stabilization, snoRNA metabolism, and metabolism. A and B: GO enrichment analysis for proteins identified in 2 out of 3 replicates showing Biological Processes (A) or KEGG pathways (B). C: GO enrichment analysis of proteins, which were 2-fold enriched in either BioID or GFP-CoIP showing Biological Processes. A-C: GO terms with an adjusted p-value <0.05 were ordered by Combined Score [1]. Top 5 hits are indicated in shades of blue. Light grey circles indicate that the respective GO term is among top hits in one of the other conditions.

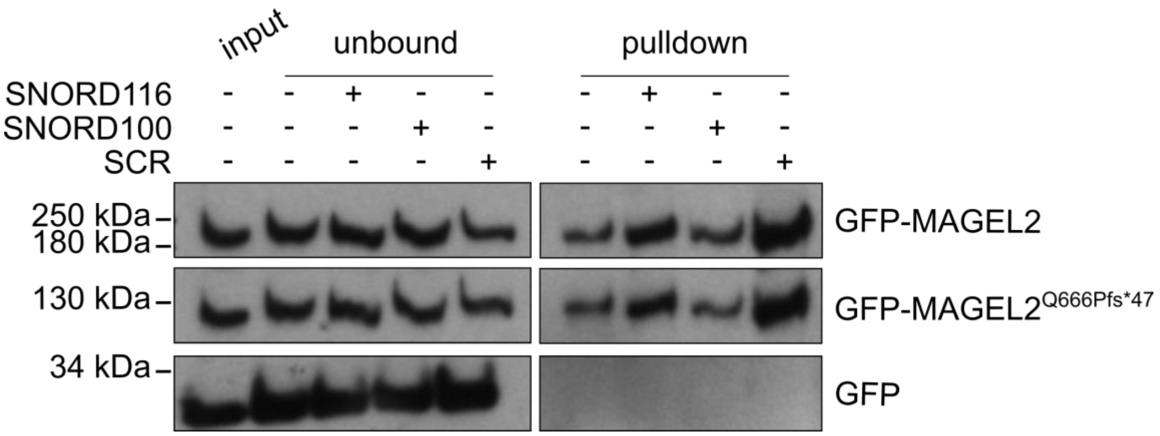

Figure S2: Western blot analysis following RNA-CoIP of HEK293T IP lysates with transiently overexpressed GFP, GFP-MAGEL2 and GFP- MAGEL2 p.(Gln666Profs*47).

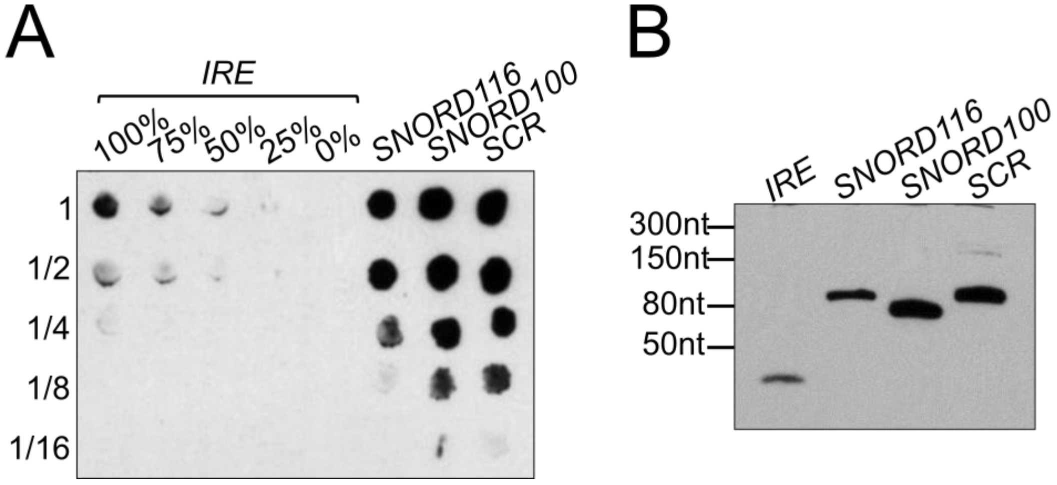

Figure S3: Effieciency of biotinylation of *SNORD116*, *SNORD100* and the *SCR* control. A and B: 3'-biotinylation of RNAs was analyzed by dot blot analysis employing a dilution series (A) and on an 8% polyacrylamide gel (7 M urea) (B). As control served a biotinylated IRE RNA from the company (Thermo Fisher Scientific).

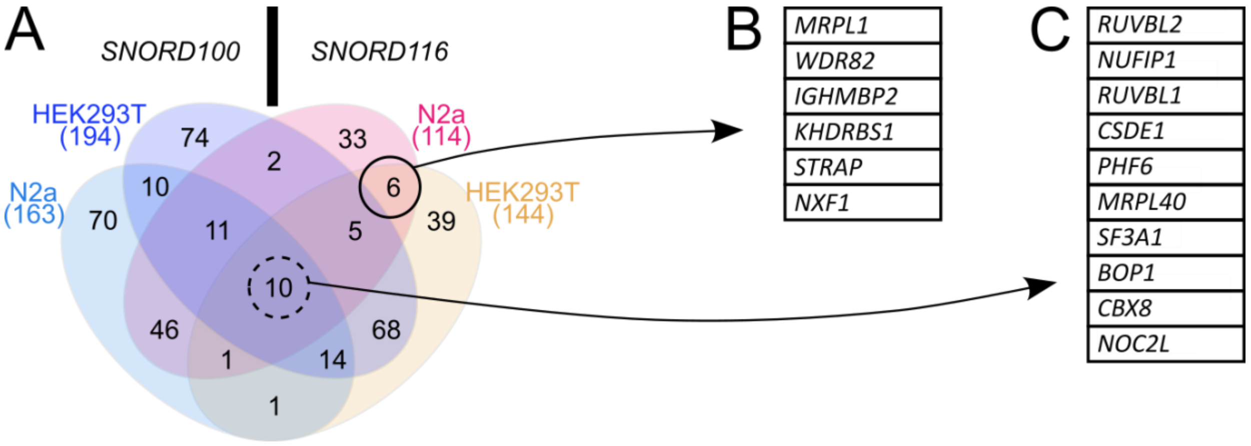

Figure S4: A: Venn diagram of interactomes identified by RNA-CoIP with 3'-biotinylated RNAs in HEK293T and N2A IP lysates. Only proteins identified in at least 2 of 3 replicates were analyzed. Putative unspecific interactors were removed employing a scrambled RNA control (see Fig 4B) for per cell type. B: Specific proteins (gene names depicted) bound to *SNORD116* in HEK293T and N2A. C: Mutual proteins (gene names depicted) of *SNORD116* and *SNORD100* in both examined cell types.

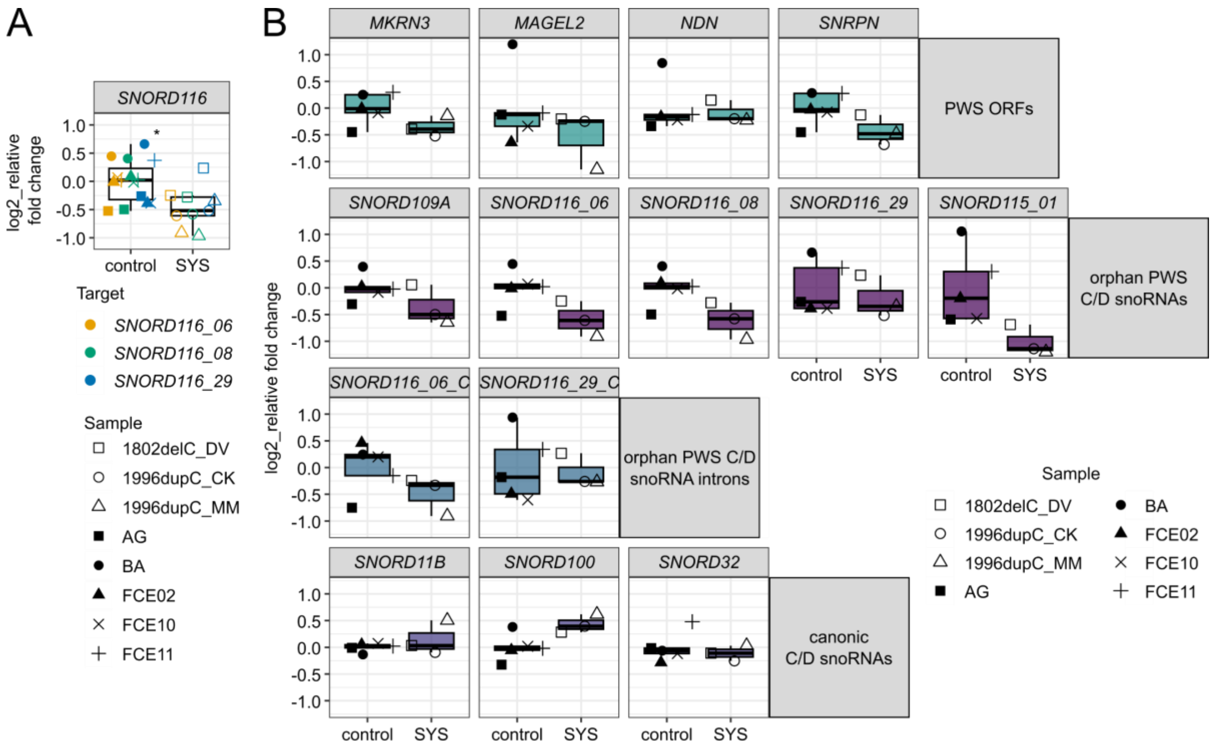

Figure S5: Expression analysis of genes from the PWS region by qPCR analysis. A: Pooled RT-qPCRs for investigated *SNORD116* copies 06, 08, and 29 in smNPCs, rstatix[2] was used for the calculation of Wilcoxon tests B: .RT-qPCR of individual transcripts in smNPCs, which were used for analysis in ***Error! Reference source not found.***A.

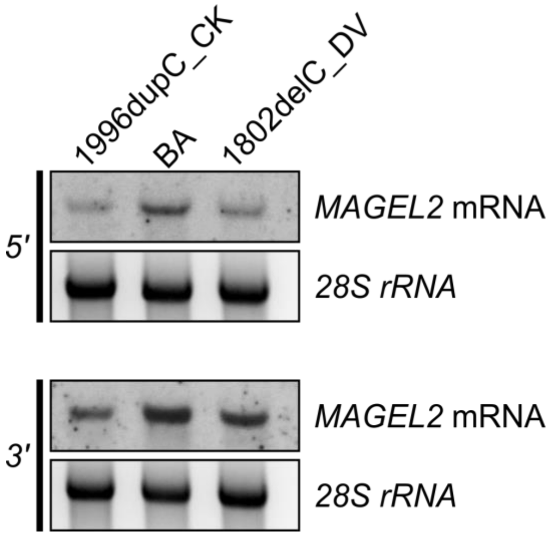

Figure S6: Northern blot analysis of wt and mutant MAGEL2 mRNA transcripts from healthy and SYS smNPCs. Probes for expression analysis of wt (BA) or mutant (1996dupC_CKMAGEL2, 1802delC_DV) mRNAs are indicated as 5' or 3', respectively; as a loading control expression of 28S rRNA is shown.

antibody	company	product-no.	clonality [clone]	host	dilution
**anti-EEA1**	Abcam	ab109110	mono [EPR4245]	rabbit	750 (HEK 293T) 500 (N2a)
**anti-RAB11A**	Cell signaling	#2413	poly	rabbit	50
**anti-FMRP**	Cell Signaling	#4317	poly	rabbit	50
**anti-SMN**	Proteintech	#11708	poly	rabbit	400
**anti-RAB11A**	Thermo Fischer	#715300	poly	rabbit	50
**CoraLite488-conjugated anti-Rabbit**	Proteintech	SA00013-2	poly	goat	250

Table S1: Antibodies used in IF-analyses. All antibodies employed in this study are depicted above, as well as the host they were derived from and the dilution used in the study.

Target	Sequence [5' to 3']	forward [5' to 3']	reverse [5' to 3']
SNORD116_3/9	**GGATCCTAATACGACTCACTATAGGATCGATGATGAGTCCCCCATAAAAACATTCCTTGGAAAAGCTGAACAAAATGAGTGAGAACTCATACCGTCGTTCTCATCGGAACTGAGGTCC**	GGATCCTAATACGACTCAC	GGACCTCAGTTCCGATGA
SNORD100	**GGATCCTAATACGACTCACTATAGCTGTACATGATGACAACTGGCTCCCTCTACTGAACTGCCATGAGGAAACTGCCATGTCACCCTTCTGATTACAGC**	GGATCCTAATACGACTCAC	GCTGTAATCAGAAGGGTGA
SCR	**GCTAATACGACTCACTATAGAAGATCAGTTGGGTGCACGAGTGGGTTACATCGAACTGGATCTCAACAGCGGTAAGATCCTTGAGAGTTTTCGCCCCGAAGAACGTTTTCCAAT**	GCTAATACGACTCACTATAGAAGATCAGTTGGGTGCAC	ATTGGAAAACGTTCTTCGGG

Table S2: Sequences of snoRNAs and SCR control RNA sequences (bold). Sequences were employed for the generation of T7 transcripts and their respective primers are indicated. The T7 promoter sequence is underlined.

antibody	company	product-no.	clonality [clone]	host	dilution
**anti-FMRP**	Abcam	ab264380	poly	rabbit	5,000
**anti-SMN**	Abcam	ab108531	mono [EPR4429]	rabbit	1,000
**anti-NUFIP1**	Cell signaling	#37783	poly	rabbit	1,000
**anti-USP7**	Abcam	ab264422	mono [BLR072G]	rabbit	1,000
**anti-GFP**	Roche	11814460001	mono [7.1 and 13.1]	mouse	1,000
**anti-mouse**	Dako	P0447	poly	goat	3,000
**anti-rabbit**	Thermo Fischer	31463	poly	goat	3,000 - 10,000

Table S3: Antibodies used in western blot analyses. Indicated are all antibodies as well as their clonality, the host they were derived from and the dilution which was employed in experiments.

name	*Sequence [5' to 3']*	*forward [5' to 3']*	*reverse [5' to 3']*
M2_5' (356 nt)	CTGGGTCTCTCTGAAAGCCCAGGG AGCTCTCTGCCCGTAGTTGTGTCTG AGGTCGCAAGTGTCTCTCCGGGAT CCAGTGCCACCCAGGATAATTCCAA GGTGGAGGCACAGCCCTTGTCTCC CTTGGATGAGAGGGCAAATGCGTT GGTGCAGTTCCTCTTAGTCAAGGAC CAAGCCAAGGTGCCTGTCCAGCGC TCGGAGATGGTGAAAGTCATCCTCC GAGAGTATAAAGATGAGTGCTTAGA TATCATCAACCGTGCCAACAATAAG CTGGAGTGTGCCTTTGGTTATCAAT TGAAAGAAATTGATACCAAAAACCA CGCCTATATTATCATCAACAAGCTG GGCTACCATACAGGGAATTTGGTGG CATCCTATTTAGACAGGCCC	GTACACCGATGG CCCAG	AACTTGAGACTGG ATTTGCAG
M2_3' (390 nt)	CTGGGTCTCTCTGAAAGCCCAGGG AGCTCTCTGCCCGTAGTTGTGTCTG AGGTCGCAAGTGTCTCTCCGGGAT CCAGTGCCACCCAGGATAATTCCAA GGTGGAGGCACAGCCCTTGTCTCC CTTGGATGAGAGGGCAAATGCGTT GGTGCAGTTCCTCTTAGTCAAGGAC CAAGCCAAGGTGCCTGTCCAGCGC TCGGAGATGGTGAAAGTCATCCTCC GAGAGTATAAAGATGAGTGCTTAGA TATCATCAACCGTGCCAACAATAAG CTGGAGTGTGCCTTTGGTTATCAAT TGAAAGAAATTGATACCAAAAACCA CGCCTATATTATCATCAACAAGCTG GGCTACCATACAGGGAATTTGGTGG CATCCTATTTAGACAGGCCC	CTGGGTCTCTCTG AAAGCCCAG	GGGCCTGTCTAA ATAGGATGCC

Table S4: DIG probe sequences and oligonucleotides employed for probe generation. Forward and reverse primers for PCR amplification are indicated.

**Target**	**Forward [5‘ to 3‘]**	**Reverse [5‘ to 3‘]**
**GAPDH**	CCATGGGGAAGGTGAAGGTC	AGTTAAAAGCAGCCCTGGTGA
**EID2**	GGCATCGCTCTGTCCAGTTA	GCTTGGACATCTCAGACCGT
**TBP**	GCAAGGGTTTCTGGTTTGCC	CAAGCCCTGAGCGTAAGGTG
**HPRT**	AGGCGAACCTCTCGGCTTTC	CTGGTTCATCATCACTAATCACGAC
**TUBB**	CAACCAGATCGGGGCCAAGTT	GAGGCACGTACTTGTGAGAAGA
**MAGEL2**	CAAGTTTGGCCTTCTGATGGTG	CGGACATCCAACCCTAACTTGA
**MKRN3**	CATTGAGTTTGTTCCAGGGCAG	TCTGCTTCCTCTCAGTCTCTGA
**SNORD115_1**	GAGAACCTTATATTATCCTGAAGAGA GGTG	GGCCTCAGCGTAATCCTATTGA
**SNORD116_29**	GGACCTCAGCTCACAGAAGTG	GGATCGATGATGACTTAAAAAAATGG AAAC
**SNORD116_08**	GGATCGATGATGAGTCCTCCAA	GGACCTCAGTTCCGATGAGAAC
**SNORD116_06**	GGATCGATGATGAGTCCTCCAA	GGACCTCAGTTCCGATGAGAAT

**SNORD116_06_C**	GTGGAAGTTGTCCCTCACTAGTA	CAAAATGAGTGAAAACTCATACCGTC
**SNORD116_29_C**	CAAACTGAACGCCCTCCAC	AAATGAGTGACCAAGACACTTCTG
**SNRPN**	ACTGTTGGCAAGAGTAGCAAGA	GGCCATCTTGCAGGATACATCT
**NDN**	TACTCCACGAGGGTGTTTTCTG	AGTTTGGAAGAAGTCACCAGCA
**SNORD109A**	GGATCGATGATGAGAATAATTGTC	GGACCTCAGATTGACATCTG
**SNORD11B**	CTGATGGCAATGATGATTTTTACAC	GATGGCATCAGATGAGGTAGT
**SNORD32**	CAGTGATGAGCAACATTCACCA	GAGCGGTGCATGGGGTTGAT
**SNORD100**	GCTGTACATGATGACAACTGGC	GTGACATGGCAGTTTCCTCATG

Table S5: Primers used in qPCR analysis. For quantification of gene expression by qPCR forward and reverse primers for respective genes are indicated.

**MAGEL2**			**MAGEL2 p.(Gln666Profs*47)**		
Position	Sequence	Score	Position	Sequence	Score
4-33	LSKNLGDSSPPAEAPKPPV YSRPTVLMRAP	4.3	4-33	LSKNLGDSSPPAEAPKPPV YSRPTVLMRAP	4.3
277-307	PMAKPPGPGVLMIHPPGAR APMTQPPASGAP	2.3	277-307	PMAKPPGPGVLMIHPPGAR APMTQPPASGAP	2.3
292-322	PGARAPMTQPPASGAPMA QPAAPPAQPMAPP	2.3	292-322	PGARAPMTQPPASGAPMA QPAAPPAQPMAPP	2.3
494-527	RQAPPPIRPAPQVLATQPPL WQALPPPPPLRQAP	3.1	494-527	RQAPPPIRPAPQVLATQPPL WQALPPPPPLRQAP	3.1
587-617	WQAPKGQPPVPHEIPTSM EFQEVQQTQALAW	2.5	587-617	WQAPKGQPPVPHEIPTSM EFQEVQQTQALAW	2.5
617-648	WQAQKAPTHIWQPLPAQE AQRQAPPLVQLEQP	2.8	617-648	WQAQKAPTHIWQPLPAQE AQRQAPPLVQLEQP	2.8
654-684	PSQKAVQIQLPPQQAQAS GPQAEVPTLPLQP	2.5	654-683	PSQKAVQIQLPPPAGPGIG SASGGAHTAAP	2.1
709-739	GSAKSLMTPSGECRASSID RRGSSKERRTSS	3.1			
728-760	RRGSSKERRTSSKERRAPS KDRMIFAATFCAPK	3.0			
735-764	RRTSSKERRAPSKDRMIFA ATFCAPKAVSA	2.5			
735-766	RRTSSKERRAPSKDRMIFA ATFCAPKAVSAAR	3.0			
735-770	RRTSSKERRAPSKDRMIFA ATFCAPKAVSAARAHLP	2.0			
742-774	RRAPSKDRMIFAATFCAPK	2.4			

	AVSAARAHLPAAWK				
802-832	NAFKGPSAASETPKSLPYA LQDPFACVEALP	2.7			
880-914	RRSGKATRKKKHLEAQEDS RGHTLAFHDWQGPRPW	2.9			
880-915	RRSGKATRKKKHLEAQEDS RGHTLAFHDWQGPRPWE	2.3			
881-915	RSGKATRKKKHLEAQEDS RGHTLAFHDWQGPRPWE	2.9			
887-916	RKKKHLEAQEDSRGHTLAF HDWQGPRPWEN	2.5			
952-979	SRILSGWEGPSASWALSA WEGPSTSRAL	2.4			
1161-1192	FVRQKYLEYRRIPYTEPAE YEFLWGPRAFLET	2.0			
1163-1197	RQKYLEYRRIPYTEPAEYE FLWGPRAFLETSKMLV	2.4			
883-893	GKATRKKKHLE	8.5			

Tab S6: Nuclear localization signal (NLS) motif prediction performed with the cNLS mapper online tool [3]. NLS are listed for MAGEL2 protein and the truncated MAGEL2 p.(Gln666Profs*47). Shown are amino acid position, NLS candidate sequence and cut-off score.

MAGEL2			MAGEL2 p.(Gln666Profs*47)		
Position	Sequence	Score	Position	Sequence	Score
65-79	WEAPQGQLPAPVVPM	0.024	65-79	WEAPQGQLPAPVVPM	0.022
235-249	PGVLMAQPLTPGVLM	0.011	235-249	PGVLMAQPLTPGVLM	0.124
236-250	GVLMAQPLTPGVLMV	0.068	236-250	GVLMAQPLTPGVLMV	0.255
592-606	GQPPVPHEIPTSMEF	0.042	592-606	GQPPVPHEIPTSMEF	0.031
668-682	AQASGPQAEVPTLPL	0.022			
694-708	QAQPGPPVAAANFPL	0.046			
842-856	MNASKASQAVPTFLM	0.017			
985-999	PGSSLPVVVSEVASV	0.074			
1016-1030	PLSPLDERANALVQF	0.109			
1018-1032	SPLDERANALVQFLL	0.144			
1019-1033	PLDERANALVQFLLV	0.186			
1103-1117	SYLDRPKFGLLMVVL	0.262			

1105-1119	LDRPKFGLLMVVLSL	0.204			
1106-1120	DRPKFGLLMVVLSLI	0.115			
1107-1121	RPKFGLLMVVLSLIF	0.473			
1108-1122	PKFGLLMVVLSLIFM	0.201			
1123-1137	KGNCVREDLIFNFLF	0.051			
1127-1141	VREDLIFNFLFKLGL	0.332			
1129-1143	EDLIFNFLFKLGLDV	0.238			
1146-1160	TNGLFGNTKKLITEV	0.007			
1148-1162	GLFGNTKKLITEVFV	0.005			
1183-1197	LWGPRAFLETSKMLV	0.015			
1184-1198	WGPRAFLETSKMLVL	0.186			
1186-1200	PRAFLETSKMLVLRF	0.084			

Table S7: Nuclear export signal (NES) motif prediction performed with the LocNES online sever tool [4]. NES are listed for MAGEL2 and the truncated MAGEL2 p.(Gln666Profs*47). Shown are amino acid position, NES candidate sequence and probability score.

Term	Genes
**Positive Regulation Of DNA-templated Transcription (GO:0045893)**	**HMGA1, SFPQ, TAF15, ILF3, HMGN1, YBX1**
**Regulation Of DNA-templated Transcription (GO:0006355)**	**FUS, ILF3, YBX1, HMGA1, SFPQ, TAF15**
**Regulation Of Nucleic Acid-Templated Transcription (GO:1903506)**	**FUS, HMGA1, SFPQ, YBX1**
**Regulation Of Transcription By RNA Polymerase II (GO:0006357)**	**FUS, YBX1, HMGA1, SFPQ**
Negative Regulation Of DNA-templated Transcription (GO:0045892)	HMGA1, SFPQ, ILF3
Negative Regulation Of Nucleic Acid-Templated Transcription (GO:1903507)	ILF3, HMGA1, SFPQ
Positive Regulation Of Nucleic Acid-Templated Transcription (GO:1903508)	ILF3, HMGA1, TAF15
Positive Regulation Of Transcription By RNA Polymerase II (GO:0045944)	HMGA1, SFPQ, YBX1
DNA-templated Transcription Initiation (GO:0006352)	ELOB
Negative Regulation Of Transcription By RNA Polymerase II (GO:0000122)	SFPQ
Positive Regulation Of DNA-templated Transcription, Elongation (GO:0032786)	HMGN1
Positive Regulation Of Transcription Regulatory Region DNA Binding (GO:2000679)	H1-0
Regulation Of DNA-templated Transcription Elongation (GO:0032784)	HMGN1
Regulation Of Transcription Regulatory Region DNA Binding (GO:2000677)	H1-0

Transcription By RNA Polymerase II (GO:0006366)	ELOB
Transcription Initiation At RNA Polymerase II Promoter (GO:0006367)	ELOB

Table S8: Mutual protein interaction partners of MAGEL2 and MAGEL2 p.(Gln666Profs*47) with implication in transcription.

Summarized term	GO terms included
Transcription	Transcription By RNA Polymerase II (GO:0006366)
	Transcription Initiation At RNA Polymerase II Promoter (GO:0006367)
	Transcription Preinitiation Complex Assembly (GO:0070897)
snoRNAs	Box C/D snoRNP Assembly (GO:0000492)
	Polyadenylation-Dependent snoRNA 3'-End Processing (GO:0071051)
	snoRNA Localization (GO:0048254)
mRNA stabilization	3'-UTR-mediated mRNA Stabilization (GO:0070935)
	CRD-mediated mRNA Stabilization (GO:0070934)
	mRNA Stabilization (GO:0048255)
	Regulation Of 3'-UTR-mediated mRNA Stabilization (GO:1905868)
Pyruvate metabolism and Gluconeogenesis	Gluconeogenesis (GO:0006094)
	Negative Regulation Of Gluconeogenesis (GO:0045721)
	Pyruvate Metabolic Process (GO:0006090)
	Positive Regulation Of Gluconeogenesis (GO:0045722)
	Regulation Of Gluconeogenesis (GO:0006111)

Table S9: GO terms significantly regulated in the two different approaches (BioID and GFP-CoIP).

Supplemental References

1. Chen, E.Y., Tan, C.M., Kou, Y., Duan, Q., Wang, Z., Meirelles, G.V., Clark, N.R., and Ma'ayan, A. (2013). Enrichr: interactive and collaborative HTML5 gene list enrichment analysis tool. BMC Bioinformatics *14*, 128. https://doi.org/10.1186/1471-2105-14-128.

2. Kassambara, A. (2023). rstatix: Pipe-Friendly Framework for Basic Statistical Tests.

3. Kosugi, S., Hasebe, M., Tomita, M., and Yanagawa, H. (2009). Systematic identification of cell cycle-dependent yeast nucleocytoplasmic shuttling proteins by prediction of composite motifs. Proc. Natl. Acad. Sci. U. S. A. *106*, 10171–10176. https://doi.org/10.1073/pnas.0900604106.

4. Xu, D., Marquis, K., Pei, J., Fu, S.-C., Cağatay, T., Grishin, N.V., and Chook, Y.M. (2015). LocNES: a computational tool for locating classical NESs in CRM1 cargo proteins. Bioinforma. Oxf. Engl. *31*, 1357–1365. https://doi.org/10.1093/bioinformatics/btu826.
