# Supplementary material for: Truncated variants of MAGEL2 are involved in the etiologies of the Schaaf-Yang and Prader-Willi syndromes
Source: Am J Hum Genet. 2024 Jun 21;111(7):1383–404. doi: 10.1016/j.ajhg.2024.05.023 (PMC11267527; doi:10.1016/j.ajhg.2024.05.023)
Supplement: Document S2. Article plus supplemental information [file mmc2.pdf]

Truncated variants of MAGEL2 are involved in the etiologies of the Schaaf-Yang and Prader-Willi syndromes

Graphical abstract

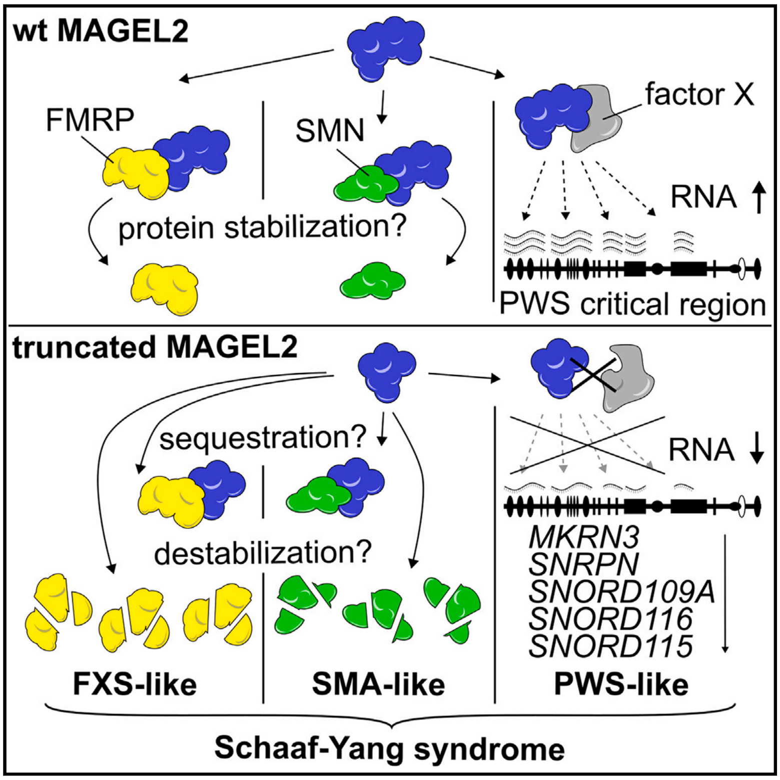

Authors

David Heimdörfer,
Alexander Vorleuter,
Alexander Eschlböck, ...,
Stephan Geley,
Mariana E.G. de Araujo,
Alexander Huettenhofer

Correspondence

david.heimdoerfer@i-med.ac.at (D.H.),
alexander.huettenhofer@i-med.ac.at
(A.H.)

Mutations within *MAGEL2* from chromosomal region 15q11–q13 cause Schaaf-Yang syndrome, which is phenotypically related to Prader-Willi syndrome, caused by deletion of the *SNORD116* cluster within the same locus. We correlate mutations within *MAGEL2* to spinal muscular atrophy and autism and also demonstrate its influence on the abundance of *SNORD116*.

Heimdörfer et al., 2024, The American Journal of Human Genetics *111*, 1383–1404

July 11, 2024 © 2024 The Author(s).

https://doi.org/10.1016/j.ajhg.2024.05.023

Truncated variants of MAGEL2 are involved in the etiologies of the Schaaf-Yang and Prader-Willi syndromes

David Heimdörfer,[1,7,*] Alexander Vorleuter,[1,7] Alexander Eschlböck,[4] Angeliki Spathopoulou,[4] Marta Suarez-Cubero,[4] Hesso Farhan,[2] Veronika Reiterer,[2] Melanie Spanjaard,[3] Christian P. Schaaf,[3] Lukas A. Huber,[5] Leopold Kremser,[6] Bettina Sarg,[6] Frank Edenhofer,[4] Stephan Geley,[2] Mariana E.G. de Araujo,[5] and Alexander Huettenhofer[1,*]

Summary

The neurodevelopmental disorders Prader-Willi syndrome (PWS) and Schaaf-Yang syndrome (SYS) both arise from genomic alterations within human chromosome 15q11–q13. A deletion of the *SNORD116* cluster, encoding small nucleolar RNAs, or frameshift mutations within *MAGEL2* result in closely related phenotypes in individuals with PWS or SYS, respectively. By investigation of their subcellular localization, we observed that in contrast to a predominant cytoplasmic localization of wild-type (WT) MAGEL2, a truncated MAGEL2 mutant was evenly distributed between the cytoplasm and the nucleus. To elucidate regulatory pathways that may underlie both diseases, we identified protein interaction partners for WT or mutant MAGEL2, in particular the survival motor neuron protein (SMN), involved in spinal muscular atrophy, and the fragile-X-messenger ribonucleoprotein (FMRP), involved in autism spectrum disorders. The interactome of the non-coding RNA *SNORD116* was also investigated by RNA-CoIP. We show that WT and truncated MAGEL2 were both involved in RNA metabolism, while regulation of transcription was mainly observed for WT MAGEL2. Hence, we investigated the influence of *MAGEL2* mutations on the expression of genes from the PWS locus, including the SNORD116 cluster. Thereby, we provide evidence for MAGEL2 mutants decreasing the expression of *SNORD116*, *SNORD115*, and *SNORD109A*, as well as protein-coding genes *MKRN3* and *SNRPN*, thus bridging the gap between PWS and SYS.

Introduction

Prader-Willi syndrome (PWS [MIM: 176270]) and Schaaf-Yang syndrome (SYS [MIM: 615547]) are two related rare genetic neuro-developmental disorders emerging from mutations in the maternally imprinted locus on paternal chromosome 15q11–q13.[1] This region harbors paternally expressed genes exclusively, i.e., *MKRN3* (MIM: 603856), *MAGEL2* (MIM: 605283), *NDN* (MIM: 602117), *PWRN1* (MIM: 611215), *NPAP1* (MIM: 610922), *SNRPN* (MIM: 182279), *SNHG14* ([MIM: 616259]; its introns contain *SNORD107*, *SNORD64*, *SNORD108*, *SNORD109A*, *SNORD 109B*, *SNORD115*, and *SNORD116*), and *IPW* (MIM: 601491).[2]

PWS appears in 1 in 16,000 to 1 in 21,000 live births[3] and is caused by either (1) a 5–6 Mb *de novo* deletion within the paternal Prader-Willi critical region (65%–75% of the cases), (2) maternal uniparental disomy 15 (20%–30% of the cases), or (3) imprinting center defects (1%–3% of the cases).[1,4] Clinical features of PWS are developmental delay, hypotonia, endocrine dysfunction, intellectual disability, and in particular hyperphagia resulting in severe obesity.[5]

Recent studies showed that micro-deletions of 71–200 kb in size, encompassing the entire *SNORD116* cluster only, are sufficient to cause PWS.[6–10] *SNORD116* belongs to the class of box C/D small nucleolar RNAs (snoRNAs). Together with all other snoRNAs from the PWS region, *SNORD116* was initially discovered in our lab by an RNA-seq approach for non-coding RNAs for which we coined the term RNomics.[11,12] Canonical box C/D snoRNAs guide 2′-*O*-methylation of precursor ribosomal and small nuclear RNAs within the nucleolus by base-pairing.[13,14] The majority of snoRNAs are embedded in intronic regions of RNA Polymerase II transcribed protein-coding[15,16] or long non-coding RNA (lncRNA) genes.[17,18] Subsequent to intron splicing and debranching, processing and maturation of pre-snoRNAs involves 5′- and 3′-end exonucleolytic trimming[19,20] and binding to snoRNP proteins.[17,21,22]

Both *SNORD116* and *SNORD115* are uniquely organized within the PWS critical region in tandem repetitive multi-copy gene arrays of 30[23] and 48[6] copies, respectively, share high sequence homology within their cluster[11] and are post-transcriptionally processed from their long non-coding host *IC-SNURF-SNRPN* transcript.[24] In contrast to canonical snoRNAs, we were able to demonstrate that both *SNORD116* and *SNORD115* snoRNAs lack obvious sequence complementarity within their antisense element toward canonical target pre-rRNAs or snRNAs.[11]

[1]Institute of Genomics and RNomics, Biocenter Innsbruck, Medical University of Innsbruck, Innrain 80/82, 6020 Innsbruck, Austria; [2]Institute of Pathophysiology, Biocenter, Medical University of Innsbruck, Innrain 80/82, 6020 Innsbruck, Austria; [3]Institute of Human Genetics, Heidelberg University, Im Neuenheimer Feld 366, 69120 Heidelberg, Germany; [4]Institute for Molecular Biology, Genomics, Stem Cell Biology & Regenerative Medicine Group, University of Innsbruck and CMBI, Technikerstr. 25, 6020 Innsbruck, Austria; [5]Institute of Cell Biology, Biocenter, Medical University of Innsbruck, Innrain 80/82, Innsbruck 6020, Austria; [6]Institute of Medical Biochemistry, Protein Core Facility, Biocenter, Medical University of Innsbruck, Innrain 80/82, 6020 Innsbruck, Austria
[7]These authors contributed equally
*Correspondence: david.heimdoerfer@i-med.ac.at (D.H.), alexander.huettenhofer@i-med.ac.at (A.H.)
https://doi.org/10.1016/j.ajhg.2024.05.023.
© 2024 The Author(s). This is an open access article under the CC BY license (http://creativecommons.org/licenses/by/4.0/).

Molecular functions of orphan mammalian *SNORD116* and its host gene still remain to be fully elucidated. Human PWS phenotypes were partially recapitulated in mice carrying a deletion of the paternal *Snord116* cluster.[25,26] Although these mice did not develop obesity, they showed retarded growth, failure to thrive, and reduced feeding.[25,26] In addition, hyperphagia, increased anxiety, and reduced motor learning were also observed in mice with PWS.[25] The flanking non-coding exons of the *SNORD116* copies were shown to be processed into a functional lncRNA, designated as lncRNA *HG116* which was suggested to regulate transcription of several target genes in a diurnal manner.[27] Furthermore, rhythmic methylation of CpG islands was disrupted in a *Snord116* deletion mouse model.[28]

SYS is a very rare disease with a prevalence of <1 in 1,000,000[29] and was originally described by Schaaf et al.,[30] when they reported four individuals with nonsense or frameshift indel mutations within MAGEL2 who displayed PWS or PWS-like phenotypes. Individuals with SYS show developmental delay, mild to profound intellectual disability, neonatal hypotonia with respiratory distress and central apnea, joint contractures, and social behavioral characteristics.[1,31–33] Moreover, autism spectrum disorder or autistic features are diagnosed in 75%–80% of affected individuals as well as hyperphagia resulting in subsequent obesity which can develop as SYS-affected individuals get older.[1,31–33]

Up until now, several mutations within the single exon gene *MAGEL2* have been described with a correlation between the severity of phenotypes and the genotype of affected individuals.[31,32,34] Persons carrying a *MAGEL2* c.1996delC (p.Gln666Serfs*36) mutation passed away during gestation or hours after birth.[31,32,35] The c.1996dupC is not only the most prevalent but also the *MAGEL2* mutation resulting in the most severe phenotype.[32]

MAGEL2 (melanoma antigen L2) belongs to the MAGE protein family of RING E3 ubiquitin ligase regulators.[36–38] It was initially shown to specifically bind to the RING-type E3 ubiquitin transferase TRIM27 and the ubiquitin-specific protease USP7 to form a MAGE-RING E3 ligase complex, termed MUST, which is recruited by the retromer to early and recycling endosomes to facilitate F-actin polymerization.[37–39] However, additional protein interaction partners have also been identified, implicating MAGEL2 in other biological functions.[40]

Magel2-null mice were shown to recapitulate some PWS phenotypes, such as growth retardation, excessive weight gain,[41] and differences in circadian regulation.[42] In addition, rats with paternal *Magel2* truncation exhibit changes in body composition, cardiac structure and function, breathing, as well as sociability and anxiety-like behavior, also observed in SYS-affected individuals.[43]

Because of their similar phenotypes as well as their common genomic location, in this study we investigated a potential functional cross-talk between both PWS and SYS. To this end, we first cloned WT *MAGEL2* as well as mutated *MAGEL2* c.1996dupC (p.Gln666Profs*47) (GenBank: NM_019066.5) as mCherry- or GFP-fusion proteins, resulting in the WT and truncated p.Gln666Profs*47 variant and addressed their subcellular localization. In addition, we attempted to identify novel protein interaction partners by either BioID (miniTurbo-fusion proteins) or co-immunoprecipitation (CoIP; GFP-fusion proteins). To identify potential common interaction partners between MAGEL2 and *SNORD116*, we further analyzed the interactome of *SNORD116* by RNA-CoIP.

Our data showed an involvement of MAGEL2 in biological processes such as RNA stabilization, chromosome condensation, and transcription. Based on these findings, we investigated the involvement of WT and truncated MAGEL2 on the expression of selected genes from the PWS locus in small molecule neural precursor cells (smNPCs) derived from persons suffering from SYS and compared them to healthy control subjects. Interestingly, the expression of most genes within the PWS locus—including *SNORD116*—was found to be down-regulated in SYS cell lines carrying the *MAGEL2* c.1996dupC (see above) or the *MAGEL2* c.1802delC (p.Pro601Glnfs*101) mutation (GenBank: NM_019066). Taken together, our results thus suggest a functional link between SYS and PWS.

Material and methods

Cloning of reporter plasmids

Plasmids were designed with the Benchling software (https://benchling.com). Plasmid constructs were cloned by Gibson Assembly or mutagenesis PCR (NEB) and Oligonucleotides for Gibson Assembly and mutagenesis PCR were designed with the online NEBuilder Assembly Tool 2.0 and NEBaseChanger Tool (NEB), respectively. Gene fragments and oligonucleotides were purchased from IDT.

Human *SNORD116*-3 (GenBank: LN847959) and human *SNORD100* (GenBank: LN847822) were designed with a T7 promoter sequence in the 5′ end and flanked by BamHI and EcoRI restriction sites. snoRNA genes were amplified by PCR from gene fragments (IDT), digested by the respective enzymes, and ligated into a PUC19 vector. Single-exon *MAGEL2* (ENSG00000254585) was amplified by PCR from human genomic DNA. Due to the high GC-content within the coding sequence of *MAGEL2* (>70% in the proline-rich region), the PCR was prone to premature termination. To circumvent this issue, the sequence was divided into three equal parts for amplification. Amplicons were analyzed by gel electrophoresis, and PCR products with the correct length were gel excised and purified using the Monarch DNA Gel Extraction Kit (NEB).

The *MAGEL2* sequence was cloned into the Low0 vector backbone employing Gibson Assembly (NEB). A plasmid harboring the *MAGEL2* c.1996dupC mutation, resulting in the truncated MAGEL2 p.Gln666Profs*47 variant, was generated via Gibson Assembly. Furthermore, various plasmids for the expression of WT MAGEL2 and p.Gln666Profs*47 fusion proteins were generated. To this purpose, either mCherry, GFPspark, or miniTurbo[44] were cloned N-terminally to the respective *MAGEL2* variant by Gibson Assemby, thereby preserving the endogenous 3′ UTRs of *MAGEL2* and *MAGEL2* c.1996dupC transcripts. Plasmids were transformed

into homemade chemically competent NEB 5-alpha *E. coli*, and plasmid DNA of selected clones was purified by the Monarch Plasmid Miniprep Kit (NEB) and sequenced by Eurofins Mix2Seq (Eurofins). Positive clones were re-cultured and DNA was extracted employing the QIAGEN Plasmid Midi Kit (QIAGEN).

Cell culture

HEK293T (ATCC CRL-3216) or Neuro-2a (ATCC CCL-131) (N2a) cells were grown in pyruvate-depleted, 4.5 g/L glucose- and L-glutamine-containing DMEM medium (Gibco) supplemented with 100 U/mL penicillin/streptomycin and 10% heat-inactivated FCS (Gibco).

HeLa cells (HeLa ACC57, DSMZ) were cultured in 4.5 g/L glucose and L-glutamine containing DMEM medium (Sigma-Aldrich) supplemented with 100 U/mL penicillin/streptomycin and 10% FCS (Thermo Fisher Scientific).

Induced pluripotent stem cell (iPSC) lines were cultured on hESC-qualified Matrigel-coated plates (Corning) with StemMACS iPS-Brew (Miltenyi), passaged with Accutase (Sigma-Aldrich), and supplemented with 10 μM Y-27632 (Miltenyi) for 24 h. The cells were cryopreserved in knock-out serum replacement (Thermo Fisher Scientific) supplemented with 10% DMSO (Roth) and stored at −80°C until further usage.

smNPCs were cultured with N2B27 medium (1:1 mixture of DMEM/F12 and Neurobasal [Gibco] supplemented with 0.5% *N*-2 [Gibco], 1% B-27 minus Vitamin A [Gibco], 1× GlutaMAX [Gibco], 3 μM CHIR 99021 [Axon Medchem], 0.5 μM Purmorphamine [Miltenyi], and 150 μM ascorbic acid [Sigma]). The cells were passaged with Accutase (Sigma-Aldrich) and cryopreserved in knock-out serum replacement (Thermo Fisher Scientific) supplemented with 10% DMSO (Roth) and stored at −80°C until further usage. All cell lines were maintained at 37°C, 5% CO_2, and saturated humidity culturing conditions.

Healthy and Schaaf-Yang syndrome proband cell lines and iPSC reprogramming

Human control fibroblast lines AG, FCE02, FCE10, and FCE11 were obtained from the Coriell Institute for Medical Research. All the procedures were in accordance with the Office for Human Research Protections, Department of Health and Human Services ("DHHS") regulations for the protection of human subjects (45 CFR Part 46). Fibroblasts were cultured with fibroblast medium (DMEM [Thermo Fisher Scientific], 15% FBS [Thermo Fisher Scientific], 1× non-essential amino acids [Sigma]). The medium was changed every second day and the cells were passaged using Trypsin-EDTA solution (Sigma). The cells were cryopreserved in 90% FBS supplemented with 10% DMSO (Roth) and stored at −80°C until further usage. Fibroblasts were reprogrammed into iPSCs employing the CytoTune iPS 2.0 Sendai Reprogramming Kit (Thermo Fischer Scientific) according to the manufacturer's guidelines with some modifications. In brief, 40,000 fibroblasts were plated per 24 well a day prior to the viral transduction. The cells were transduced with the recommended viral multiplicity of infection (MOI). After 24 h the viral medium was removed and the medium was renewed every second day. On day 7 the cells were replated on hESC-qualified Matrigel-coated 6-well plates (Corning). From day 8 onwards, the fibroblast medium was replaced with StemMACS iPS-Brew (Miltenyi) and was renewed daily. Around day 12, iPSC putative colonies started to appear and once they reached the appropriate size they were manually isolated and transferred on hESC-qualified Matrigel-coated 48 wells (Corning). The colonies were further expanded, characterized, and eventually cryopreserved in knock-out serum replacement (Thermo Fisher Scientific) supplemented with 10% DMSO (Roth) and stored at −80°C until further usage. The pluripotency of the cells was confirmed by immunofluorescence staining for pluripotency-associated markers and differentiation of the cells into the three germinal layers (STEMdiff Trilineage Differentiation Kit, Stemcell Technologies). Moreover, the absence of Sendai-associated transgene expression was confirmed via RT-qPCR.

Human SYS fibroblast lines were obtained from skin biopsies of SYS-affected individuals carrying the *MAGEL2* c.1802delC or *MAGEL2* c.1996dupC mutations and healthy control individuals (BA cell line). From respective individuals under human research protocol H-34578 of the Institutional Review Board of Baylor College of Medicine, Houston, USA, proper informed consent was obtained. Fibroblasts were reprogrammed into iPSCs by the Baylor College of Medicine Human Stem Cell Core (HSCC) using the CytoTune-iPS 2.0 Sendai Reprogramming Kit (Life Technologies), following the manufacturer's protocol. Following transduction, cells were grown in high-glucose DMEM supplemented with 10% fetal bovine serum and 1× MEM non-essential amino acids (Life Technologies) for 5 days, in TeSR-E7 (StemCell) for 8 days, and in TeSR-E8 (StemCell) until day 21 when pluripotent colonies were manually picked. Immunofluorescence and RT-qPCR for pluripotency markers were performed on all iPSC lines as described before.[45] Each iPSC line was shown to have a normal karyotype.

smNPC derivation

smNPC lines were derived from iPSCs following the protocol of Reinhardt et al.[46] Briefly, upon 50%–70% confluency, iPSCs were mechanically dissociated with 0.5 mM EDTA (Gibco) and transferred to low-adherence plates in order to form embryoid bodies. After two days, the media was changed to N2B27 medium (1:1 mixture of DMEM/F12 and Neurobasal [Gibco] supplemented with 0.5% *N*-2 [Gibco], 1% B-27 minus Vitamin A [Gibco], 1× GlutaMAX with 10 μM SB-431542 [MedChem Express], 1 μM Dorsomorphin [Miltenyi], 3 μM CHIR 99021 [Axon Medchem], and 0.5 μM Purmorphamine [Miltenyi]). On day 4, media was changed to N2B27 medium supplemented with 3 μM CHIR 99021, 0.5 μM Purmorphamine, and 150 μM Ascorbic acid (Sigma-Aldrich). On day 6, embryoid bodies were triturated with a pipette and seeded as a monolayer on Growth Factor Reduced Matrigel-coated wells (Corning). Subsequently, the cells were further expanded and homogenized by high passaging ratios. After 5–6 passages, the cell morphology appeared homogeneous and the cells were further maintained with N2B27 medium on Growth Factor Reduced Matrigel-coated wells. Successful induction of the neural precursor fate was validated by immunofluorescence stainings for NPC markers.

Leptomycin B treatment

50,000 HeLa cells were seeded in a 12-well plate containing glass cover slips. 24 h after seeding, cells were transiently transfected with a plasmid encoding either Low0_GFPspark_*MAGEL2* or Low0_GFPspark_*MAGEL2* c.1996dupC using PEI (PolySciences). 48 h after transfection cells were left untreated or treated with 20 nM Leptomycin B (Cell Signaling) for 20 h. Cells were fixed with 4% paraformaldehyde, permeabilized with 0.1% Triton X-100, and stained for DAPI. Coverslips were mounted on microscopy slides using polyvinylalcohol mounting medium (Sigma-Aldrich). Images were acquired with a 40× objective (Plan Apo λ 40×) using a Spinning Disc Eclipse Ti-2 microscope.

Fiji was used for image analysis to determine the nuclear cytosolic ratio of Low0_GFPspark_MAGEL2 or Low0_GFPspark_MAGEL2 p.Gln666Profs*47. DAPI staining was used to distinguish the nucleus from the cytosol. A region of interest was selected in the nucleus and a region of similar size was selected in the cytosol. The mean fluorescent intensity of these regions was measured and the ratio of nuclear to cytosolic signal was calculated. Data were statistically analyzed employing the Wilcoxon test.

Immunofluorescence staining

15 mm VWR No.1 coverslips (VWR) were coated in 12-well plates (Greiner Bio-One) with Poly-D-Lysine (Sigma-Aldrich) for 5 min, washed twice with DPBS (Gibco), and dried for at least 2 h. Plates were optionally stored at 4°C up to two weeks. Wells were washed again with DPBS and 10^5 HEK293T or N2A cells were seeded 24 h before transfection. Per expression vector, 750 ng of DNA were transfected with 1.5 μL Metafectene Pro (Biontex).

24 h after transfection, the cultivation medium of the transfected HEK293T or N2A cells was aspirated and the coverslips were washed twice with DPBS. All subsequent steps were conducted in the dark. Cells were incubated in 4% paraformaldehyde in 1× cytoskeletal buffer (CB; 10 mM Pipes [pH 6.8], 150 mM NaCl, 5 mM EGTA [pH 9], 5 mM glucose, 5 mM $MgCl_2$) for 8 min at RT and washed twice in 1× CB. At this point, cells were either stored in 1× CB at 4°C or immediately subjected to the immunofluorescence steps described below.

For EEA1 and RAB11A staining, the CB was discarded and cells were incubated in IF blocking buffer (2% gelatin [w/v], 50 mM NH_4Cl, 1× CB, 0.025% Saponin [w/v]; supplemented with BSA) for at least 45 min at RT, followed by incubation with the primary antibody in IF blocking buffer for 1 to 2 h at RT. For stainings in HEK293T cells, rabbit α-EEA1 (Abcam, ab109110) or rabbit α-RAB11A (Cell Signaling, #2413S) were used in a 1:750 or 1:50 dilution, respectively. Endogenous proteins in N2a cells were stained with α-EEA1 (Abcam, ab109110) and rabbit α-RAB11A (Thermo Fischer, #715300) were used in a 1:500, 1:50 dilution, respectively. Coverslips were washed 6 times with IF washing buffer (50 mM NH_4CL, 1× CB) for 5 min at RT and incubated in CoraLite488 goat α rabbit IgG (Proteintech, SA00013-2) diluted in 1:250 in IF blocking buffer for 40 to 60 min at RT. After 6 washing steps in IF washing buffer for 5 min at RT, cells were counterstained with DAPI (f.c. 1 μg/mL) in IF washing buffer for 1 min and subsequently washed 3 times in IF washing buffer for 5 min. Finally, coverslips were mounted in 20 μL Mowiol on microscope slides and stored light protected at 4°C until microscopy.

For staining of endogenous FMRP and SMN, the fixed N2a cells (see above) were incubated in 0.2% Triton X-100 in DPBS and subsequently washed three times in DPBS for 5 min. After blocking of unspecific binding in 5% BSA (Roth) in DPBS-T (0.1% Tween 20 [Roth] in DPBS) (BB) for at least 1 h at RT, the primary antibody was incubated in BB for 1–1.5 h at RT. α-SMN (Proteintech, #11708) or α-FMRP (Cell Signaling, #4317) antibodies were applied in a 1:400 or 1:50 dilution, respectively. After washing the cells thrice in DPBS-T for 5 min, the secondary CoraLite488 goat α rabbit IgG antibody (Proteintech, SA00013-2) was incubated in a 1:250 dilution in BB for 40–60 min at RT. After three consecutive washing steps in DPBS-T for 5 min at RT, DAPI (f.c. 1 μg/mL) was applied in DPBS-T for 1 min at RT. The coverslips were washed three times in DPBS-T and mounted as described above. Antibodies used are listed in Table S1.

Confocal microscopy and image processing

Confocal fluorescence microscopy was performed on the Zeiss LSM980 Airyscan 2 microscope using the ZEN 3.5 software (Carl Zeiss Microscopy GmbH) in the Airyscan super resolution mode. Images were acquired as Z-stacks with an LD LCI Plan-Apochromat 63×/1.2 objective with Immersol G glycerine immersion oil (Zeiss) and using an LSM T-PMT. Excitation wavelength was preset by the software and is stated for DAPI = 405 nm, mCherry = 561 nm, and CoraLite488 = 488 nm. Deconvolution of images was performed employing the Classic Maximum Likelihood Estimation (CMLE) algorithm in the Huygens Software (Scientific Volume Imaging) and for further image processing and figure compilation the software Fiji[47] was used.

In vivo proximity biotinylation

2.5 × 10^6 HEK293T cells were seeded in 100 mm dishes. After 24 h, cells were transiently transfected with 5 μg of plasmids harboring *MAGEL2* or *MAGEL2* c.1996dupC with an N-terminally fused HA-miniTurbo and 20 μL of Metafectene Pro. 48 h post transfection, the culture medium was discarded and cells were synchronized in starvation medium (DMEM, supplemented with 1% Pen/Strep) supplemented with 200 μM (f.c.) biotin for 4 h. HEK293T cells were carefully washed once with 1× DPBS and scraped in fresh DPBS. After centrifugation at 500 × *g* for 5 min, cells were resuspended in 500 μL RIPA lysis buffer (50 mM Tris-HCl [pH 7.4]; 1% Igepal CA-630; 0.25% SDS, 150 mM NaCl; 1 mM EDTA [pH 8.0]; 1× cOmplete [Roche]) according to Hutter et al.[48] and incubated on ice for 10 min. Subsequently, the cell debris was pelleted for 10 min at 14,000 × *g* and the protein concentration was determined by Bradford assay employing RotiQuant (Roth). Affinity capture of biotinylated proteins was performed as described before.[48] Briefly, 600 μg of total protein lysate was diluted in 500 μL RIPA lysis buffer supplemented with 60 μL of Streptavidin Magnetic Beads (NEB) and incubated overnight at 4°C under constant rotation. For mass spectrometric analysis, beads with biotinylated proteins were washed twice with RIPA lysis buffer.

Protein co-immunoprecipitation

2.5 × 10^6 HEK293T cells were seeded in 100 mm dishes and grown for 24 h. Subsequently, cells were transfected with 5 μg of Low0_GFPspark_*MAGEL2* or Low0_GFPspark_*MAGEL2* c.1996dupC plasmids and 20 μL of Metafectene Pro (Biontex) and incubated for 48 h. HEK293T cells were washed with ice-cold DPBS (Gibco), scraped in 500 μL ice-cold homemade Pierce IP Lysis Buffer (25 mM Tris-HCl [pH 7.4]; 150 mM NaCl; 1 mM EDTA; 1% Igepal CA-630; and 5% glycerol; supplemented with 1× [f.c.] cOmplete protease inhibitor [Roche]) and incubated on ice for at least 15 min. Lysates were sonicated twice with 5 pulses at 0.5 cycles and 30 mA (Hielscher) on ice and 5% were retained for later SDS-PAGE analysis. 20 μL Affi-Prep Protein A slurry (BioRad) were washed three times in 200 μL Pierce IP Lysis Buffer at 1,000 × *g* for 1 min at 4°C (centrifugation setting for all steps) and then incubated with 500 μL cell lysate on a rotation wheel for 90 min at 4°C. 20 μL of 25% covalently coupled anti-GFP (Roche) Affi-Prep Protein A slurry (BioRad) were washed thrice in 200 μL Pierce IP lysis buffer (supplemented with cOmplete protease inhibitor [Roche]). 25 μL of precleared cell lysate were saved for later analysis and the remains was added to the washed anti-GFP-Protein A beads and incubated overnight at 4°C under rotation. Samples were centrifuged and supernatant was stored. Beads were washed thrice in 1 mL IP Lysis Buffer (with cOmplete

protease inhibitor [Roche]), twice in 1 mL 0.5× DPBS, twice in 1 mL 0.1× DPBS, and 3 times in 1 mL MilliQ-water. Co-immunoprecipitated proteins were eluted from beads twice with 2% formic acid for 15 min. At the second elution step, samples were vortexed after 10 min. After adjusting the volume of protein mixtures to 200 μL with 2% formic acid, 20 μL were saved for later SDS-PAGE and samples were analyzed by mass spectrometry.

When samples were prepared for Western blot analysis, cell seeding, transfection, cell lysis, and pre-clearing was conducted as described above or 6.25×10^6 HEK293T cells were seeded in 150 mm dishes. For that purpose, all following reagents and buffers were upscaled 2.5-fold accordingly—the following protocol refers to 2.5×10^6 starting cell number for 100 mm dishes.

Pre-cleared IP cell lysates were incubated with 2.5 ng/μL (f.c.) mouse anti-GFP antibody (Roche, #11814460001) at 4°C overnight under rotation. 20 μL Affi-Prep Protein A slurry (BioRad) were washed three times with 200 μL IP lysis buffer, supplemented with cOmplete protease inhibitor (Roche). Subsequently, they were incubated with the cell lysate/GFP-antibody mixture at 4°C for 90 min. After three wash steps in 1 mL IP lysis buffer, supplemented with cOmplete protease inhibitor (Roche), bound proteins were eluted from the beads in at least 1.5 bead volumes 1.5× Lämmli buffer at 95°C for 5 min with periodical vortexing.

RNA co-immunoprecipitation

snoRNA genes and a scrambled RNA (amplified from the AmpR gene) sequence harboring a 5′-T7 promoter sequence were amplified by PCR on respective plasmids using Phusion High-Fidelity DNA Polymerase (Thermo Fisher Scientific) (for primers and RNA sequences refer to Table S2). Amplicons were checked on a 1% Agarose gel and purified by Monarch PCR & DNA Cleanup Kit (5 μg) (NEB). Transcripts were generated employing the HiScribe T7 High Yield RNA Synthesis Kit (NEB). After template DNA was digested with 4 U RQ1 DNase (Promega), T7 transcripts were PAGE purified from a 8% denaturing polyacrylamide gel by UV shadowing and passively eluted in gel elution buffer (300 mM NaCl; 0.2% sodium dodecyl sulfate; 60 mM NaOAc [pH 5.2]) under agitation overnight at 4°C as described by Hoernes et al.[49] Samples were incubated at 60°C for 1 h and stored on ice until RNA was purified. This particular step was performed using the Monarch RNA Cleanup Kit (50 μg) (NEB). Transcripts were biotinylated by use of Pierce RNA 3′ End Biotinylation Kit (Thermo Fisher Scientific) according to the manufacturer's protocol. Thereby, a single biotinylated cytidine (bis)phosphate is ligated to the 3′-end of the respective transcripts in presence of DMSO at 16°C overnight. Biotinylated RNAs were purified utilizing Monarch RNA Cleanup Kit (10 μg) (NEB). Ligation efficiency was determined by northern blot analysis and dot blotting, followed by detection employing the Pierce Chemiluminescent Nucleic Acid Detection Module Kit (Thermo Fisher Scientific).

For LC-MS/MS analysis, HEK293T cells were washed with ice-cold DPBS (Gibco), scraped in ice-cold homemade Pierce IP Lysis Buffer (25 mM Tris-HCl [pH 7.4]; 150 mM NaCl; 1 mM EDTA; 1% Igepal CA-630; and 5% glycerol; supplemented with 1× [f.c.] cOmplete protease inhibitor [Roche]) and incubated for 5 min on ice. Cell debris was pelleted by centrifugation at 13,000 × *g* for 10 min at 4°C. Protein concentration was determined by Bradford assay with ROTIQuant (Roth).

40 pmol of biotinylated RNA were renatured at 65°C for 10 min, at RT for 20 min, and stored on ice until they were immobilized on 20 μL Dynabeads M-280 Streptavidin (Thermo Fisher Scientific) in 30 min incubation time, otherwise following the manufacturer's protocol. Immobilized RNA-bead mixture was washed two times in 1× Protein-RNA binding buffer (20 mM Tris [pH 7.5]; 150 mM NaCl; 2 mM $MgCl_2$; 0.1% Tween-20); pull-down was performed by a modified protocol from Pierce Magnetic RNA-Protein Pull-Down Kit (Thermo Fisher Scentific). Binding was performed in a 200 μL RNA-protein binding reaction containing 1 μg/μL protein lysate, 1× Protein-RNA binding buffer, 15% glycerol, 0.4 U/μL of RNase inhibitor (Promega), and 1× cOmplete protease inhibitor (Roche) at 4°C for 1 h on a rotation wheel. Beads were washed thrice with 200 μL IP wash buffer (20 mM Tris [pH 7.5], 150 mM NaCl, 0.1% Tween-20) and thrice with 500 μL 50 mM Tris (pH 7.5).

Sample preparation for western blot analysis had modifications, i.e., only 20 pmol of biotinylated RNA were used and all other reagents were downscaled accordingly. Transfection of HEK293T with Low0_GFPspark, Low0_GFPspark_*MAGEL2*, or Low0_GFPspark_*MAGEL2* c.1996dupC plasmids as well as cell lysate preparation were conducted as described above. After Bradford analysis, all three lysates were mixed in equal parts according to their protein concentration and 100 μg of this lysate mix were used for immunoprecipitation. After the third wash step with IP wash buffer, proteins were eluted in 40 μL 1× Lämmli buffer at 95°C for 5 min.

LC-MS/MS analysis

Proteins from BioID and RNA-CoIP were reduced with dithiothreitol, alkylated with iodoacetamide, and digested with trypsin (Promega) according to Branon et al.[44] Then samples were purified using Pierce C18 Tips, 100 μL (Thermo Fisher Scientific) according to the manufacturer's instructions prior to nano LC-MS analysis.

Samples from Protein-CoIP were dried in a speedvac and dissolved in 33 μL 100 mM NH_4HCO_3 buffer (pH 8.0). Subsequently 33 μL of 10 mM DTT in 100 mM NH_4HCO_3 were added and incubated at 56°C for 30 min. Thereafter 1.0 μg trypsin (Promega) was added to the sample and incubated at 37°C for 6 h. Finally, 33 μL of 55 mM IAA dissolved in 100 mM NH_4HCO_3 were added and incubated at room temperature for 20 min in the dark.

Digested samples were analyzed using an UltiMate 3000 nano-HPLC system coupled to a Q Exactive HF mass spectrometer (Thermo Fisher Scientific) equipped with a Nanospray Flex ionization source. The peptides were separated on a homemade fritless fused-silica microcapillary column (75 μm i.d. × 280 μm o.d. × 16 cm length) packed with 2.4 μm reversed-phase C18 material (Reprosil). Solvents for HPLC were 0.1% formic acid (solvent A) and 0.1% formic acid in 85% acetonitrile (solvent B). The gradient profile was as follows: 0–4 min, 4% B; 4–57 min, 4%–35% B; 57–62 min, 35%–100% B; and 62–67 min, 100% B. The flow rate was 250 nL/min.

BioID samples were analyzed with a Q Exactive HF mass spectrometer. The instrument was operating in the data-dependent mode selecting the top 20 most abundant isotope patterns with charge >1 from the survey scan with an isolation window of 1.6 mass-to-charge ratio (m/z). Survey full-scan MS spectra were acquired from 300 to 1,750 m/z at a resolution of 60,000 with a maximum injection time (IT) of 120 ms and automatic gain control (AGC) target 1e6. The selected isotope patterns were fragmented by higher-energy collisional dissociation (HCD) with normalized collision energy of 28 at a resolution of 30,000 with a maximum IT of 120 ms and AGC target 5e5.

Samples from Protein-CoIP and RNA-CoIP were analyzed with an Orbitrap Eclipse mass spectrometer. The instrument

equipped with a field asymmetric ion mobility spectrometer (FAIMS) interface was operating in the data-dependent mode with compensation voltages (CV) of −45 and −65 and a cycle time of 1 s. Survey full-scan MS spectra were acquired from 375 to 1,500 m/z at a resolution of 240,000 with an isolation window of 1.2 mass-to-charge ratio (m/z), a maximum injection time (IT) of 50 ms, and automatic gain control (AGC) target 400,000. The MS2 spectra were measured in the Orbitrap analyzer at a resolution of 15,000 with a maximum IT of 22 ms, and AGC target of 50,000. The selected isotope patterns were fragmented by higher-energy collisional dissociation with normalized collision energy of 28.

Data analysis was performed using Proteome Discoverer 2.5 (Thermo Fisher Scientific) with search engine Sequest. Depending on the input sample type, the raw files were searched against *Homo sapiens* or *Mus musculus* uniprot databases. Precursor and fragment mass tolerance was set to 10 ppm and 0.02 Da, respectively, and up to two missed cleavages were allowed. Carbamidomethylation of cysteine was set as static modification, and oxidation of methionine was set as variable modifications. Peptide identifications were filtered at 1% false discovery rate.

Western blot analysis

For analyses of co-immunoprecipitation experiments, proteins were denatured for 5 min at 95°C and separated on gradient SDS-PAGE gels in tris-glycine running buffer (24.8 mM Tris, 191.8 mM Glycine, 0.5% SDS) in a Novex Invitrogen XCell SureLock Electrophoresis System (Thermo Fisher Scientific).

Separated proteins were transferred onto a 0.45 μm PVDF membrane (Thermo Fisher Scientific) in 1× transfer buffer (24.8 mM Tris, 191.8 mM glycine, 10% ethanol, 0.08% SDS) in a Novex Invitrogen XCell II Blot Module 1 (Thermo Fisher Scientific) wet blotting apparatus at 20 V, 100 mA for 1 to 4 h.

The membrane was blocked in blocking buffer (1× TBS-T: 137 mM NaCl, 27 mM KCl, 25 mM Tris [pH 7.4], 0.1% Tween 20; supplemented with 5% BSA [Roth]) for at least 1 h at RT and incubated with the primary antibody diluted in blocking buffer for 1–2 h at RT or overnight at 4°C under constant rotation. Membranes were washed thrice in TBS-T for 5 min at RT and incubated with the secondary antibody conjugated to horseradish peroxidase in locking solution for 1 h at RT. Membranes were washed again 3 times in TBS-T for 5 min and signals were detected employing Pierce ECL chemiluminescent substrate (Thermo Fisher Scientific) and Kodak BioMax MS film. Antibodies used for western blot analysis are listed in Table S3.

Northern blot analysis

Total smNPC RNA was isolated employing TRI Reagent (Sigma-Aldrich) according to the manufacturer's protocol. For the detection of MAGEL2 and *MAGEL2* c.1996dupC mRNAs, 10 μg of total RNA were loaded onto a 1.2% denaturing agarose gel in 1× RP buffer (40 mM MOPS, 10 mM sodium acetate, 2 mM EDTA [pH 7.0]) containing 2.17% formaldehyde and the gel was run in RP buffer containing 2.17% formaldehyde at 100 V for 50 min. The gel was washed 2 times in 10× SSC (1.5 M NaCl, 150 mM sodium citrate [pH 7.2]) for 15 min. RNA was transferred onto an Amersham Hybond-N^+ nylon membrane (GE Healthcare) employing passive capillary blotting in 10× SSC overnight. Subsequently, the membrane was rinsed with 2× SSC, shortly air dried, and the RNA was cross-linked to the membrane with 254 nm at 0.12 Joule in a UV cross-linker (Stratagene).

Digoxigenin (DIG)-labeled probes for detection were produced by PCR employing a dNTP mix containing a DIG-UTP:dTTP ratio of 1:2 (for primers refer to Table S4). *M2_5′* and *M2_3′* probes (Table S4) anneal within the 5′ and 3′ portion of the *MAGEL2* transcript, respectively. After pre-hybridization in high-SDS solution (7% SDS, 50% deionized formamide, 20% blocking solution, 10% blocking reagent [Roche], 1× maleic acid buffer [100 mM maleic acid, 150 mM NaCl, 100 mM NaOH (pH 7.5)], 1% sodium lauroyl sarcosinate) at 42°C for 4 h, cross-linked membranes were hybridized with 15 mL high-SDS solution containing DIG-labeled probes at 42° overnight. Subsequently, the membranes were washed three times in 1× SSC supplemented with 0.1% SDS at RT for 10 min and two times at 65°C for 15 min. 5 min incubation in DIG wash buffer (99.7% 1× maleic acid buffer, 0.3% Tween 20) were followed by DIG-P2 (90% 1× maleic acid buffer, 10% blocking reagent [Roche]) incubation for 30 min, before alkaline phosphatase conjugated anti-DIG antibody solution (99.99% DIG-P2, 0.01% anti-DIG antibody [Roche]) was applied for 30 min at RT.

Membranes were washed once in DIG wash buffer for 5 min, twice for 10 min at RT, and incubated in DIG-P3 buffer (0.1 M Tris, 0.1 M NaCl, 50 mM $MgCl_2$ [pH 9.5]) for 5 min, before the DIG CSPD substrate solution (99% DIG-P3, 1% CSPD [Roche]) was applied for additional 5 min at RT. Signals were detected applying Kodak BioMax MS films (Kodak) to the membranes at 37°C for approximately 2 h and developed.

For the detection of ncRNAs, 15 μg of total RNA were separated on 8% denaturing polyacrylamide gels (acrylamide:bisacrylamide = 29:1, 7 M urea in 1× TBE) at 120 to 370 V for 2 to 3 h. After ethidium bromide staining for 10 min, the RNA was transferred onto Amersham Hybond-N+ membranes employing the Trans-Blot SD Semi-Dry Transfer Cell (Bio-Rad) at 15 V and 400 mA for 45 min. The RNA was UV-crosslinked at 0.12 kJ in a UV crosslinker (Stratagene) and ncRNAs of interest were detected by 5′-radioactively labeled oligonucleotides. Therefore, oligos (in total 1.2 μM f.c.) were labeled with 1 μCi/μL [γ-32P]-ATP (Hartmann Analytic) by 0.8 U/μL T4 PNK (NEB) for at least 30 min at 37°C and denatured at 96°C for 1 min. After equilibration of cross-linked membranes in hybridization buffer (178 mM Na_2HPO_4, 822 mM NaH_2PO_4, 7% (v/v) SDS [pH 6.2]), the labeling mix was added for overnight incubation at 42°C under constant rotation. Blots were washed in wash buffer I (300 mM NaCl, 34 mM sodium citrate, 0.1% (w/v) SDS [pH 7]) for 10 min and wash buffer II (15 mM NaCl, 1.7 mM sodium citrate, 0.1% (w/v) SDS [pH 7]) for 5 min and rinsed in Aqua dest. Signals were detected employing a phosphorimager and Typhoon FLA 9500 (GE Healthcare).

If necessary, blots were stripped in 0.5% (w/v) SDS at 65°C for 1 h, rinsed with Aqua dest., incubated in Aqua dest. at 65°C for 30 min, and rinsed again with Aqua dest. At least 19 *SNORD116* copies were detected with three probes (5′-GGACCTCAGTTCC GATGA-3′ for copies 1–3, 6, 8, and 9; 5′-GGACCTCAGTTCGAC GAG-3′ for copies 12 and 16–22; copy 10 with 2 mismatches; and 5′-GGACCTCAGCTCACAGAA-3′ for copies 25–27, 29, and 30). 5′-GGGCCTCAGCGTAATCCT-3′ was used to detect several copies of *SNORD115* (1, 4–16, 21, 24–26, 29, 30, 33, 34, 36, and 38–44). 5.8S rRNA (5′-GCAATTCACATTAATTCTCGCAGCTAGC-3′) and U6 snRNA (5′-TATGGAACGCTTCACGAATTTG-3′) were used as controls.

RT-qPCR

167 ng/μL total RNA was reverse transcribed using the SuperScript IV VILO Master Mix with ezDNase Enzyme (Thermo Fisher

Scientific) according to the manufacturer's protocol. 2 μL of 1/100 diluted cDNA was amplified with 3 μL Luna Universal qPCR Master Mix (NEB) and 200 nM forward and reverse primers (Table S5) each in 6 μL reaction volume in 384-well plates using QuantStudio 5 Real-Time PCR System (AppliedBiosystems). After quality control (QC, i.e., melt-curve analysis and outlier removal—samples were analyzed only when at least 2 of 3 technical replicates passed QC), primer efficiencies were calculated from amplification curves using web-based LinRegPCR (https://www.gear-genomics.com/rdml-tools/).[50] Inter-plate variation was corrected employing RDML-Analyze from rdml-tools.[51] RefFinder[52,53] was used to find the most stable housekeeping genes *GAPDH*, *TBP*, and *HPRT*. Percentage changes were calculated by subtracting the median fold changes (as calculated by Ruijter et al.[54] of SYS and control cell lines. For comparison Wilcoxon test was used.

Computational analyses

R and R Studio was used for most computational analyses. For general data processing the packages rio and tidyverse[55] were used. Mass spectrometric-identified protein abundances were normalized to mean total protein abundances per condition, followed by removal of proteins discovered with only one respective peptide or no available abundance. Only proteins with a reviewed status in the uniprot database and present in at least two replicates in at least one condition were included in the analysis. Proteomics analysis was conducted by the DEP package.[56] In brief, abundances of single replicates were normalized using variance stabilizing transformation (VSN). Missing value imputation was performed per sample by mixed imputation. Proteins present in two of three or one of three replicates were defined as missing at random (MAR) or missing not at random (MNAR), respectively. MARs were imputed by maximum likelihood estimation and MNARs were imputed with zero. For differential enrichment analysis, DEP's "test_diff" function was modified, so that FDR was adjusted for the p value instead of t-statistic. Proteins with an FDR < 0.05 were assumed statistically significant.

Before GO enrichment analysis, the datasets from differential enrichment analysis were cleansed of general contaminant proteins by use of the CRAPome database.[57] For proximity-based *in vivo* biotinylation, the CRAPome database was filtered for cell/tissue type "HEK293," subcellular fractionation "total cell lysate" and "total cell lysate and chromatin," epitope tag "BirA*-FLAG," and affinity approach "Streptavidin." For GFP-fusion protein CoIP, we used the filters cell/tissue type "HEK293," subcellular fractionation "total cell lysate and chromatin," and affinity approach "anti-GFP mouse." For each gene, spectral counts (designated as "PSMs"—number of peptide spectrum matches—in Proteome Discoverer 2.5) were averaged across the three replicates and across the retrieved datasets from the CRAPome database. Subsequently, per BioID and GFP-CoIP only proteins with at least twice as many spectral counts than the control dataset were used for further analysis. As for RNA-CoIPs, no public contaminant lists are available, so we included a scrambled RNA control in the experiments. Because it was not possible to quantify bait amount in our experimental design, we conducted GO enrichment analysis with proteins which were found in at least two of three replicates, whereupon interactors of scrambled control were removed from obtained interactor lists of *SNORD100* or *SNORD116* pull-downs employing interactivenn.[58] Gene ontologies were analyzed using the EnrichR R package which connects to the enrichr web-server.[59–61] Upon GO enrichment analysis functional categories were sorted by combined score[60] and top 5 hits per condition were depicted.

Statistics

Plots were generated with ggplot2[62] or ggpubr. R-package rstatix was used for statistical analyses.

NLS and NES prediction tool

The computational prediction of potential nuclear localization signal (NLS) motifs for human MAGEL2 WT and the truncated p.Gln666Profs*47 variant was performed using the cNLS Mapper online tool[63] with a cut-off score of 2.0 as well as the NLStradamus online tool[64] with a prediction cut-off of 0.5 in the 4 state HMM static model. Potential nuclear export signal (NES) motif prediction was performed using the LocNES server.[65]

Results

The truncated p.Gln666Profs*47 variant accumulates predominantly within the nucleus while WT MAGEL2 shuttles between the cytoplasm and the nucleus

Human MAGEL2 is a 1,249 amino acid protein with a MAGE homology domain (MHD) and a USP7 binding site (U7BS) in the C-terminal domain, located at residues 1,027 to 1,195 and 820 to 1,034, respectively.[36,37,40] The N-terminal region (aa 1–819) is an intrinsically disordered domain containing 28% proline, 15% alanine, and 11% glutamine residues.[40]

The *MAGEL2* c.1996dupC frameshift mutation represents the most commonly found pathogenic variant among individuals with SYS and shows the second most severe phenotype, with the only exception of the *MAGEL2* c.1996dupC mutation which has been reported to be associated with pre- or perinatal lethality.[32]

In the present study, we initially investigated the subcellular localization of the predominant, truncated p.Gln666Profs*47 variant and its WT counterpart. Subsequently, we aimed to decipher functional interaction partners of WT and mutant MAGEL2 proteins and to investigate molecular mechanisms which might be connected to the expression and/or function of *SNORD116* snoRNAs as well as protein-coding genes from the PWS locus. To this end, we first generated expression vectors in which mCherry or GFP tags were fused in frame to the N terminus of the WT or the c.1996dupC frameshift mutant *MAGEL2* sequence (Figures 1A and 1B) to maintain transcription of the full-length mRNA. Thereby, the inserted c.1996dupC mutation resulted in the truncated MAGEL2 p.Gln666Profs*47 variant due to a premature stop codon (Figures 1A and 1B).

MAGEL2 has previously been shown to bind USP7 and TRIM27 proteins, thus forming the MUST complex.[36,37,66] The trimer was shown to facilitate F-actin polymerization and retrograde recycling on endosomes by ubiquitination of the WASH complex.[36,37,66] Similar to the actin nucleation promoting factor WASH containing complex SHRC

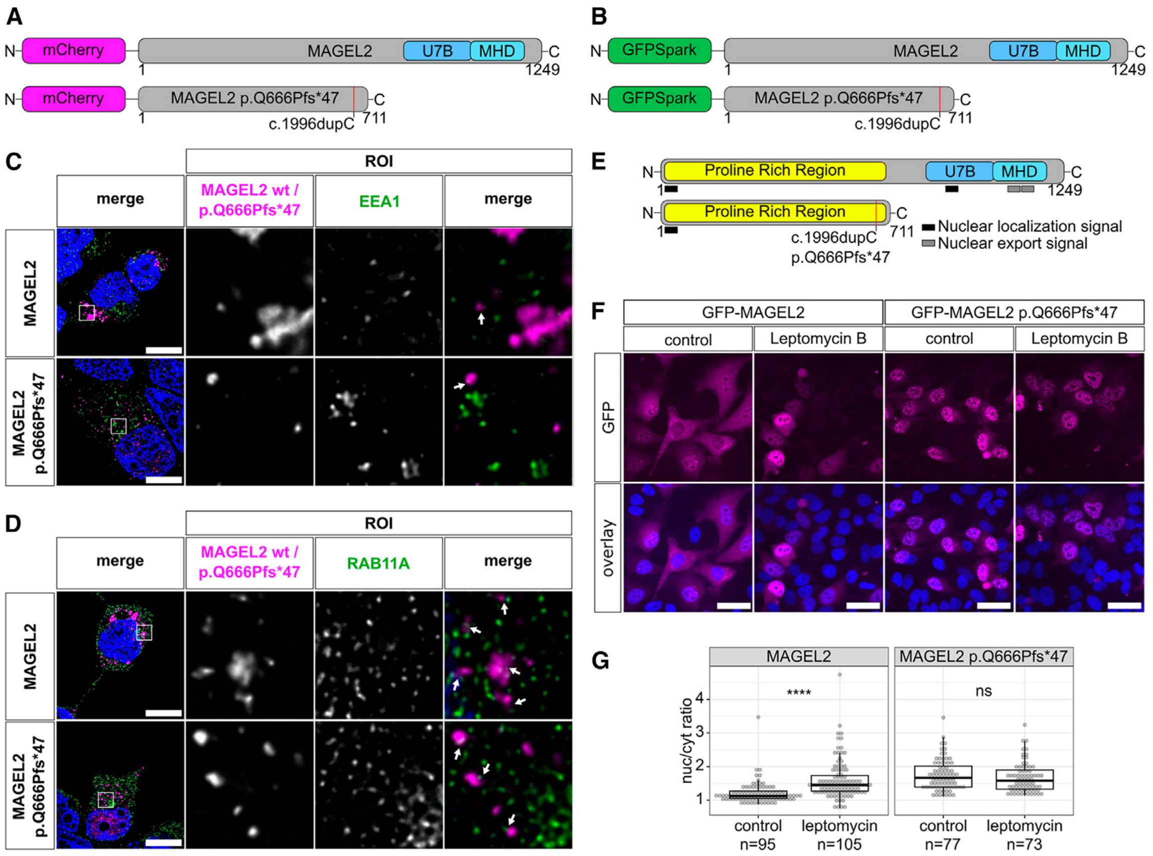

Figure 1. Subcellular localization of MAGEL2 WT and p.Gln666Profs*47

(A and B) MAGEL2/p.Gln666Profs*47 fusion proteins employed for investigation of their subcellular localization, tagged with either an N-terminal mCherry (A) or GFPspark (B).

(C and D) Confocal immunofluorescence microscopy of recombinant fusion proteins WT mCherry-MAGEL2 or mCherry-p.Gln666Profs*47 (magenta) in transiently transfected HEK293T cells, 24 h after transfection. Nuclei were counterstained with DAPI (blue). The scale bar is 10 μm. White arrows indicate sites of spatial proximity between WT MAGEL2 and p.Gln666Profs*47 with endogenous early endosomes (C) and recycling endosomes (D), which were visualized with anti-EEA1 and anti-RAB11A antibodies (green), respectively. At least 5 cells were imaged in three independent replicates and representative cells are depicted.

(E) MAGEL2 WT and truncated p.Gln666Profs*47 with computationally predicted putative NLS (black boxes) and NES (gray boxes) motifs.

(F and G) HeLa cells were transfected with the GFP-tagged wild-type *MAGEL2* or *MAGEL2* c.1996dupC mutant. 48 h after transfection cells were treated with leptomycin B (20 nM) or left untreated for 20 h. Nuclei were counterstained with DAPI (blue).

(F) HeLa cells with and without leptomycin B treatment. Cells from three independent transfection experiments were used for quantification. The scale bar is 40 μm.

(G) Quantification of the nuclear/cytosolic ratio of individual cells from three independent experiments. Wilcoxon tests were calculated employing rstatix.

(WASH regulatory complex),[67–69] the MUST complex is actively recruited to endosomal subdomains by the retromer subunit VPS35.[36,37,66]

Hence, we first addressed a potential co-localization between an mCherry-tagged WT or truncated MAGEL2 protein with the early- and recycling-endosomal markers EEA1 and RAB11A, respectively. Therefore, HEK293T cells were transiently transfected with fluorescently tagged WT and truncated *MAGEL2* expression vectors. WT and truncated MAGEL2 were randomly distributed within the cytoplasm, where the p.Gln666Profs*47 variant predominantly appeared in vesicular-like structures (Figures 1C and 1D). However, both proteins were only rarely in spatial proximity to EEA1 (Figure 1C) but were located adjacent to RAB11A (Figure 1D).

Interestingly, during these analyses we observed a considerably higher nuclear accumulation of the p.Gln666 Profs*47 mutant compared to WT MAGEL2 (Figures 1C and 1D). Based on these findings, we hypothesized that truncated MAGEL2 p.Gln666Profs*47 might be subject to either an increased nuclear import or a reduced nuclear export kinetic in comparison to its wild-type counterpart. Indeed, by employing computational prediction tools, a putative bipartite nuclear localization signal (NLS) motif was indicated, i.e., 4-LSKNLGDSSPPAEAPKPPVYSRPTVLMRAP-33, at the N-terminal end of WT MAGEL2 and p.Gln666 Profs*47 (Figure 1E; Table S6). Additionally, a putative monopartite NLS, i.e., 883-GKATRKKKHLE-893, and two nuclear export signal (NES) motifs, i.e., 1,107-RPKFGLLM VVLSLIF-1,121 and 1,127-VREDLIFNFLFKLGL-1,141, were

predicted within the C-terminal half of WT MAGEL2, which were absent in the truncated p.Gln666Profs*47 variant (Figure 1E, Tables S6 and S7).

To further investigate the nuclear export of MAGEL2 proteins, we transiently transfected HeLa cells with WT GFP-MAGEL2 or p.Gln666Profs*47 expression vectors (Figure 1B) and subsequently blocked the nuclear export of MAGEL2 proteins by inhibiting the export factor karyopherin CRM1 (exportin 1) with leptomycin B.[70,71] As observed in HEK293 cells, also in HeLa cells the p.Gln666Profs*47 variant showed an increased nuclear localization compared to WT MAGEL2 (Figures 1F and 1G). However, some nuclear localization was also observed for WT MAGEL2 (Figures 1C, 1D, and 1F). Consistent with our hypothesis, leptomycin B treatment resulted in a significant accumulation of WT MAGEL2 within the nucleus compared to the untreated control ($W = 2017$, $p = 3.7 \times 10^{-13}$) (Figures 1F and 1G), while truncated p.Gln666 Profs*47 localization was not affected by CRM1 inhibition ($W = 3123$, $p = 0.24$) (Figures 1F and 1G).

In vivo proximity labeling and co-immunoprecipitation reveal protein interaction partners for MAGEL2 WT and the p.Gln666Profs*47 variant

To decipher potential pathogenic roles of MAGEL2 frameshift mutants in SYS-affected individuals, we considered three different hypotheses to link the truncated MAGEL2 proteins to the SYS phenotype: firstly, the different distribution of WT and the truncated MAGEL2 protein might lead to differences in the composition of their interactomes explaining a potential gain or loss of function of MAGEL2 p.Gln666Profs*47 within the nucleus or cytoplasm, respectively. Secondly, the truncated form of MAGEL2, consisting mainly of the intrinsically disordered proline-rich N-terminal region,[40] might *per se* promote interactions with novel binding partners. Lastly, MAGEL2 p.Gln666Profs*47 might still be able to bind canonical interaction partners via its N-terminal region but would lack a functional response due to loss of U7BS and MHD in the C-terminal region.

In a recent study, interacting proteins of over-expressed WT MAGEL2 and the C-terminal portion of MAGEL2 have been identified in HEK293T cells by Sanderson et al.[40] By employing a “subtractive” approach, i.e., comparing proteins binding to full-length MAGEL2 versus proteins binding to the C-terminal portion, also N-terminal protein binding partners were predicted.[40] In contrast, in our study we directly employed the original truncated N-terminal p.Gln666Profs*47 variant and compared its interactome to that of full-length WT MAGEL2 to identify MAGEL2 WT and p.Gln666Profs*47 interaction partners. To achieve this aim, we generated vectors for the expression of biotin ligase miniTurbo[44] (Figure 2A) or GFPspark (Figure 1B) WT MAGEL2 and p.Gln666Profs*47 fusion proteins.

By applying different proteomic approaches, we wanted to cover the entire spectrum of MAGEL2 interaction partners as comprehensively as possible, but also aimed to pinpoint candidates that specifically bind either to the WT or the truncated MAGEL2 proteins. By employing BioID, all proteins in proximity of about 10 nm to the biotin ligase are known to be biotinylated[73] prior to the pull-down. In contrast, by GFP-CoIP, only direct interaction partners and potentially their respective complexes are co-precipitated. Therefore, the interactome identified by BioID was expected to be larger than that obtained by GFP-CoIP (Figure 2B).

MAGEL2 WT and the p.Gln666Profs*47 variant share interactomes involved in RNA stabilization and chromosome condensation

First, we investigated common interaction partners for MAGEL2 WT and p.Gln666Profs*47, which were present in at least two out of three replicates (Figures 2B–2D). Previous analysis of the full-length MAGEL2 interactome suggested that the protein might be involved in various aspects of RNA metabolism.[40] Using BioID and GFP-CoIP, our proteomics analyses revealed 36 common protein interaction partners for WT and truncated MAGEL2 (Figures 2B–2D), of which only YBX1 and PAPBC1 have been previously described to be in proximity to the C-terminal or full-length MAGEL2, respectively.[40] These proteins clustered into two major groups in protein-protein association network analysis[72] (Figure 2C). The first cluster contained PABPC1, YBX1, FUS, ELAVL1, TAF15, and HNRNPA0, which are all implicated in RNA stabilization, while histones H1.0, H1.2, H1.3, H1.4, and H1.10 in the second cluster are involved in chromosome condensation (Figures 2C and 2D). Additionally, HMGA1, SFPQ, TAF15, ILF3, HMGN1, YBX1, and FUS represent a subset of common protein interaction partners involved in the regulation of transcription (Figure 2C; Table S8).

Mutations in MAGEL2 might result in loss of function or gain of function in RNA metabolism processes

Next, we aimed to identify differences in the interactomes of MAGEL2 WT and p.Gln666Profs*47 and analyzed proteins overlapping in GFP-CoIP and BioID for each protein variant (Figure 3A). As expected, fewer proteins were identified employing GFP-CoIP compared to BioID (Figure 3A) due to the general experimental differences between the two approaches, whereby p.Gln666Profs*47 interacted with more proteins than WT MAGEL2 (Figure 3A). The interactome of WT MAGEL2 was mainly found to be involved in chromosome condensation and organization (Figure 3B), whereas mRNA stabilization was one of the top hits of the proteins bound to p.Gln666Profs*47 (Figure 3B).

Subsequently, we focused on 2-fold enriched proteins per pull-down set to identify proteins that were either proximal to or would directly interact with either WT MAGEL2 or p.Gln666Profs*47. Hence, these analyses might also indicate potential novel gain or loss of functions of the truncated MAGEL2 variant (Figure 3C). The enriched proteins identified by the MAGEL2 BioID

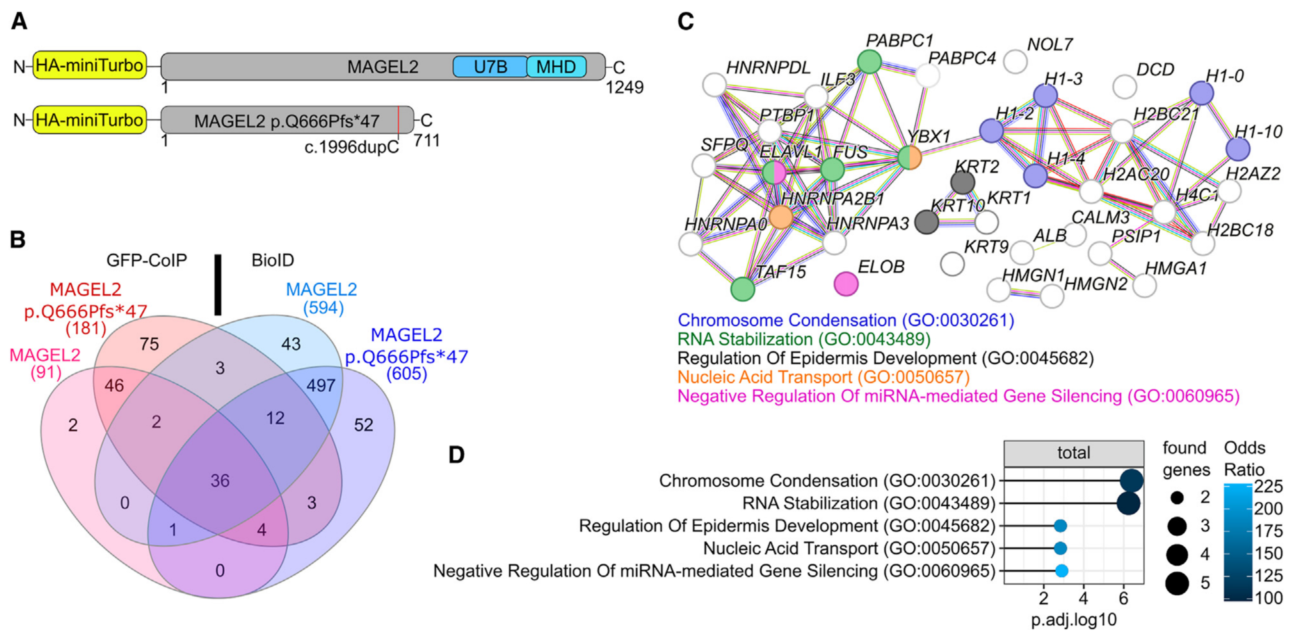

Figure 2. MAGEL2 WT and p.Gln666Profs*47 show high affinity for proteins involved in RNA metabolism and chromosome condensation

(A) Fusion-protein constructs for BioID.

(B–D) Analyses were carried out for proteins identified in at least two of three replicates per experiment.

(B) Combined Venn diagram interaction partners of MAGEL2 WT and p.Gln666Profs*47 identified by BioID and GFP-CoIP.

(C) Protein-protein association networks (gene names are depicted)[72] of the 36 common interaction partners identified in (B), colored by the top 5 biological processes in (D).

(D) Top 5 hits by combined score[60] of gene enrichment analysis employing enrichR for biological processes (2023) in either BioID or GFP-CoIP. Only GO terms with an adjusted p value <0.05 were included.

were mostly involved in transcription regulation (Figures 3C and S1C). In contrast, proteins in proximity to MAGEL2 p.Gln666Profs*47 were implicated in mRNA stabilization and snoRNA metabolism, while direct interaction partners of MAGEL2 p.Gln666Profs*47 identified by LC-MS/MS were involved in cell metabolism, i.e., pyruvate metabolism and gluconeogenesis (Figure 3C; Table S9). Included in that group of proteins was the DNA- and RNA-binding protein KHSRP,[74] which was also enriched in MAGEL2 p.Gln666Profs*47 employing BioID (Figure 3A).

In addition, NUFIP1 was enriched in the WT MAGEL2 BioID (Figure 3C). However, NUFIP1 was on the threshold of detectability in only one of three replicates of western blot analyses upon GFP-CoIP with WT GFP-MAGEL2 (Figure 3D). SMN and FMRP enrichment was observed in the MAGEL2 p.Gln666Profs*47 BioID approach (Figure 3C). Importantly, subsequent western blot analysis confirmed FMRP and SMN to be specifically co-immunoprecipitated with WT GFP-MAGEL2 and GFP-p.Gln666 Profs*47 (Figure 3D). In this context, FMRP showed a higher affinity to WT MAGEL2 in comparison to the truncated protein (Figure 3D). Hence, our data strongly indicate that FMRP and SMN are indeed proximal interaction partners of WT and truncated MAGEL2 proteins, whereas NUFIP1 is most likely an indirect binding partner of WT MAGEL2 (Figures 3C and 3D).

USP7, which has been previously described as a functional binding partner of MAGEL2,[66] was found to be enriched in the WT MAGEL2 GFP-CoIP in both LC-MS/MS and western blot analyses (Figures 3A and 3B). Intriguingly, USP7 was also slightly detectable in the MAGEL2 p.Gln666Profs*47 variant (Figure 3B), although the annotated U7BS is absent in the truncated mutant (Figure 2A).[36]

MAGEL2 WT and p.Gln666Profs*47 co-localize with SMN and FMRP in mouse N2a cells

Considering that WT and truncated MAGEL2 variants were specifically binding to SMN and FMRP in our GFP-CoIPs (Figure 3D), we next asked the question of whether both MAGEL2 variants colocalized with SMN and FMRP in neuronal cells. To that aim, we transiently transfected the mCherry-MAGEL2 and p.Gln666Profs*47 constructs into mouse N2A cells (Figures 4A and 4B). In the majority of the transfected cells, we observed colocalization of both MAGEL2 variants with SMN and FMRP in distinct speckles present in the cytoplasm. Analogous to the experiments presented before in HEK293T cells (Figures 1B and 1C), we also investigated whether MAGEL2 variants were found on endosomes in N2A cells. Indeed, transiently transfected mCherry-MAGEL2 and p.Gln666Profs*47 partially co-localized with EEA11 and RAB11A (Figures 4C and 4D). Interestingly, the association of MAGEL2 with early and recycling endosomes appears preferentially in neuronal cells

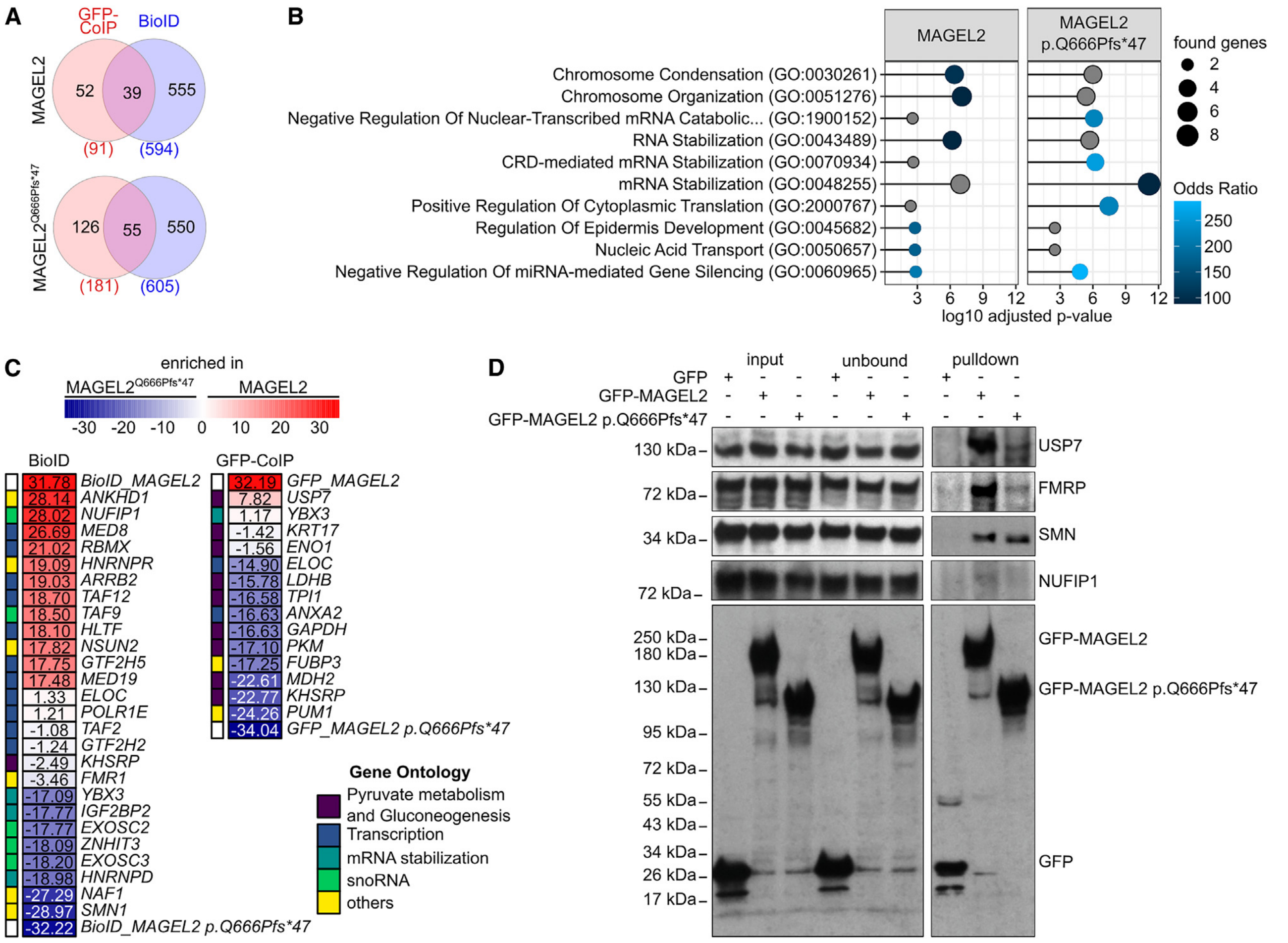

Figure 3. MAGEL2 WT and p.Gln666Profs*47 interact with proteins involved in RNA metabolism and gene expression as well as disease related SMN (spinal muscular atrophy) and FMRP (fragile-X syndrome)

(A) Venn diagrams of protein interaction partners for MAGEL2 WT and p.Gln666Profs*47 identified by BioID or GFP-CoIP.

(B) Gene enrichment analysis for Biological Processes (2023) in either BioID or GFP-CoIP employing enrichR. Shades of blue depict top 5 hits by combined score.[60] Gray circles indicate that the respective GO term is among the top hits in another condition. GO terms with an adjusted p value > 0.05 were excluded.

(C) Heatmap for enriched proteins (gene names are depicted) in MAGEL2 WT and p.Gln666Profs*47 by BioID and GFP-CoIP. GO term enrichment analyses for Biological Process and KEGG Pathways from Figure S1 were grouped to broader functional terms as shown in Table S9 are written in the respective colors (Gene Ontology). Gene ontology "others" include novel interaction partners of interest which could not be assigned to the remaining annotated terms.

(D) Western blots of proteins which co-immunoprecipitated with WT GFP-MAGEL2 or GFP-p.Gln666Profs*47 ($n = 3$). 5% of the lysate were used in input fractions.

and was less pronounced in HEK293T cells (Figures 1C and 1D). Of note, p.Gln666Profs*47 showed less overlap with RAB11A compared to WT MAGEL2 (Figure 4B).

Protein interactomes of MAGEL2 and the *SNORD116* snoRNA

Due to the phenotypic similarities between SYS and PWS and the genomic proximity of the respective affected genes, i.e., *MAGEL2* and *SNORD116*,[1] we also sought to find a functional link between MAGEL2 and *SNORD116* snoRNAs. Therefore, we employed an RNA-centric based pull-down approach, employing 3′-biotinylated *SNORD116* (copy 3), the canonical *SNORD100* snoRNA, and a scrambled RNA control (SCR) followed by LC-MS/MS or western blot analyses for identification of protein interaction partners (Figure 5).

From these analyses, a direct interaction between *SNORD116* and WT MAGEL2 or p.Gln666Profs*47 was not corroborated since WT and mutant MAGEL2 were present in all co-IP samples including *SNORD100* and *SCR* controls, which might indicate a more general affinity of mutant and WT MAGEL2 to various RNA species (Figure S2). Because *SNORD100* and the *SCR* control sample were found to be more efficiently 3′-biotinylated than *SNORD116* (Figure S3), we decided to analyze proteins present in at least two of three experiments by LC-MS/MS and subtracted proteins identified in the *SCR* sample. Thereby, we aimed to exclude non-specific RNA binding proteins (Figure 5A).

GO term analysis revealed that box C/D snoRNP assembly and ribosome biogenesis are within the top 5 GO biological processes of the 97 proteins bound to *SNORD116* and canonical *SNORD100* and validated our pull-down

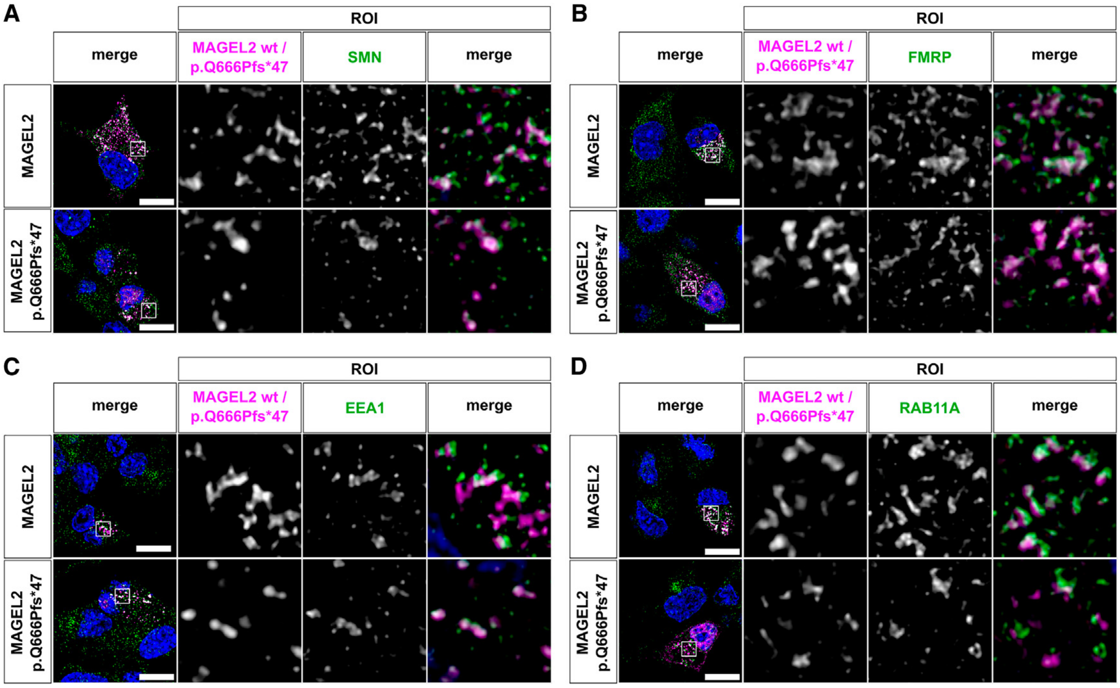

Figure 4. MAGEL2 WT and p.Gln666Profs*47 co-localize with EEA1, RAB11, SMN and FMRP in mouse Neuro-2a cells
Mouse Neuro-2a cells were transiently transfected with WT mCherry-MAGEL2 or mCherry-p.Gln666Profs*47 (magenta) fusion proteins and subcellular localization was analyzed employing confocal immunofluorescence microscopy 24 h after transfection. Nuclei were counterstained with DAPI (blue). The scale bar is 10 μm. SMN (A), FMRP (B), endogenous early endosomes (C), and recycling endosomes (D) were visualized with anti-SMN, anti-FMRP, anti-EEA1, and anti-RAB11A antibodies (green), respectively. A white signal in the overlap images indicated signal overlap or co-localization. At least 3 transfected cells were imaged in three independent replicates and representative cells are depicted.

approach (Figure 5A and 5B). Moreover, GO term analysis of co-immunoprecipitated proteins from either *SNORD100* or *SNORD116* RNA-CoIPs resulted in the same top GO terms (Figure 5B and 5C), which indicates that orphan *SNORD116* snoRNAs assemble into canonical snoRNPs.

To more precisely pinpoint specific *SNORD116* protein interaction partners, the RNA co-IP was also performed employing mouse N2a cell lysates (Figure S4). Among the ten common interaction partners co-immunoprecipitated with both snoRNAs (*SNORD116* and *SNORD100*) in both cell types, we identified box C/D snoRNP assembly proteins NUFIP1, RUVBL1, and RUVBL2 (Figures S4A and S4C).[75] In contrast, specific *SNORD116* interactors, overlapping in human and mouse, were identified as MRPL1, WDR82, IGHMBP2, KHDRBS1, STRAP, and NXF1 (Figures S4A and S4B). In summary, our data did not indicate an overlapping *SNORD116* and MAGEL2 interactome, as no common proteins could be identified by the MAGEL2 BioID and GFP-CoIP and by the *SNORD116* RNA-CoIP (Figures 5C and 5D).

Mutations in MAGEL2 reduce expression of *SNORD116* snoRNAs in SYS proband cell lines

Even in the absence of a common interactome between MAGEL2 and *SNORD116* snoRNAs, our results do not necessarily exclude a common pathophysiological mechanism between SYS and PWS. Since we hypothesized a potential involvement of MAGEL2 in transcriptional regulation and RNA stabilization, we assessed the influence of truncated MAGEL2 mutants on the expression of *SNORD116* snoRNAs involved in the etiology of PWS.[6–10] Hence, we employed RT-qPCR and northern blot analysis to investigate the abundance of *SNORD116* in small molecule neural progenitor cells (smNPCs) derived from healthy and SYS iPSCs, paternally harboring the WT or mutant (c.1996dupC or c.1802delC) *MAGEL2* alleles, respectively.

Indeed, RT-qPCR analysis revealed that expression of *SNORD116* ($W = 110$, $p = 0.0101$) (Figure S5A), as well as *SNORD115*, and of *SNORD109A* snoRNAs were downregulated by 31%, 42%, and 28% in smNPCs, respectively (Figure S5B). Collectively, the abundance of these box C/D snoRNAs within the PWS gene locus was about 32% lower in SYS smNPCs ($W = 313$, $p = 0.000254$) (Figure 6A). Because of the high sequence similarity of the different *SNORD116* and *SNORD115* snoRNA copies within their respective gene location,[6] we were able to apply RT-qPCR analysis only to three or one copy of the *SNORD116* and *SNORD115* clusters, respectively (Figures 6A, S5A, and S5B). Hence, we also investigated abundances of both snoRNAs by northern blot analysis employing specific probes which detected multiple copies of *SNORD116* (19 of 30) and *SNORD115* (30 of 49) (Figure 6C). In agreement with qPCR analysis, quantification of northern blot signals

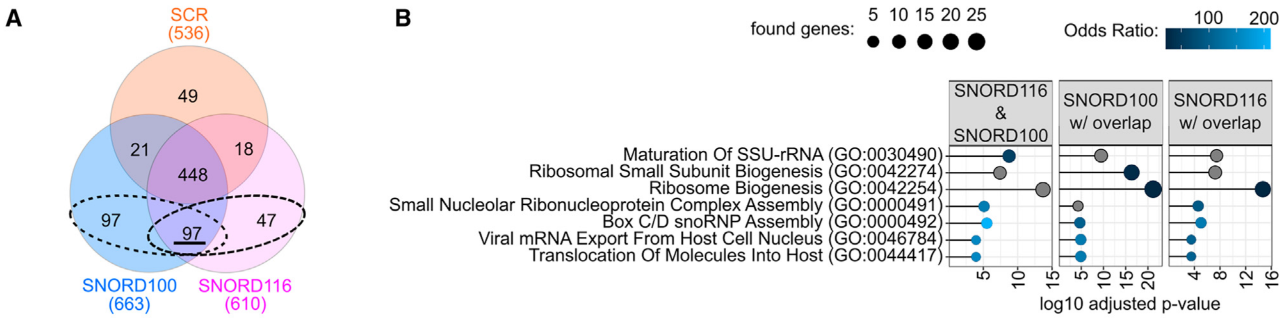

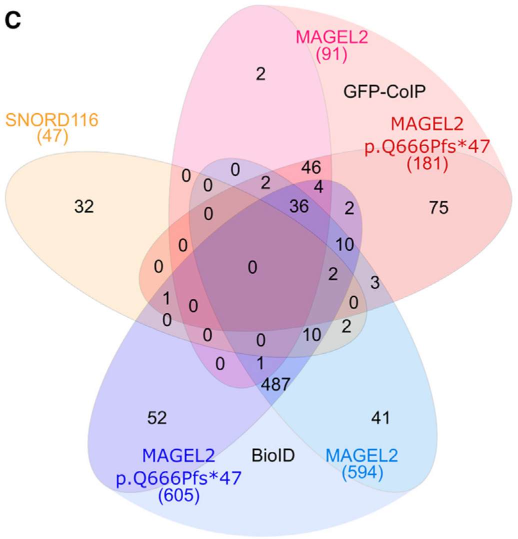

D

Protein	GFP-CoIP		BioID	
	MAGEL2	MAGEL2 p.Q666Pfs*47	MAGEL2	MAGEL2 p.Q666Pfs*47
SLX9		✓		✓
SF1<br>KHDRBS1		✓	✓	✓
SF3B2 | RPL15 | SPOUT1 | RBM39 | PRC1 | EIF2S2 | SAFB | PRPF40A | PHF5A | TAF2			✓	✓
ANKHD1<br>NSUN2			✓	

Figure 5. ***SNORD116* does not directly interact with WT MAGEL2 or p.Gln666Profs*47**
RNA-coIP was performed with biotinylated *SNORD116*, *SNORD100*, or a *SCR* control with HEK293T IP lysates. Analyses were carried out with proteins identified in at least 2 of 3 replicates per condition.
(A) Venn diagrams showing overlapping proteins identified via RNA-coIP. The 97, 194, and 144 proteins found in RNA-coIPs for *SNORD116* and *SNORD100* (underlined), SNORD100 with overlap (dashed circles), and SNORD116 with overlap (dashed circles) were employed for the analysis in (B).
(B) GO term enrichment analysis employing enrichR for Biological Processes (2023) for proteins identified in *SNORD116* and *SNORD100*, but not in the *SCR* control. Top 5 hits by Combined Score[60] are shown in shades of blue. Gray circles indicate that the respective GO term is among top hits in another condition. Only GO terms with an adjusted p value < 0.05 were included.
(C) Venn diagram displaying the overlap between unique *SNORD116* interacting proteins (A) and those identified in 2 of 3 replicates of the BioID or GFP-CoIP performed with WT MAGEL2 or p.Gln666Profs*47.
(D) Table showing overlapping proteins from (C).

(relative to 5S rRNA and U6 snRNA) showed reduced abundancies of *SNORD116* and *SNORD115* in SYS smNPCs (Figure 6D).

Because of their reduced abundance in SYS proband cell lines, we investigated whether lower transcript levels of *SNORD116* and *SNORD115* might be due to aberrant processing from their host transcript in SYS cell lines. To address this, we designed primers targeting an amplicon ranging from within the respective snoRNA into the intronic region and performed RT-qPCR analysis (Figure 6B). Relative expression of pre-processed PWS box C/D snoRNAs was decreased in SYS compared to healthy smNPCs (−19.1%, W = 41, p = 0.263) (Figure 6A). Importantly, pre-processed snoRNAs had a similar relative expression compared to their mature snoRNAs (Figure S5B), indicating altered transcription or RNA stabilization rather than deregulation of snoRNA processing.

In contrast to the expression pattern of snoRNAs located within the PWS locus, RNA levels of canonical box C/D snoRNAs, including *SNORD32* located on chromosome 19q13 within *RPL13A*,[76] remained unaltered (+3%, W = 46, p = 0.215) (Figure 6A). Interestingly, the abundance of the canonical box C/D snoRNA *SNORD100* was even higher in SYS smNPCs than in healthy control cell lines (Figure S5B).

We also examined the expression levels of protein-coding genes located within the PWS critical region on chromosome 15q11–q13, including the *MAGEL2* mRNA transcript. As observed for snoRNAs, the abundance of protein-coding gene expression within the chromosome 15q11–q13 locus was significantly lower in SYS smNPCs (W = 185, p = 0.0105). Nevertheless, regulation of single genes within the PWS locus varied strongly, i.e., the abundances of *SNRNPN* and *MKRN3* were reduced by about 25%

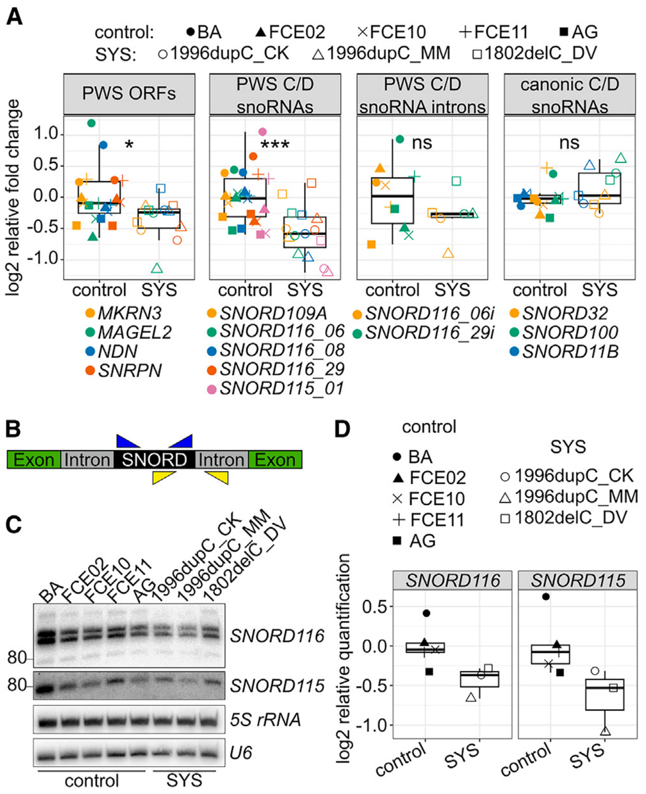

Figure 6. Influence of *MAGEL2* frameshift mutations on gene expression, assessed in SYS smNPCs compared to healthy controls

(A) RT-qPCR analysis of genes of interest (GOI) within and outside chromosome 15q11–q13. Relative expression was calculated with primer efficiency normalized N0 values (linRegPCR[50]). N0 values were normalized to the geometric mean of housekeeping genes *TBP*, *GAPDH*, and *HPRT* and to the geometric mean of healthy control samples. Genes were combined into groups depending on type and genomic localization. Wilcoxon tests were applied employing rstatix.

(B) Scheme of primer position to detect snoRNAs before (yellow) and after (blue) splicing and processing.

(C) Northern blot analysis of 19 and 30 copies of *SNORD116* and *SNORD115*, respectively.

(D) Northern blot bands were quantified employing Fiji and normalized to geometric mean areas of *5S rRNA* and *U6* snRNA signals.

whereas the abundance of *NDN* (−2%) or *MAGEL2* (−8%) was unchanged or only slightly lower, respectively (Figure S5B).

We finally addressed the question of whether in smNPCs *MAGEL2* c.1996dupC and *MAGEL2* c.1802delC mRNAs might exhibit different sizes since both frameshift mutants harbor elongated 3′ UTRs of more than 2 kb (in contrast to 433 bases in the WT transcript). We hypothesized that the abundance of the intronless mutant *MAGEL2* transcripts would be decreased due to EJC-independent nonsense-mediated decay (NMD). This control mechanism assures the degradation of mRNAs exhibiting a premature stop codon resulting in unusually long (∼1 kb) and unstructured 3′ UTRs.[77] However, by northern blot analysis, employing probes hybridizing either to the 3′ or 5′ end of the *MAGEL2/MAGEL2* c.1996dupC transcripts, we showed that *MAGEL2* c.1996dupC and *MAGEL2* c.1802delC mRNAs are not subject to NMD. These findings are consistent with mRNA sizes of MAGEL2 mutants being indeed identical to that observed for WT *MAGEL2* mRNA (Figure S6).

Discussion

Since the discovery of truncated MAGEL2 protein variants and their involvement in the etiology of the neurodevelopmental disorder SYS,[30] most of the past research has focused on their molecular functions and their involvement in disease.[36,40,66,78,79] Due to the phenotypic similarities between SYS and PWS,[1] we sought to elucidate possible links between both diseases at a molecular level, i.e., to link *MAGEL2* mutations to genes involved in PWS.

Our data show an accumulation of the predominant p.Gln666Profs*47 variant within the nucleus in HEK 293T and HeLa cells, whereas WT MAGEL2 accumulated within the nucleus only upon CRM1 inhibition. This suggests that WT MAGEL2 is dynamically shuttled between the cytoplasm and the nucleus. These observations imply functions of MAGEL2 within the nucleus and are corroborated by our proteomics data, which link MAGEL2 to regulators of transcription. This also suggests yet undescribed functions of both protein variants within the nucleus. Previously, the group by Urreizti and coworkers described a predominant nuclear localization of a truncated MAGEL2 variant.[80] In another study, Centeno-Pla et al.[81] have recently reported a predominant nuclear localization of six truncated MAGEL2 variants. Among these, the variants with the strongest nuclear accumulation associated with arthrogryposis multiplex congenita (AMC [MIM: 618947]), indicating a genotype-phenotype correlation dependent on the level of nuclear localization of truncated MAGEL2.[81] In summary, the above findings are thus consistent with increased nuclear localization of all truncated MAGEL2 mutants investigated so far due to the loss of the predicted C-terminal NES motifs upon truncation of the protein.

In the cytoplasm of HEK293T cells, mCherry-tagged MAGEL2 WT and p.Gln666Profs*47 were found to be adjacent to endogenous RAB11A-positive recycling endosomes, whereas only a small fraction of both proteins was found proximate to endogenous EEA1-positive endosomes. In addition, we observed the truncated MAGEL2 protein to be organized in vesicular-like structures in the cytosol. In contrast, in N2A cells, both MAGEL2 variants showed a strong colocalization with EEA1 and RAB11A indicating that the mechanisms controlling the subcellular localization of MAGEL2 might be partially cell type specific. A recent study showed that F-actin and ArpC5 were reduced in retromer subunit VPS35-positive endosomes in PWS and SYS stem cell-derived neurons.[79] Together with our data, this indicates that the N-terminal

region of MAGEL2 might be required for recognition and binding by VPS35, which also recruits the SHRC complex through an interaction with FAM21.[36] Hence, our findings indicate that MAGEL2 association with the retromer might persist even in the presence of the truncated MAGEL2 mutants, thereby displaying compromised MUST assembly (i.e., loss of the functional U7BS and MHD regions).

Since SYS phenotypes are not resembled by a complete loss of the *MAGEL2*,[32,82,83] a few possible scenarios have been raised in the past to explain the SYS disease. On the one hand, it was proposed that transcription of the imprinted maternal allele might become leaky upon loss of the paternal allele.[1,32,84] On the other hand, truncated proteins have been linked to gain-of-function phenotypes[85,86] and also neomorphic effects have been proposed for truncated MAGEL2 variants.[1] Consistent with these observations, our results suggest gain or loss of cellular functions of truncated MAGEL2 mutants within the nucleus or the cytoplasm, respectively. In addition, WT MAGEL2 dynamically shuttles between the cytoplasm and the nucleus, and therefore might indicate novel functions within the nucleus.

MAGEL2 is an intrinsically disordered protein (IDP) exhibiting over 85% of intrinsically disordered protein regions (IDPR).[40] Previous studies have shown that highly structured proteins are related to integral membrane proteins or enzymes, while signaling and regulation—including differentiation, transcription regulation, DNA condensation, and mRNA processing—are mainly orchestrated by intrinsically disordered proteins.[87,88]

Sanderson and coworkers have conducted a biotin proximity-labeling approach employing HEK293 cells stably expressing the full-length or a C-terminal MAGEL2 mutant.[40] In these analyses they have demonstrated that the N-terminal portion of MAGEL2 might be implicated in RNA metabolism processes. In a “subtractive” approach, they assigned hits that were found in full-length but not the C-terminal MAGEL2 conditions as N-terminal interaction partners.

By studying interaction partners of MAGEL2 WT and p.Gln666Profs*47 employing BioID and CoIP, our GO term analysis revealed their potential function in RNA stabilization as well as chromosome condensation. Taken together, these data suggest that the N-terminal half of MAGEL2 might be required for the interaction with regulators of RNA metabolism.[40] Association of KHSRP and FUBP3 with the N-terminal protein portion of MAGEL2 were shown in the present study and by Sanderson et al.[40] The absence of U7BS and MHD might lead to loss of function of the pathogenic truncated MAGEL2 variants, for example through the deregulation of targeted proteins by ubiquitination or de-ubiquitination.

The deubiquitinase USP7 was found as an interactor of MAGEL2 WT and the p.Gln666Profs*47 variant, confirming the direct binding of those proteins as part of the MUST complex.[66] The interaction is decreased in p.Gln666Profs*47 compared to WT MAGEL2, which suggests that part of the SYS phenotype could potentially be related to decreased association of these two proteins. This is particularly interesting, as heterozygous loss-of-function variants of *USP7* are the cause of Hao-Fountain syndrome (HAFOUS [MIM: 616863]), a neurodevelopmental disorder that clinically manifests developmental delay/intellectual disability, autism spectrum disorder, muscular hypotonia.[89] This suggests that the two genes and conditions represent not only a molecular but also a pathophysiological and clinical continuum.[89]

Some proteins, such as ENO1, GAPDH, PKM, and LDHA, have been previously identified as binding partners for the C-terminal MAGEL2 variant,[40] while in our study these proteins were enriched in GFP-MAGEL2 p.Gln666Profs*47 by co-immunoprecipitation. Discrepancies between the results from the two studies might arise from the different experimental setups. Sanderson and colleagues[40] generated a C-terminal MAGEL2 variant which exhibited a 62 amino acid overlap with the C terminus of the truncated MAGEL2 p.Gln666Profs*47 protein. Hence, this portion of the protein might harbor binding sites for the above-mentioned proteins.

Based on our study, it is unlikely that MAGEL2 and *SNORD116* share a common interactome or directly form an RNP complex. Still, we were able to identify interaction partners for both MAGEL2 variants as well as for *SNORD116*. WT and truncated MAGEL2 were found both as potential interaction partners of SMN, FMRP, KHSRP, and FUS. Intriguingly, SMN was reported to associate with FMRP,[90,91] KHSRP,[92] and FUS.[90,93,94]

SMN has been shown to shuttle between the cytoplasm and the nucleus,[95] which we also observed for the WT MAGEL2 protein, and has been implicated in snRNP assembly, mRNA trafficking, and local translation, and was also observed to influence mitochondria and bioenergetic pathways.[93] Mutations or the homozygous loss of *SMN1* have been reported to be the main cause of spinal muscular atrophy (SMA [MIM: 253300]).[96–98]

Silencing of *FMR1* by DNA methylation of expanded CGG triplet repeats within the 5′ UTR leads to the loss of FMRP and causes the fragile X syndrome (FXS [MIM: 300624]).[99,100] FMRP (fragile-X-messenger ribonucleoprotein) is an RNA-binding protein, which was initially shown to inhibit translation *in vitro* and in *Xenopus laevis* oocytes.[101] It was recently described to regulate activity-dependent local translation in CA1 neurons by repressing translation[102] and thereby is a crucial regulator in neuronal mRNA metabolism, neuronal development, axonal growth, and synaptic plasticity.[103,104]

KHSRP (also designated as FUBP2) functions as a DNA- and RNA-binding protein which was previously described to be involved in degradation of AU-rich element (ARE)-containing mRNAs and splicing.[74,105,106] Recent findings by Olguin and colleagues indicated that KHSRP is a regulator of brain development and function.[74] Therefore, MAGEL2 might be an integral component of these respective complexes.

Due to phenotypic similarities, SMA should be considered as a differential diagnosis for SYS[33] and PWS.[107] Moreover, intellectual disability, behavioral symptoms, and autism spectrum disorder are shared symptoms of FXS[99,100] and SYS.[1,31–33] It is noteworthy that there are also case reports of individuals with FXS exhibiting a Prader-Willi phenotype (i.e., hyperphagia and obesity), which was likely caused by reduced cytoplasmic FMRP interacting protein (CYFIP1) expression.[108] Vice versa, haploinsufficiency of *Cyfip1* and *Fmr1* knockout mice have been shown to share a common phenotype.[109] Interestingly, heterozygous loss of *CYFIP1* appears in individuals with type I deletions of the PWS critical region, underlining the complexity between the phenotypic similarities of PWS and FXS.[109] Consequently, downstream molecular pathways of the potential MAGEL2 targets FMRP and SMN might be negatively regulated in SYS-affected individuals.

Besides modulating the (de-)ubiquitination of WASH,[36] MAGEL2 has also been shown to control the circadian rhythm via cryptochrome 1 (CRY1) stabilization mediated by USP7-dependent de-ubiquitination.[78] In addition, previous studies revealed that the ubiquitin proteasome system (UPS) targets and degrades SMN[110] and FMRP through a phosphorylation-induced ubiquitination cascade.[111] Together with our data on the specific co-immunoprecipitation and co-localization of SMN and FMRP with WT and truncated MAGEL2 variants, we suggest a model where MAGEL2 regulates the stability of both proteins. Future studies should examine whether the truncation of MAGEL2 leads to decreased levels of SMN and FMRP. This might indeed explain some of the common phenotypes observed between individuals with SYS and FXS[1,31–33,99,100] or between individuals with SYS and SMA.[33]

Utilizing RNA-CoIP, we were able to identify protein interaction partners for *SNORD116* RNAs which differed from canonical *SNORD* snoRNA proteins.[14] In these analyses, IGHMBP2 and STRAP specifically co-immunoprecipitated with *SNORD116*. Mutations in IGHMBP2 are linked to spinal muscular atrophy, type 1 (SMARD1 [MIM: 604320]),[112–117] which shows symptoms such as hypotonia, feeding problems, and poor suck.[118] In addition, more than 30% and 50% of individuals with PWS die of respiratory failure[119] or respiratory diseases,[120] respectively. This links PWS to SMARD1, where life-threatening respiratory distress/respiratory failure was shown to appear within 13 months of age.[112]

MAGEL2 WT and p.Gln666Profs*47 both interacted with RNA stabilization associated factors YBX1, ELAVL1, TAF15, PABPC1, and FUS. RNA stabilizers function mainly in post-transcriptional control of RNAs, ranging from splicing, polyadenylation, mRNA stabilization, and mRNA localization to translation.[121] For example, the m^{5}C reader YBX1 recruits ELAVL1 and thereby preserves its target mRNA from degradation.[122] Strikingly, m^{5}C was reported as an important dynamically regulated RNA modification during brain development and is enriched in genes for synaptic plasticity regulation.[123] Our data thus indicate that MAGEL2 might also act as an essential component in RNA stabilization, possibly by fine-tuning the interaction of the m^{5}C reader YBX1 with ELAVL1 and thereby affecting neuronal plasticity. Interestingly, ELAVL1 was also found to bind to *SNORD115* from the PWS critical region.[124]

By linking of MAGEL2 to gene expression and RNA stabilization, we hypothesized that mutations in *MAGEL2* might lead to altered gene expression or transcript abundance in SYS smNPCs (exhibiting c.1996dupC and c.1802delC mutations), in particular within the PWS critical region. Indeed, we observed reduced transcript abundances of most genes from the PWS region, in particular *SNORD116* (i.e., a reduction by about 31%). In addition, RNA levels of *SNORD115*, *SNORD109A*, *MKRN3*, and *SNRPN* were also decreased. A fairly moderate reduction of transcript abundancies for RNA transcripts from the PWS region might not be considered sufficient for the observed phenotypes in SYS. However, as demonstrated in the case of miRNAs, downregulation of expression of their mRNA targets has been reported to be within a similar range.[125] This indicates that even moderate changes in mRNA expression can result in severe disease phenotypes, in particular if several genes are affected. In addition, the moderate reduction of *SNORD116* abundance in probands with SYS, compared to individuals with PWS, might explain at least some of the missing clinical features not observed in SYS, such as severe obesity.

In contrast to the above genes, abundances of *NDN* from the PWS critical region and canonical box C/D snoRNAs located outside the PWS region were found not to be affected in SYS smNPCs. The observed reduced abundance of transcript levels of genes from the PWS critical region might thus arise, for example, from an aberrant interaction of mutant MAGEL2 proteins with regulators of transcription or RNA stability.

MAGEL2 mRNA levels differed only slightly between SYS and healthy smNPCs. Together with the identification of novel protein interaction partners, the stability of the truncated MAGEL2 mRNA in SYS proband cell lines as well as its expression in transfection experiments underline that this variant likely leads to gain-of-function effects in probands with SYS, as also suggested previously.[1] We thus propose that the preferential sequestration of the truncated variant to the nucleus plays a fundamental role in the pathophysiology of the disease.

Recent case reports have described individuals with PWS exhibiting atypical microdeletions within 15q11–q13 whose *SNORD116* cluster was not deleted.[126,127] In contrast, several previous studies have reported that an entire deletion of the *SNORD116* cluster was sufficient to cause PWS.[6–10] These contradictory case reports underline the complexity of genes involved in the etiology of PWS. Our study demonstrates functions of MAGEL2 in the regulation of RNA metabolism, especially of genes within the PWS critical region. It thus might be conceivable that in

PWS-affected individuals with atypical microdeletions which do not result in the deletion of the *SNORD116* cluster,[126,127] the expression of *SNORD116* might still be deregulated. Therefore, we propose that examination of *SNORD116* transcript abundance by RT-qPCR might be included as a tool for the diagnosis of PWS in the future, e.g., from NPCs differentiated from fibroblasts derived from individuals with PWS.

Conclusion

In summary, we have provided evidence that MAGEL2 shuttles between the cytoplasm and the nucleus and that it interacts with proteins involved in transcription regulation and RNA metabolism. Consistent with these findings, we observed the abundances of genes expressed from the PWS critical region, including *SNORD116*, to be reduced in SYS smNPCs harboring the c.1996dupC and c.1802delC *MAGEL2* mutations. Hence, MAGEL2 might indeed be involved in the transcription of genes from the PWS locus thus resembling some but not all features of PWS. In addition, among other proteins, we identified SMN and FMRP proteins as binding partners for MAGEL2 variants, both causing diseases linked to known phenotypic features of individuals with SYS or PWS. Hence, although the precise roles of *SNORD116* and MAGEL2 protein complexes and their pathways in the etiology of SYS and PWS yet remain to be fully determined, our data provide a first step in the elucidation of their role in these pathologies, in particular in their relation to other motor neuron diseases. A perturbed interaction of mutant MAGEL2 with interaction partners such as SMN and FMRP and/or its influence on *SNORD116* expression might thus explain some of the observed phenotypes in SYS and PWS diseases. Hence, our study sets the stage for further investigation into the molecular mechanisms of both diseases and might aid in the generation of therapeutic drugs for future therapeutic options.

Data and code availability

The datasets generated during this study are available via figshare (doi: https://doi.org/10.6084/m9.figshare.c.7217859). If there is interest in the raw Airyscan immunofluorescence images, please contact the corresponding author.

Supplemental information

Supplemental information can be found online at https://doi.org/10.1016/j.ajhg.2024.05.023.

Acknowledgments

This research was funded by the Austrian Science Funds (FWF ZFP326120 and FWF TAI 411-B) to A.H. We would like to thank Dr. Martin Offterdinger (Institute of Biooptics, Medical University of Innsbruck) for his technical support. We also thank Beate Abt and Alexander Kühbacher (Institute of Molecular Biology, Medical University of Innsbruck) for technical support. We thank Matthias Erlacher, Utz Fischer, and Andreas Janecke for helpful discussions and critically reading the manuscript. We thank Anna and her parents for inspiration to initiate this study. We dedicate this paper to Jürgen Brosius, on the occasion of his 75th birthday, and Harry Noller, both leading pioneers in RNA research.

Author contributions

A.H., conceptualization, funding acquisition, supervision, writing – review & editing, project administration, validation; D.H., investigation, writing – original draft, formal analysis, data curation, visualization, validation, writing – review & editing; A.V., investigation, validation, writing – review & editing; A.E., resources, writing – review & editing; A.S., resources, writing – review & editing; M.S.-C., resources, writing – review & editing; H.F., validation, writing – review & editing; V.R., investigation, verification, writing – review & editing; M.S., resources, writing – review & editing; C.P.S., resources, writing – review & editing; L.A.H., validation, writing – review & editing; L.K., resources, writing – review & editing; B.S., resources, writing – review & editing; F.E., resources, writing – review & editing; S.G., validation, writing – review & editing; M.E.G.d.A., validation, writing – review & editing. All authors contributed to editing and revising and approved the final version manuscript.

Declaration of interests

The authors declare no competing interests.

Received: April 16, 2024
Accepted: May 29, 2024
Published: June 21, 2024

Web resources

enrichR, https://cran.r-project.org/web/packages/enrichR/index.html
GenBank, https://www.ncbi.nlm.nih.gov/genbank/
Ggpubr, https://CRAN.R-project.org/package=ggpubr
OMIM, https://www.omim.org/
R, https://www.R-project.org/
R Studio, http://www.rstudio.com/
Rio, https://cran.r-project.org/web/packages/rio/index.html
rstatix, https://CRAN.R-project.org/package=rstatix

References

1. Fountain, M.D., and Schaaf, C.P. (2016). Prader-Willi Syndrome and Schaaf-Yang Syndrome: Neurodevelopmental Diseases Intersecting at the MAGEL2 Gene. Dis. Basel Switz. *4*, 2. https://doi.org/10.3390/diseases4010002.
2. Chamberlain, S.J. (2013). RNAs of the human chromosome 15q11-q13 imprinted region. Wiley Interdiscip. Rev. RNA *4*, 155–166. https://doi.org/10.1002/wrna.1150.
3. Höybye, C., and Tauber, M. (2022). Approach to the Patient With Prader–Willi Syndrome. J. Clin. Endocrinol. Metab. *107*, 1698–1705. https://doi.org/10.1210/clinem/dgac082.
4. Cassidy, S.B., Schwartz, S., Miller, J.L., and Driscoll, D.J. (2012). Prader-Willi syndrome. Genet. Med. *14*, 10–26. https://doi.org/10.1038/gim.0b013e31822bead0.

5. Szabadi, S., Sila, Z., Dewey, J., Rowland, D., Penugonda, M., and Ergun-Longmire, B. (2022). A Review of Prader–Willi Syndrome. Endocrines *3*, 329–348. https://doi.org/10.3390/endocrines3020027.
6. Cavaillé, J. (2017). Box C/D Small Nucleolar RNA Genes and the Prader-Willi Syndrome: A Complex Interplay*8* (Wiley Interdiscip. Rev. RNA), pp. e1417. https://doi.org/10.1002/wrna.1417.
7. Tan, Q., Potter, K.J., Burnett, L.C., Orsso, C.E., Inman, M., Ryman, D.C., and Haqq, A.M. (2020). Prader–Willi-Like Phenotype Caused by an Atypical 15q11.2 Microdeletion. Genes *11*, 128. https://doi.org/10.3390/genes11020128.
8. Sahoo, T., del Gaudio, D., German, J.R., Shinawi, M., Peters, S.U., Person, R.E., Garnica, A., Cheung, S.W., and Beaudet, A.L. (2008). Prader-Willi phenotype caused by paternal deficiency for the HBII-85 C/D box small nucleolar RNA cluster. Nat. Genet. *40*, 719–721. https://doi.org/10.1038/ng.158.
9. de Smith, A.J., Purmann, C., Walters, R.G., Ellis, R.J., Holder, S.E., Van Haelst, M.M., Brady, A.F., Fairbrother, U.L., Dattani, M., Keogh, J.M., et al. (2009). A deletion of the HBII-85 class of small nucleolar RNAs (snoRNAs) is associated with hyperphagia, obesity and hypogonadism. Hum. Mol. Genet. *18*, 3257–3265. https://doi.org/10.1093/hmg/ddp263.
10. Duker, A.L., Ballif, B.C., Bawle, E.V., Person, R.E., Mahadevan, S., Alliman, S., Thompson, R., Traylor, R., Bejjani, B.A., Shaffer, L.G., et al. (2010). Paternally inherited microdeletion at 15q11.2 confirms a significant role for the *SNORD116* C/D box snoRNA cluster in Prader–Willi syndrome. Eur. J. Hum. Genet. *18*, 1196–1201. https://doi.org/10.1038/ejhg.2010.102.
11. Cavaillé, J., Buiting, K., Kiefmann, M., Lalande, M., Brannan, C.I., Horsthemke, B., Bachellerie, J.P., Brosius, J., and Hüttenhofer, A. (2000). Identification of brain-specific and imprinted small nucleolar RNA genes exhibiting an unusual genomic organization. Proc. Natl. Acad. Sci. USA *97*, 14311–14316. https://doi.org/10.1073/pnas.250426397.
12. Hüttenhofer, A., Kiefmann, M., Meier-Ewert, S., O'Brien, J., Lehrach, H., Bachellerie, J.P., and Brosius, J. (2001). RNomics: an experimental approach that identifies 201 candidates for novel, small, non-messenger RNAs in mouse. EMBO J. *20*, 2943–2953. https://doi.org/10.1093/emboj/20.11.2943.
13. Watkins, N.J., and Bohnsack, M.T. (2012). The box C/D and H/ACA snoRNPs: key players in the modification, processing and the dynamic folding of ribosomal RNA. Wiley Interdiscip. Rev. RNA *3*, 397–414. https://doi.org/10.1002/wrna.117.
14. Bachellerie, J.P., Cavaillé, J., and Hüttenhofer, A. (2002). The expanding snoRNA world. Biochimie *84*, 775–790.
15. Liu, J., and Maxwell, E.S. (1990). Mouse U14 snRNA is encoded in an intron of the mouse cognate hsc70 heat shock gene. Nucleic Acids Res. *18*, 6565–6571. https://doi.org/10.1093/nar/18.22.6565.
16. Pellizzoni, L., Crosio, C., Campioni, N., Loreni, F., and Pierandrei-Amaldi, P. (1994). Different forms of U15 snoRNA are encoded in the introns of the ribosomal protein S1 gene of *Xenopus laevis*. Nucleic Acids Res. *22*, 4607–4613. https://doi.org/10.1093/nar/22.22.4607.
17. Kufel, J., and Grzechnik, P. (2019). Small Nucleolar RNAs Tell a Different Tale. Trends Genet. *35*, 104–117. https://doi.org/10.1016/j.tig.2018.11.005.
18. Tycowski, K.T., Shu, M.-D., and Steitz, J.A. (1996). A mammalian gene with introns instead of exons generating stable RNA products. Nature *379*, 464–466. https://doi.org/10.1038/379464a0.
19. Kiss, T., and Filipowicz, W. (1995). Exonucleolytic processing of small nucleolar RNAs from pre-mRNA introns. Genes Dev. *9*, 1411–1424. https://doi.org/10.1101/gad.9.11.1411.
20. Petfalski, E., Dandekar, T., Henry, Y., and Tollervey, D. (1998). Processing of the Precursors to Small Nucleolar RNAs and rRNAs Requires Common Components. Mol. Cell Biol. *18*, 1181–1189.
21. Bizarro, J., Charron, C., Boulon, S., Westman, B., Pradet-Balade, B., Vandermoere, F., Chagot, M.-E., Hallais, M., Ahmad, Y., Leonhardt, H., et al. (2014). Proteomic and 3D structure analyses highlight the C/D box snoRNP assembly mechanism and its control. J. Cell Biol. *207*, 463–480. https://doi.org/10.1083/jcb.201404160.
22. Cahill, N.M., Friend, K., Speckmann, W., Li, Z.-H., Terns, R.M., Terns, M.P., and Steitz, J.A. (2002). Site-specific cross-linking analyses reveal an asymmetric protein distribution for a box C/D snoRNP. EMBO J. *21*, 3816–3828. https://doi.org/10.1093/emboj/cdf376.
23. Good, D.J., and Kocher, M.A. (2017). Phylogenetic Analysis of the *SNORD116* Locus. Genes *8*, 358. https://doi.org/10.3390/genes8120358.
24. Runte, M., Hüttenhofer, A., Gross, S., Kiefmann, M., Horsthemke, B., and Buiting, K. (2001). The IC-SNURF-SNRPN transcript serves as a host for multiple small nucleolar RNA species and as an antisense RNA for UBE3A. Hum. Mol. Genet. *10*, 2687–2700. https://doi.org/10.1093/hmg/10.23.2687.
25. Ding, F., Li, H.H., Zhang, S., Solomon, N.M., Camper, S.A., Cohen, P., and Francke, U. (2008). SnoRNA Snord116 (Pwcr1/MBII-85) deletion causes growth deficiency and hyperphagia in mice. PLoS One *3*, e1709. https://doi.org/10.1371/journal.pone.0001709.
26. Skryabin, B.V., Gubar, L.V., Seeger, B., Pfeiffer, J., Handel, S., Robeck, T., Karpova, E., Rozhdestvensky, T.S., and Brosius, J. (2007). Deletion of the MBII-85 snoRNA Gene Cluster in Mice Results in Postnatal Growth Retardation. PLoS Genet. *3*, e235. https://doi.org/10.1371/journal.pgen.0030235.
27. Powell, W.T., Coulson, R.L., Crary, F.K., Wong, S.S., Ach, R.A., Tsang, P., Alice Yamada, N., Yasui, D.H., and Lasalle, J.M. (2013). A Prader-Willi locus lncRNA cloud modulates diurnal genes and energy expenditure. Hum. Mol. Genet. *22*, 4318–4328. https://doi.org/10.1093/hmg/ddt281.
28. Coulson, R.L., Yasui, D.H., Dunaway, K.W., Laufer, B.I., Vogel Ciernia, A., Zhu, Y., Mordaunt, C.E., Totah, T.S., and LaSalle, J.M. (2018). Snord116-dependent diurnal rhythm of DNA methylation in mouse cortex. Nat. Commun. *9*, 1616. https://doi.org/10.1038/s41467-018-03676-0.
29. Marbach, F., Elgizouli, M., Rech, M., Beygo, J., Erger, F., Velmans, C., Stumpel, C.T.R.M., Stegmann, A.P.A., Beck-Wödl, S., Gillessen-Kaesbach, G., et al. (2020). The adult phenotype of Schaaf-Yang syndrome. Orphanet J. Rare Dis. *15*, 294. https://doi.org/10.1186/s13023-020-01557-8.
30. Schaaf, C.P., Gonzalez-Garay, M.L., Xia, F., Potocki, L., Gripp, K.W., Zhang, B., Peters, B.A., McElwain, M.A., Drmanac, R., Beaudet, A.L., et al. (2013). Truncating mutations of MAGEL2 cause Prader-Willi phenotypes and autism. Nat. Genet. *45*, 1405–1408. https://doi.org/10.1038/ng.2776.
31. Fountain, M.D., Aten, E., Cho, M.T., Juusola, J., Walkiewicz, M.A., Ray, J.W., Xia, F., Yang, Y., Graham, B.H., Bacino, C.A., et al. (2017). The phenotypic spectrum of Schaaf-Yang syndrome: 18 new affected individuals from 14 families. Genet. Med. *19*, 45–52. https://doi.org/10.1038/gim.2016.53.

32. McCarthy, J., Lupo, P.J., Kovar, E., Rech, M., Bostwick, B., Scott, D., Kraft, K., Roscioli, T., Charrow, J., Schrier Vergano, S.A., et al. (2018). Schaaf-Yang syndrome overview: Report of 78 individuals. Am. J. Med. Genet. A *176*, 2564–2574. https://doi.org/10.1002/ajmg.a.40650.
33. Schaaf, C.P., and Marbach, F. (2021). In Schaaf-Yang Syndrome, M.P. Adam, J. Feldman, and G.M. Mirzaa, et al., eds. (University of Washington, Seattle), pp. 1993–2024, GeneReviews® [Internet]. https://www.ncbi.nlm.nih.gov/books/NBK567492/.
34. Patak, J., Gilfert, J., Byler, M., Neerukonda, V., Thiffault, I., Cross, L., Amudhavalli, S., Pacio-Miguez, M., Palomares-Bralo, M., Garcia-Minaur, S., et al. (2019). MAGEL2-related disorders: A study and case series. Clin. Genet. *96*, 493–505. https://doi.org/10.1111/cge.13620.
35. Mejlachowicz, D., Nolent, F., Maluenda, J., Ranjatoelina-Randrianaivo, H., Giuliano, F., Gut, I., Sternberg, D., Laquerrière, A., and Melki, J. (2015). Truncating Mutations of MAGEL2, a Gene within the Prader-Willi Locus, Are Responsible for Severe Arthrogryposis. Am. J. Hum. Genet. *97*, 616–620. https://doi.org/10.1016/j.ajhg.2015.08.010.
36. Tacer, K.F., and Potts, P.R. (2017). Cellular and disease functions of the Prader-Willi Syndrome gene MAGEL2. Biochem. J. *474*, 2177–2190. https://doi.org/10.1042/BCJ20160616.
37. Hao, Y.-H., Doyle, J.M., Ramanathan, S., Gomez, T.S., Jia, D., Xu, M., Chen, Z.J., Billadeau, D.D., Rosen, M.K., and Potts, P.R. (2013). Regulation of WASH-dependent actin polymerization and protein trafficking by ubiquitination. Cell *152*, 1051–1064. https://doi.org/10.1016/j.cell.2013.01.051.
38. Doyle, J.M., Gao, J., Wang, J., Yang, M., and Potts, P.R. (2010). MAGE-RING protein complexes comprise a family of E3 ubiquitin ligases. Mol. Cell *39*, 963–974. https://doi.org/10.1016/j.molcel.2010.08.029.
39. Lee, A.K., and Potts, P.R. (2017). A Comprehensive Guide to the MAGE Family of Ubiquitin Ligases. J. Mol. Biol. *429*, 1114–1142. https://doi.org/10.1016/j.jmb.2017.03.005.
40. Sanderson, M.R., Fahlman, R.P., and Wevrick, R. (2021). The N-terminal domain of the Schaaf-Yang syndrome protein MAGEL2 likely has a role in RNA metabolism. J. Biol. Chem. *297*, 100959. https://doi.org/10.1016/j.jbc.2021.100959.
41. Bischof, J.M., Stewart, C.L., and Wevrick, R. (2007). Inactivation of the mouse Magel2 gene results in growth abnormalities similar to Prader-Willi syndrome. Hum. Mol. Genet. *16*, 2713–2719. https://doi.org/10.1093/hmg/ddm225.
42. Kozlov, S.V., Bogenpohl, J.W., Howell, M.P., Wevrick, R., Panda, S., Hogenesch, J.B., Muglia, L.J., van Gelder, R.N., Herzog, E.D., and Stewart, C.L. (2007). The imprinted gene Magel2 regulates normal circadian output. Nat. Genet. *39*, 1266–1272. https://doi.org/10.1038/ng2114.
43. Reznik, D.L., Yang, M.V., Albelda de la Haza, P., Jain, A., Spanjaard, M., Theiss, S., Schaaf, C.P., Malovannaya, A., Strong, T.V., Veeraragavan, S., et al. (2023). Magel2 truncation alters select behavioral and physiological outcomes in a rat model of Schaaf-Yang syndrome. Dis. Model. Mech. *16*, dmm049829. https://doi.org/10.1242/dmm.049829.
44. Branon, T.C., Bosch, J.A., Sanchez, A.D., Udeshi, N.D., Svinkina, T., Carr, S.A., Feldman, J.L., Perrimon, N., and Ting, A.Y. (2018). Efficient proximity labeling in living cells and organisms with TurboID. Nat. Biotechnol. *36*, 880–887. https://doi.org/10.1038/nbt.4201.
45. Crutcher, E., Pal, R., Naini, F., Zhang, P., Laugsch, M., Kim, J., Bajic, A., and Schaaf, C.P. (2019). mTOR and autophagy pathways are dysregulated in murine and human models of Schaaf-Yang syndrome. Sci. Rep. *9*, 15935. https://doi.org/10.1038/s41598-019-52287-2.
46. Reinhardt, P., Glatza, M., Hemmer, K., Tsytsyura, Y., Thiel, C.S., Höing, S., Moritz, S., Parga, J.A., Wagner, L., Bruder, J.M., et al. (2013). Derivation and Expansion Using Only Small Molecules of Human Neural Progenitors for Neurodegenerative Disease Modeling. PLoS One *8*, e59252. https://doi.org/10.1371/journal.pone.0059252.
47. Schindelin, J., Arganda-Carreras, I., Frise, E., Kaynig, V., Longair, M., Pietzsch, T., Preibisch, S., Rueden, C., Saalfeld, S., Schmid, B., et al. (2012). Fiji: an open-source platform for biological-image analysis. Nat. Methods *9*, 676–682. https://doi.org/10.1038/nmeth.2019.
48. Hutter, K., Lohmüller, M., Jukic, A., Eichin, F., Avci, S., Labi, V., Szabo, T.G., Hoser, S.M., Hüttenhofer, A., Villunger, A., et al. (2020). SAFB2 Enables the Processing of Suboptimal Stem-Loop Structures in Clustered Primary miRNA Transcripts. Mol. Cell *78*, 876–889.e6. https://doi.org/10.1016/j.molcel.2020.05.011.
49. Hoernes, T.P., Clementi, N., Faserl, K., Glasner, H., Breuker, K., Lindner, H., Hüttenhofer, A., and Erlacher, M.D. (2016). Nucleotide modifications within bacterial messenger RNAs regulate their translation and are able to rewire the genetic code. Nucleic Acids Res. *44*, 852–862. https://doi.org/10.1093/nar/gkv1182.
50. Untergasser, A., Ruijter, J.M., Benes, V., and van den Hoff, M.J.B. (2021). Web-based LinRegPCR: application for the visualization and analysis of (RT)-qPCR amplification and melting data. BMC Bioinf. *22*, 398. https://doi.org/10.1186/s12859-021-04306-1.
51. Ruijter, J.M., Ruiz Villalba, A., Hellemans, J., Untergasser, A., and van den Hoff, M.J.B. (2015). Removal of between-run variation in a multi-plate qPCR experiment. Biomol. Detect. Quantif. *5*, 10–14. https://doi.org/10.1016/j.bdq.2015.07.001.
52. Xie, F., Wang, J., and Zhang, B. (2023). RefFinder: a web-based tool for comprehensively analyzing and identifying reference genes. Funct. Integr. Genomics *23*, 125. https://doi.org/10.1007/s10142-023-01055-7.
53. Xie, F., Xiao, P., Chen, D., Xu, L., and Zhang, B. (2012). miRDeepFinder: a miRNA analysis tool for deep sequencing of plant small RNAs. Plant Mol. Biol. https://doi.org/10.1007/s11103-012-9885-2.
54. Ruijter, J.M., Barnewall, R.J., Marsh, I.B., Szentirmay, A.N., Quinn, J.C., van Houdt, R., Gunst, Q.D., and van den Hoff, M.J.B. (2021). Efficiency Correction Is Required for Accurate Quantitative PCR Analysis and Reporting. Clin. Chem. *67*, 829–842. https://doi.org/10.1093/clinchem/hvab052.
55. Wickham, H., Averick, M., Bryan, J., Chang, W., McGowan, L., François, R., Grolemund, G., Hayes, A., Henry, L., Hester, J., et al. (2019). Welcome to the tidyverse. J. Open Source Softw. *4*, 1686. https://doi.org/10.21105/joss.01686.
56. Zhang, X., Smits, A.H., van Tilburg, G.B., Ovaa, H., Huber, W., and Vermeulen, M. (2018). Proteome-wide identification of ubiquitin interactions using UbIA-MS. Nat. Protoc. *13*, 530–550. https://doi.org/10.1038/nprot.2017.147.
57. Mellacheruvu, D., Wright, Z., Couzens, A.L., Lambert, J.-P., St-Denis, N.A., Li, T., Miteva, Y.V., Hauri, S., Sardiu, M.E., Low, T.Y., et al. (2013). The CRAPome: a Contaminant Repository for Affinity Purification Mass Spectrometry Data. Nat. Methods *10*, 730–736. https://doi.org/10.1038/nmeth.2557.

58. Heberle, H., Meirelles, G.V., da Silva, F.R., Telles, G.P., and Minghim, R. (2015). InteractiVenn: a web-based tool for the analysis of sets through Venn diagrams. BMC Bioinf. *16*, 169. https://doi.org/10.1186/s12859-015-0611-3.
59. Kuleshov, M.V., Jones, M.R., Rouillard, A.D., Fernandez, N.F., Duan, Q., Wang, Z., Koplev, S., Jenkins, S.L., Jagodnik, K.M., Lachmann, A., et al. (2016). Enrichr: a comprehensive gene set enrichment analysis web server 2016 update. Nucleic Acids Res. *44*, W90–W97. https://doi.org/10.1093/nar/gkw377.
60. Chen, E.Y., Tan, C.M., Kou, Y., Duan, Q., Wang, Z., Meirelles, G.V., Clark, N.R., and Ma'ayan, A. (2013). Enrichr: interactive and collaborative HTML5 gene list enrichment analysis tool. BMC Bioinf. *14*, 128. https://doi.org/10.1186/1471-2105-14-128.
61. Xie, Z., Bailey, A., Kuleshov, M.V., Clarke, D.J.B., Evangelista, J.E., Jenkins, S.L., Lachmann, A., Wojciechowicz, M.L., Kropiwnicki, E., Jagodnik, K.M., et al. (2021). Gene Set Knowledge Discovery with Enrichr. Curr. Protoc. *1*, e90. https://doi.org/10.1002/cpz1.90.
62. Cavaillé, J. (2017). Box C/D Small Nucleolar RNA Genes and the Prader-Willi Syndrome: A Complex Interplay*8* (Wiley Interdiscip. Rev. RNA), pp. e1417. https://doi.org/10.1002/wrna.1417.
63. Kosugi, S., Hasebe, M., Tomita, M., and Yanagawa, H. (2009). Systematic identification of cell cycle-dependent yeast nucleocytoplasmic shuttling proteins by prediction of composite motifs. Proc. Natl. Acad. Sci. USA *106*, 10171–10176. https://doi.org/10.1073/pnas.0900604106.
64. Nguyen Ba, A.N., Pogoutse, A., Provart, N., and Moses, A.M. (2009). NLStradamus: a simple Hidden Markov Model for nuclear localization signal prediction. BMC Bioinf. *10*, 202. https://doi.org/10.1186/1471-2105-10-202.
65. Xu, D., Marquis, K., Pei, J., Fu, S.-C., Cağatay, T., Grishin, N.V., and Chook, Y.M. (2015). LocNES: a computational tool for locating classical NESs in CRM1 cargo proteins. Bioinforma. Oxf. Engl. *31*, 1357–1365. https://doi.org/10.1093/bioinformatics/btu826.
66. Hao, Y.-H., Fountain, M.D., Fon Tacer, K., Xia, F., Bi, W., Kang, S.-H.L., Patel, A., Rosenfeld, J.A., Le Caignec, C., Isidor, B., et al. (2015). USP7 Acts as a Molecular Rheostat to Promote WASH-Dependent Endosomal Protein Recycling and Is Mutated in a Human Neurodevelopmental Disorder. Mol. Cell *59*, 956–969. https://doi.org/10.1016/j.molcel.2015.07.033.
67. Jia, D., Gomez, T.S., Billadeau, D.D., and Rosen, M.K. (2012). Multiple repeat elements within the FAM21 tail link the WASH actin regulatory complex to the retromer. Mol. Biol. Cell *23*, 2352–2361. https://doi.org/10.1091/mbc.E11-12-1059.
68. Ryder, P.V., Vistein, R., Gokhale, A., Seaman, M.N., Puthenveedu, M.A., and Faundez, V. (2013). The WASH complex, an endosomal Arp2/3 activator, interacts with the Hermansky-Pudlak syndrome complex BLOC-1 and its cargo phosphatidylinositol-4-kinase type IIα. Mol. Biol. Cell *24*, 2269–2284. https://doi.org/10.1091/mbc.E13-02-0088.
69. Gomez, T.S., and Billadeau, D.D. (2009). A FAM21-containing WASH complex regulates retromer-dependent sorting. Dev. Cell *17*, 699–711. https://doi.org/10.1016/j.devcel.2009.09.009.
70. Kudo, N., Matsumori, N., Taoka, H., Fujiwara, D., Schreiner, E.P., Wolff, B., Yoshida, M., and Horinouchi, S. (1999). Leptomycin B inactivates CRM1/exportin 1 by covalent modification at a cysteine residue in the central conserved region. Proc. Natl. Acad. Sci. USA *96*, 9112–9117. https://doi.org/10.1073/pnas.96.16.9112.
71. Sun, Q., Carrasco, Y.P., Hu, Y., Guo, X., Mirzaei, H., Macmillan, J., and Chook, Y.M. (2013). Nuclear export inhibition through covalent conjugation and hydrolysis of Leptomycin B by CRM1. Proc. Natl. Acad. Sci. USA *110*, 1303–1308. https://doi.org/10.1073/pnas.1217203110.
72. Szklarczyk, D., Kirsch, R., Koutrouli, M., Nastou, K., Mehryary, F., Hachilif, R., Gable, A.L., Fang, T., Doncheva, N.T., Pyysalo, S., et al. (2023). The STRING database in 2023: protein-protein association networks and functional enrichment analyses for any sequenced genome of interest. Nucleic Acids Res. *51*, D638–D646. https://doi.org/10.1093/nar/gkac1000.
73. Kim, D.I., Birendra, K.C., Zhu, W., Motamedchaboki, K., Doye, V., and Roux, K.J. (2014). Probing nuclear pore complex architecture with proximity-dependent biotinylation. Proc. Natl. Acad. Sci. USA *111*, E2453–E2461. https://doi.org/10.1073/pnas.1406459111.
74. Olguin, S.L., Patel, P., Buchanan, C.N., Dell'Orco, M., Gardiner, A.S., Cole, R., Vaughn, L.S., Sundararajan, A., Mudge, J., Allan, A.M., et al. (2022). KHSRP loss increases neuronal growth and synaptic transmission and alters memory consolidation through RNA stabilization. Commun. Biol. *5*, 672. https://doi.org/10.1038/s42003-022-03594-4.
75. Baldini, L., Charpentier, B., and Labialle, S. (2021). Emerging Data on the Diversity of Molecular Mechanisms Involving C/D snoRNAs. Noncoding. RNA *7*, 30. https://doi.org/10.3390/ncrna7020030.
76. Ronchetti, D., Todoerti, K., Tuana, G., Agnelli, L., Mosca, L., Lionetti, M., Fabris, S., Colapietro, P., Miozzo, M., Ferrarini, M., et al. (2012). The expression pattern of small nucleolar and small Cajal body-specific RNAs characterizes distinct molecular subtypes of multiple myeloma. Blood Cancer J. *2*, e96. https://doi.org/10.1038/bcj.2012.41.
77. Kurosaki, T., Popp, M.W., and Maquat, L.E. (2019). Quality and quantity control of gene expression by nonsense-mediated mRNA decay. Nat. Rev. Mol. Cell Biol. *20*, 406–420. https://doi.org/10.1038/s41580-019-0126-2.
78. Carias, K.V., Zoeteman, M., Seewald, A., Sanderson, M.R., Bischof, J.M., and Wevrick, R. (2020). A MAGEL2-deubiquitinase complex modulates the ubiquitination of circadian rhythm protein CRY1. PLoS One *15*, e0230874. https://doi.org/10.1371/journal.pone.0230874.
79. Chen, H., Victor, A.K., Klein, J., Tacer, K.F., Tai, D.J., de Esch, C., Nuttle, A., Temirov, J., Burnett, L.C., Rosenbaum, M., et al. (2020). Loss of MAGEL2 in Prader-Willi syndrome leads to decreased secretory granule and neuropeptide production. JCI Insight *5*, e138576. https://doi.org/10.1172/jci.insight.138576.
80. Castilla-Vallmanya, L., Centeno-Pla, M., Serrano, M., Franco-Valls, H., Martínez-Cabrera, R., Prat-Planas, A., Rojano, E., Ranea, J.A.G., Seoane, P., Oliva, C., et al. (2023). Advancing in Schaaf-Yang syndrome pathophysiology: from bedside to subcellular analyses of truncated MAGEL2. J. Med. Genet. *60*, 406–415. https://doi.org/10.1136/jmg-2022-108690.
81. Centeno-Pla, M., Alcaide-Consuegra, E., Gibson, S., Prat-Planas, A., Gutiérrez-Ávila, J.D., Grinberg, D., Urreizti, R., Rabionet, R., and Balcells, S. (2024). Subcellular localisation of truncated MAGEL2 proteins: insight into the molecular pathology of Schaaf-Yang syndrome. J. Med. Genet. https://doi.org/10.1136/jmg-2024-109898.

82. Buiting, K., Di Donato, N., Beygo, J., Bens, S., von der Hagen, M., Hackmann, K., and Horsthemke, B. (2014). Clinical phenotypes of MAGEL2 mutations and deletions. Orphanet J. Rare Dis. *9*, 40. https://doi.org/10.1186/1750-1172-9-40.
83. Kanber, D., Giltay, J., Wieczorek, D., Zogel, C., Hochstenbach, R., Caliebe, A., Kuechler, A., Horsthemke, B., and Buiting, K. (2009). A paternal deletion of MKRN3, MAGEL2 and NDN does not result in Prader-Willi syndrome. Eur. J. Hum. Genet. *17*, 582–590. https://doi.org/10.1038/ejhg.2008.232.
84. Matarazzo, V., and Muscatelli, F. (2013). Natural breaking of the maternal silence at the mouse and human imprinted Prader-Willi locus: A whisper with functional consequences. Rare Dis. *1*, e27228. https://doi.org/10.4161/rdis.27228.
85. Hong, D.-H., Pawlyk, B.S., Adamian, M., and Li, T. (2004). Dominant, Gain-of-Function Mutant Produced by Truncation of RPGR. Invest. Ophthalmol. Vis. Sci. *45*, 36–41. https://doi.org/10.1167/iovs.03-0787.
86. Yang, H., Kurtenbach, S., Guo, Y., Lohse, I., Durante, M.A., Li, J., Li, Z., Al-Ali, H., Li, L., Chen, Z., et al. (2018). Gain of function of ASXL1 truncating protein in the pathogenesis of myeloid malignancies. Blood *131*, 328–341. https://doi.org/10.1182/blood-2017-06-789669.
87. Arai, M., Sugase, K., Dyson, H.J., and Wright, P.E. (2015). Conformational propensities of intrinsically disordered proteins influence the mechanism of binding and folding. Proc. Natl. Acad. Sci. *112*, 9614–9619. https://doi.org/10.1073/pnas.1512799112.
88. Bondos, S.E., Dunker, A.K., and Uversky, V.N. (2021). On the roles of intrinsically disordered proteins and regions in cell communication and signaling. Cell Commun. Signal. *19*, 88. https://doi.org/10.1186/s12964-021-00774-3.
89. Wimmer, M.C., Brennenstuhl, H., Hirsch, S., Dötsch, L., Unser, S., Caro, P., and Schaaf, C.P. (2024). Hao-Fountain syndrome: 32 novel patients reveal new insights into the clinical spectrum. Clin. Genet. *105*, 499–509. https://doi.org/10.1111/cge.14480.
90. Binda, O., Juillard, F., Ducassou, J.N., Kleijwegt, C., Paris, G., Didillon, A., Baklouti, F., Corpet, A., Couté, Y., Côté, J., et al. (2023). SMA-linked SMN mutants prevent phase separation properties and SMN interactions with FMRP family members. Life Sci. Alliance *6*, e202201429. https://doi.org/10.26508/lsa.202201429.
91. Piazzon, N., Rage, F., Schlotter, F., Moine, H., Branlant, C., and Massenet, S. (2008). In Vitro and in Cellulo Evidences for Association of the Survival of Motor Neuron Complex with the Fragile X Mental Retardation Protein. J. Biol. Chem. *283*, 5598–5610. https://doi.org/10.1074/jbc.M707304200.
92. Tadesse, H., Deschênes-Furry, J., Boisvenue, S., and Côté, J. (2008). KH-type splicing regulatory protein interacts with survival motor neuron protein and is misregulated in spinal muscular atrophy. Hum. Mol. Genet. *17*, 506–524. https://doi.org/10.1093/hmg/ddm327.
93. Chaytow, H., Huang, Y.-T., Gillingwater, T.H., and Faller, K.M.E. (2018). The role of survival motor neuron protein (SMN) in protein homeostasis. Cell. Mol. Life Sci. *75*, 3877–3894. https://doi.org/10.1007/s00018-018-2849-1.
94. Groen, E.J.N., Fumoto, K., Blokhuis, A.M., Engelen-Lee, J., Zhou, Y., van den Heuvel, D.M.A., Koppers, M., van Diggelen, F., van Heest, J., Demmers, J.A.A., et al. (2013). ALS-associated mutations in FUS disrupt the axonal distribution and function of SMN. Hum. Mol. Genet. *22*, 3690–3704. https://doi.org/10.1093/hmg/ddt222.
95. Fischer, U., Liu, Q., and Dreyfuss, G. (1997). The SMN–SIP1 Complex Has an Essential Role in Spliceosomal snRNP Biogenesis. Cell *90*, 1023–1029. https://doi.org/10.1016/S0092-8674(00)80368-2.
96. Lefebvre, S., Bürglen, L., Reboullet, S., Clermont, O., Burlet, P., Viollet, L., Benichou, B., Cruaud, C., Millasseau, P., Zeviani, M., et al. (1995). Identification and characterization of a spinal muscular atrophy-determining gene. Cell *80*, 155–165. https://doi.org/10.1016/0092-8674(95)90460-3.
97. Lorson, C.L., Hahnen, E., Androphy, E.J., and Wirth, B. (1999). A single nucleotide in the SMN gene regulates splicing and is responsible for spinal muscular atrophy. Proc. Natl. Acad. Sci. USA *96*, 6307–6311. https://doi.org/10.1073/pnas.96.11.6307.
98. Eggert, C., Chari, A., Laggerbauer, B., and Fischer, U. (2006). Spinal muscular atrophy: the RNP connection. Trends Mol. Med. *12*, 113–121. https://doi.org/10.1016/j.molmed.2006.01.005.
99. Bostrom, C., Yau, S.Y., Majaess, N., Vetrici, M., Gil-Mohapel, J., and Christie, B.R. (2016). Hippocampal dysfunction and cognitive impairment in Fragile-X Syndrome. Neurosci. Biobehav. Rev. *68*, 563–574. https://doi.org/10.1016/j.neubiorev.2016.06.033.
100. Richter, J.D., and Zhao, X. (2021). The Molecular Biology of FMRP: New Insights into Fragile X Syndrome. Nat. Rev. Neurosci. *22*, 209–222. https://doi.org/10.1038/s41583-021-00432-0.
101. Laggerbauer, B., Ostareck, D., Keidel, E.-M., Ostareck-Lederer, A., and Fischer, U. (2001). Evidence that fragile X mental retardation protein is a negative regulator of translation. Hum. Mol. Genet. *10*, 329–338. https://doi.org/10.1093/hmg/10.4.329.
102. Hale, C.R., Sawicka, K., Mora, K., Fak, J.J., Kang, J.J., Cutrim, P., Cialowicz, K., Carroll, T.S., and Darnell, R.B. (2021). FMRP regulates mRNAs encoding distinct functions in the cell body and dendrites of CA1 pyramidal neurons. Elife *10*, e71892. https://doi.org/10.7554/eLife.71892.
103. Antar, L.N., Li, C., Zhang, H., Carroll, R.C., and Bassell, G.J. (2006). Local functions for FMRP in axon growth cone motility and activity-dependent regulation of filopodia and spine synapses. Mol. Cell. Neurosci. *32*, 37–48. https://doi.org/10.1016/j.mcn.2006.02.001.
104. Bechara, E.G., Didiot, M.C., Melko, M., Davidovic, L., Bensaid, M., Martin, P., Castets, M., Pognonec, P., Khandjian, E.W., Moine, H., et al. (2009). A novel function for fragile X mental retardation protein in translational activation. PLoS Biol. *7*, e16. https://doi.org/10.1371/journal.pbio.1000016.
105. Chen, C.Y., Gherzi, R., Ong, S.E., Chan, E.L., Raijmakers, R., Pruijn, G.J., Stoecklin, G., Moroni, C., Mann, M., and Karin, M. (2001). AU binding proteins recruit the exosome to degrade ARE-containing mRNAs. Cell *107*, 451–464. https://doi.org/10.1016/s0092-8674(01)00578-5.
106. Gherzi, R., Lee, K.-Y., Briata, P., Wegmüller, D., Moroni, C., Karin, M., and Chen, C.-Y. (2004). A KH domain RNA binding protein, KSRP, promotes ARE-directed mRNA turnover by recruiting the degradation machinery. Mol. Cell *14*, 571–583. https://doi.org/10.1016/j.molcel.2004.05.002.
107. Prior, T.W., Leach, M.E., and Finanger, E. (2000). In Spinal Muscular Atrophy, *1993–2024*, M.P. Adam, J. Feldman, and

G.M. Mirzaa, et al., eds. *(University of Washington, Seattle), GeneReviews® [Internet].* *https://www.ncbi.nlm.nih.gov/books/NBK1352/*.
108. Nowicki, S.T., Tassone, F., Ono, M.Y., Ferranti, J., Croquette, M.F., Goodlin-Jones, B., and Hagerman, R.J. (2007). The Prader-Willi Phenotype of Fragile X Syndrome. J. Dev. Behav. Pediatr. *28*, 133–138. https://doi.org/10.1097/01.DBP.0000267563.18952.c9.
109. Bozdagi, O., Sakurai, T., Dorr, N., Pilorge, M., Takahashi, N., and Buxbaum, J.D. (2012). Haploinsufficiency of Cyfip1 Produces Fragile X-Like Phenotypes in Mice. PLoS One *7*, e42422. https://doi.org/10.1371/journal.pone.0042422.
110. Chang, H.-C., Hung, W.-C., Chuang, Y.-J., and Jong, Y.-J. (2004). Degradation of survival motor neuron (SMN) protein is mediated via the ubiquitin/proteasome pathway. Neurochem. Int. *45*, 1107–1112. https://doi.org/10.1016/j.neuint.2004.04.005.
111. Nalavadi, V.C., Muddashetty, R.S., Gross, C., and Bassell, G.J. (2012). Dephosphorylation-Induced Ubiquitination and Degradation of FMRP in Dendrites: A Role in Immediate Early mGluR-Stimulated Translation. J. Neurosci. *32*, 2582–2587. https://doi.org/10.1523/JNEUROSCI.5057-11.2012.
112. Grohmann, K., Varon, R., Stolz, P., Schuelke, M., Janetzki, C., Bertini, E., Bushby, K., Muntoni, F., Ouvrier, R., Van Maldergem, L., et al. (2003). Infantile spinal muscular atrophy with respiratory distress type 1 (SMARD1). Ann. Neurol. *54*, 719–724. https://doi.org/10.1002/ana.10755.
113. Grohmann, K., Schuelke, M., Diers, A., Hoffmann, K., Lucke, B., Adams, C., Bertini, E., Leonhardt-Horti, H., Muntoni, F., Ouvrier, R., et al. (2001). Mutations in the gene encoding immunoglobulin mu-binding protein 2 cause spinal muscular atrophy with respiratory distress type 1. Nat. Genet. *29*, 75–77. https://doi.org/10.1038/ng703.
114. Maystadt, I., Zarhrate, M., Landrieu, P., Boespflug-Tanguy, O., Sukno, S., Collignon, P., Melki, J., Verellen-Dumoulin, C., Munnich, A., and Viollet, L. (2004). Allelic heterogeneity of SMARD1 at the IGHMBP2 locus. Hum. Mutat. *23*, 525–526. https://doi.org/10.1002/humu.9241.
115. Pitt, M., Houlden, H., Jacobs, J., Mok, Q., Harding, B., Reilly, M., and Surtees, R. (2003). Severe infantile neuropathy with diaphragmatic weakness and its relationship to SMARD1. Brain *126*, 2682–2692. https://doi.org/10.1093/brain/awg278.
116. Vadla, G.P., Ricardez Hernandez, S.M., Mao, J., Garro-Kacher, M.O., Lorson, Z.C., Rice, R.P., Hansen, S.A., Lorson, C.L., Singh, K., and Lorson, M.A. (2023). ABT1 modifies SMARD1 pathology via interactions with IGHMBP2 and stimulation of ATPase and helicase activity. JCI Insight *8*, e164608. https://doi.org/10.1172/jci.insight.164608.
117. Guenther, U.-P., Handoko, L., Laggerbauer, B., Jablonka, S., Chari, A., Alzheimer, M., Ohmer, J., Plöttner, O., Gehring, N., Sickmann, A., et al. (2009). IGHMBP2 is a ribosome-associated helicase inactive in the neuromuscular disorder distal SMA type 1 (DSMA1). Hum. Mol. Genet. *18*, 1288–1300. https://doi.org/10.1093/hmg/ddp028.
118. Saladini, M., Nizzardo, M., Govoni, A., Taiana, M., Bresolin, N., Comi, G.P., and Corti, S. (2020). Spinal muscular atrophy with respiratory distress type 1: Clinical phenotypes, molecular pathogenesis and therapeutic insights. J. Cell Mol. Med. *24*, 1169–1178. https://doi.org/10.1111/jcmm.14874.
119. Butler, M.G., Manzardo, A.M., Heinemann, J., Loker, C., and Loker, J. (2017). Causes of death in Prader-Willi syndrome: Prader-Willi Syndrome Association (USA) 40-year mortality survey. Genet. Med. *19*, 635–642. https://doi.org/10.1038/gim.2016.178.
120. Pacoricona Alfaro, D.L., Lemoine, P., Ehlinger, V., Molinas, C., Diene, G., Valette, M., Pinto, G., Coupaye, M., Poitou-Bernert, C., Thuilleaux, D., et al. (2019). Causes of death in Prader-Willi syndrome: lessons from 11 years' experience of a national reference center. Orphanet J. Rare Dis. *14*, 238. https://doi.org/10.1186/s13023-019-1214-2.
121. Suresh Babu, S., Joladarashi, D., Jeyabal, P., Thandavarayan, R.A., and Krishnamurthy, P. (2015). RNA stabilizing proteins as molecular targets in cardiovascular pathologies. Trends Cardiovasc. Med. *25*, 676–683. https://doi.org/10.1016/j.tcm.2015.02.006.
122. Chen, X., Li, A., Sun, B.-F., Yang, Y., Han, Y.-N., Yuan, X., Chen, R.-X., Wei, W.-S., Liu, Y., Gao, C.-C., et al. (2019). 5-methylcytosine promotes pathogenesis of bladder cancer through stabilizing mRNAs. Nat. Cell Biol. *21*, 978–990. https://doi.org/10.1038/s41556-019-0361-y.
123. Johnson, Z., Xu, X., Lin, Y., and Xie, H. (2023). Dynamics of RNA m5C modification during brain development. Genomics *115*, 110604. https://doi.org/10.1016/j.ygeno.2023.110604.
124. Soeno, Y., Taya, Y., Stasyk, T., Huber, L.A., Aoba, T., and Hüttenhofer, A. (2010). Identification of novel ribonucleo-protein complexes from the brain-specific snoRNA MBII-52. RNA *16*, 1293–1300. https://doi.org/10.1261/rna.2109710.
125. Hausser, J., and Zavolan, M. (2014). Identification and consequences of miRNA–target interactions — beyond repression of gene expression. Nat. Rev. Genet. *15*, 599–612. https://doi.org/10.1038/nrg3765.
126. Crenshaw, M.M., Graw, S.L., Slavov, D., Boyle, T.A., Piqué, D.G., Taylor, M., and Baker, P. (2023). An Atypical 15q11.2 Microdeletion Not Involving *SNORD116* Resulting in Prader-Willi Syndrome. Case Rep. Genet. *2023*, 4225092. https://doi.org/10.1155/2023/4225092.
127. Grootjen, L.N., Juriaans, A.F., Kerkhof, G.F., and Hokken-Koelega, A.C.S. (2022). Atypical 15q11.2-q13 Deletions and the Prader-Willi Phenotype. J. Clin. Med. *11*, 4636. https://doi.org/10.3390/jcm11154636.

The American Journal of Human Genetics, Volume *111*

Supplemental information

Truncated variants of MAGEL2 are involved in the etiologies of the Schaaf-Yang and Prader-Willi syndromes

David Heimdörfer, Alexander Vorleuter, Alexander Eschlböck, Angeliki Spathopoulou, Marta Suarez-Cubero, Hesso Farhan, Veronika Reiterer, Melanie Spanjaard, Christian P. Schaaf, Lukas A. Huber, Leopold Kremser, Bettina Sarg, Frank Edenhofer, Stephan Geley, Mariana E.G. de Araujo, and Alexander Huettenhofer

Supplemental Material

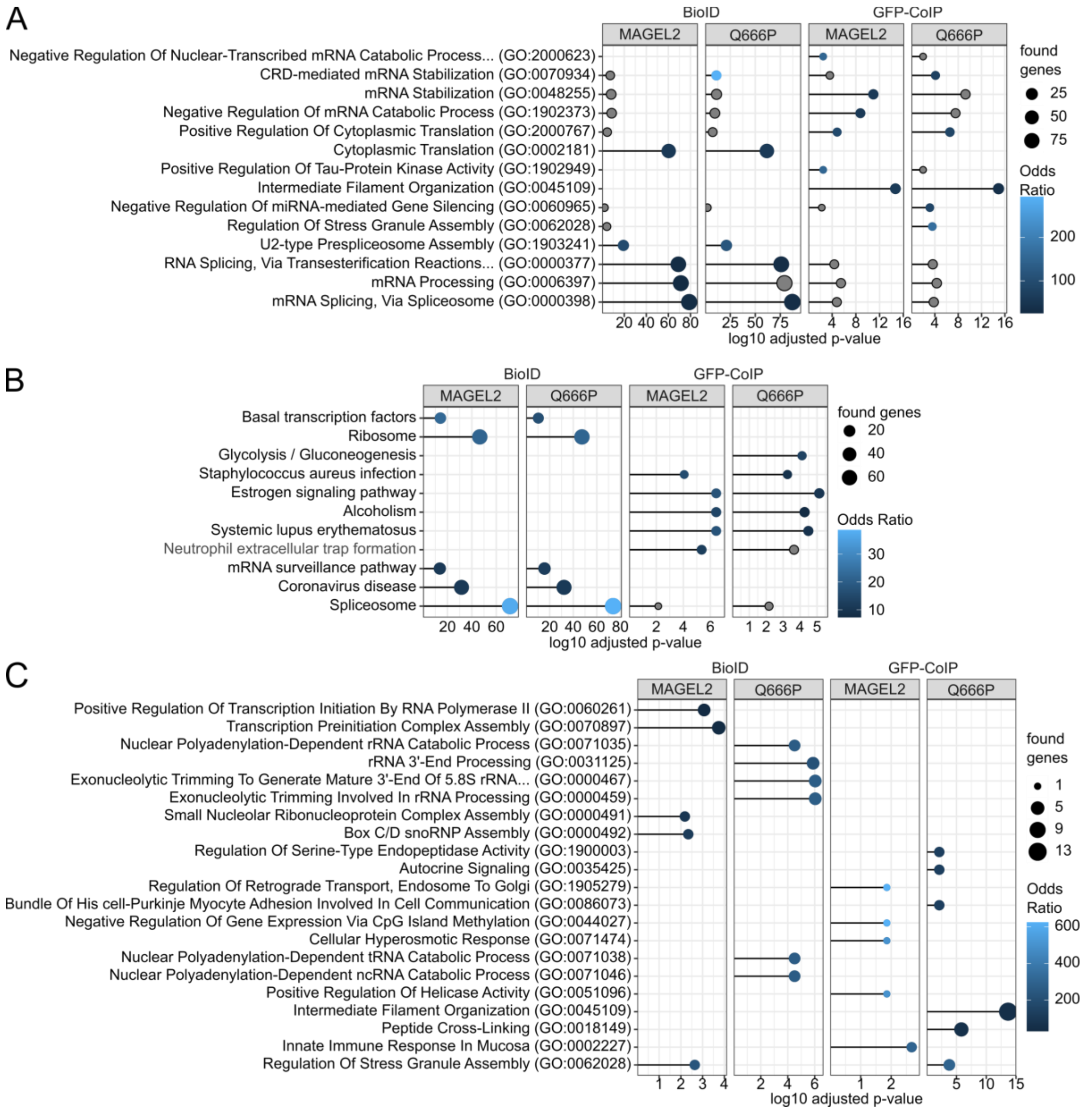

Figure S1: GO enrichment analysis reveals putative interactors of MAGEL2 and MAGEL2 p.(Gln666Profs*47) (referred to as Q666P in the figure) to be involved in transcription, mRNA stabilization, snoRNA metabolism, and metabolism. A and B: GO enrichment analysis for proteins identified in 2 out of 3 replicates showing Biological Processes (A) or KEGG pathways (B). C: GO enrichment analysis of proteins, which were 2-fold enriched in either BioID or GFP-CoIP showing Biological Processes. A-C: GO terms with an adjusted p-value <0.05 were ordered by Combined Score [1]. Top 5 hits are indicated in shades of blue. Light grey circles indicate that the respective GO term is among top hits in one of the other conditions.

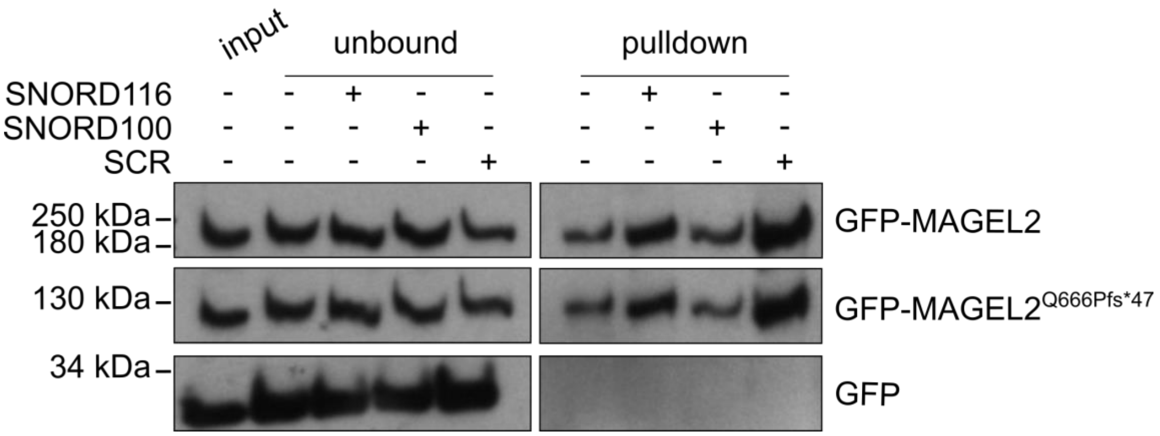

Figure S2: Western blot analysis following RNA-CoIP of HEK293T IP lysates with transiently overexpressed GFP, GFP-MAGEL2 and GFP- MAGEL2 p.(Gln666Profs*47).

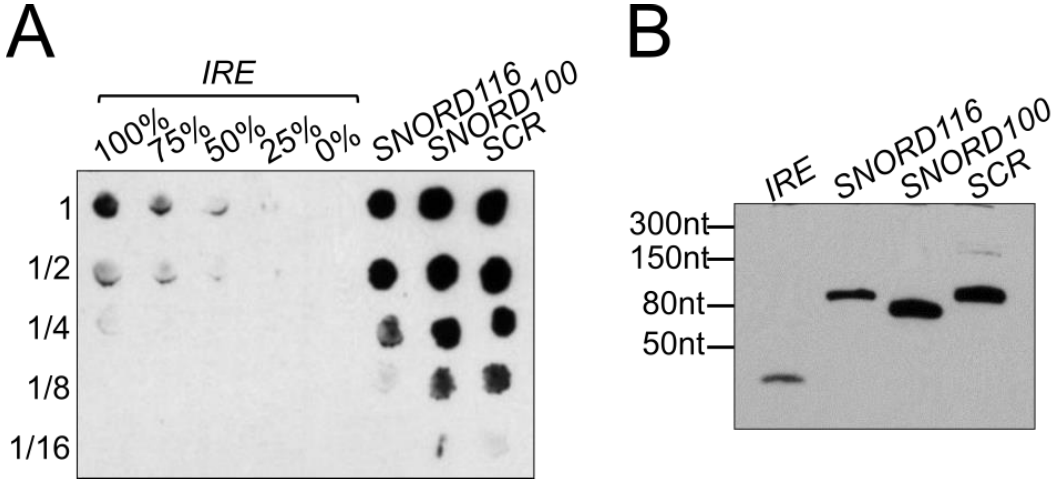

Figure S3: Effieciency of biotinylation of *SNORD116*, *SNORD100* and the *SCR* control. A and B: 3'-biotinylation of RNAs was analyzed by dot blot analysis employing a dilution series (A) and on an 8% polyacrylamide gel (7 M urea) (B). As control served a biotinylated IRE RNA from the company (Thermo Fisher Scientific).

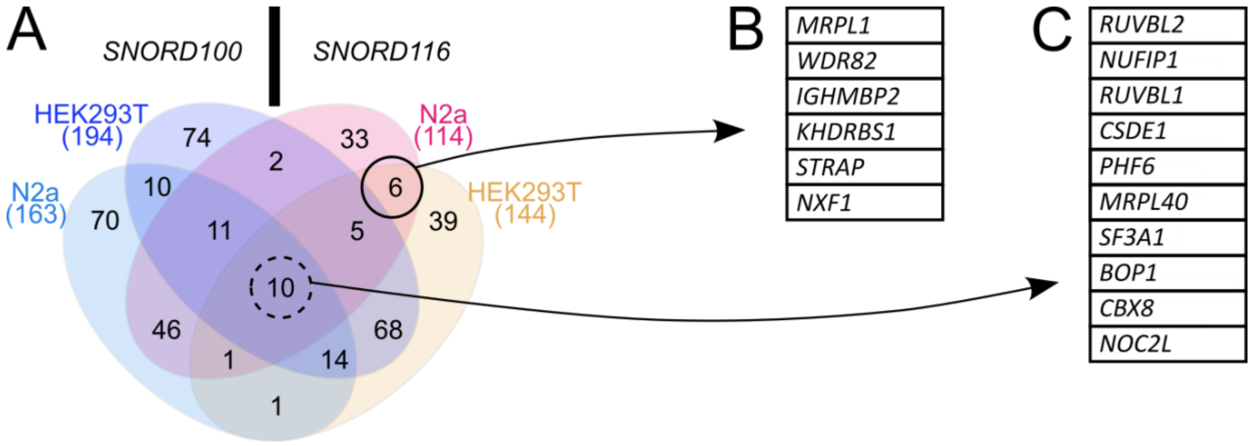

Figure S4: A: Venn diagram of interactomes identified by RNA-CoIP with 3’-biotinylated RNAs in HEK293T and N2A IP lysates. Only proteins identified in at least 2 of 3 replicates were analyzed. Putative unspecific interactors were removed employing a scrambled RNA control (see Fig 4B) for per cell type. B: Specific proteins (gene names depicted) bound to *SNORD116* in HEK293T and N2A. C: Mutual proteins (gene names depicted) of *SNORD116* and *SNORD100* in both examined cell types.

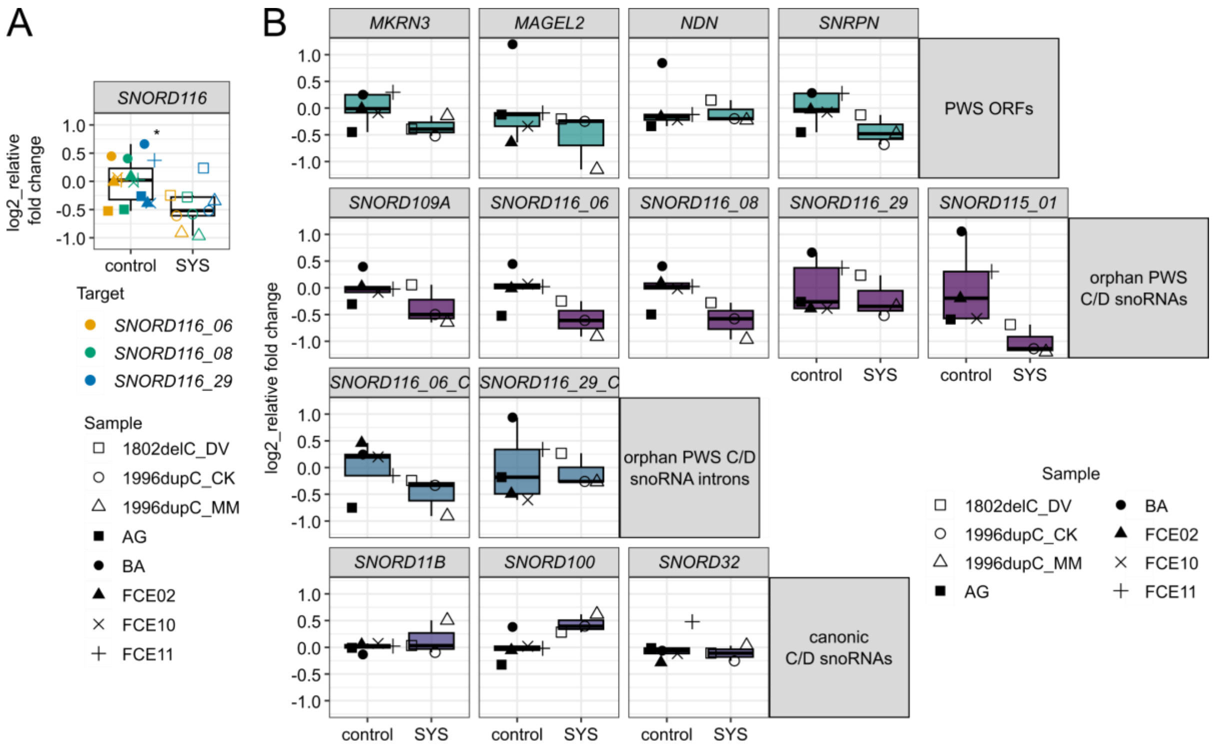

Figure S5: Expression analysis of genes from the PWS region by qPCR analysis. A: Pooled RT-qPCRs for investigated *SNORD116* copies 06, 08, and 29 in smNPCs, rstatix[2] was used for the calculation of Wilcoxon tests B: .RT-qPCR of individual transcripts in smNPCs, which were used for analysis in ***Error! Reference source not found.***A.

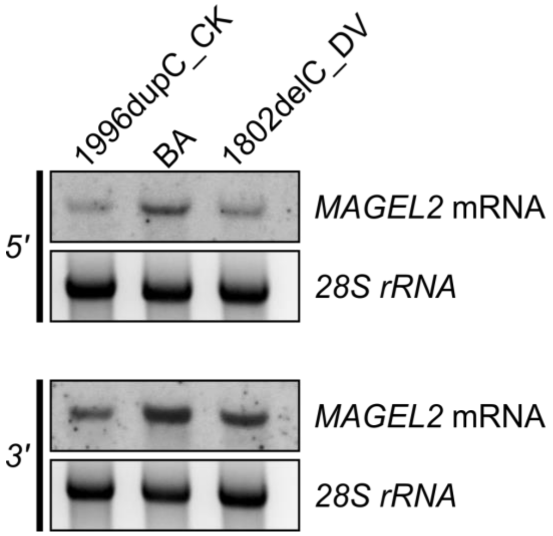

Figure S6: Northern blot analysis of wt and mutant MAGEL2 mRNA transcripts from healthy and SYS smNPCs. Probes for expression analysis of wt (BA) or mutant (1996dupC_CKMAGEL2, 1802delC_DV) mRNAs are indicated as 5’ or 3’, respectively; as a loading control expression of 28S rRNA is shown.

antibody	company	product-no.	clonality [clone]	host	dilution
**anti-EEA1**	Abcam	ab109110	mono [EPR4245]	rabbit	750 (HEK 293T)<br>500 (N2a)
**anti-RAB11A**	Cell signaling	#2413	poly	rabbit	50
**anti-FMRP**	Cell Signaling	#4317	poly	rabbit	50
**anti-SMN**	Proteintech	#11708	poly	rabbit	400
**anti-RAB11A**	Thermo Fischer	#715300	poly	rabbit	50
**CoraLite488-conjugated anti-Rabbit**	Proteintech	SA00013-2	poly	goat	250

Table S1: Antibodies used in IF-analyses. All antibodies employed in this study are depicted above, as well as the host they were derived from and the dilution used in the study.

Target	Sequence [5' to 3']	forward [5' to 3']	reverse [5' to 3']
SNORD116_3/9	**GGATCCTAATACGACTCACTATAGGATCGATGATGAGTCCCCCATAAAAACATTCCTTGGAAAAGCTGAACAAAATGAGTGAGAACTCATACCGTCGTTCTCATCGGAACTGAGGTCC**	GGATCCTAATACGACTCAC	GGACCTCAGTTCCGATGA
SNORD100	**GGATCCTAATACGACTCACTATAGCTGTACATGATGACAACTGGCTCCCTCTACTGAACTGCCATGAGGAAACTGCCATGTCACCCTTCTGATTACAGC**	GGATCCTAATACGACTCAC	GCTGTAATCAGAAGGGTGA
SCR	**GCTAATACGACTCACTATAGAAGATCAGTTGGGTGCACGAGTGGGTTACATCGAACTGGATCTCAACAGCGGTAAGATCCTTGAGAGTTTTCGCCCCGAAGAACGTTTTCCAAT**	GCTAATACGACTCACTATAGAAGATCAGTTGGGTGCAC	ATTGGAAAACGTTCTTCGGG

Table S2: Sequences of snoRNAs and SCR control RNA sequences (bold). Sequences were employed for the generation of T7 transcripts and their respective primers are indicated. The T7 promoter sequence is underlined.

antibody	company	product-no.	clonality [clone]	host	dilution
**anti-FMRP**	Abcam	ab264380	poly	rabbit	5,000
**anti-SMN**	Abcam	ab108531	mono [EPR4429]	rabbit	1,000
**anti-NUFIP1**	Cell signaling	#37783	poly	rabbit	1,000
**anti-USP7**	Abcam	ab264422	mono [BLR072G]	rabbit	1,000
**anti-GFP**	Roche	11814460001	mono [7.1 and 13.1]	mouse	1,000
**anti-mouse**	Dako	P0447	poly	goat	3,000
**anti-rabbit**	Thermo Fischer	31463	poly	goat	3,000 - 10,000

Table S3: Antibodies used in western blot analyses. Indicated are all antibodies as well as their clonality, the host they were derived from and the dilution which was employed in experiments.

name	*Sequence [5' to 3']*	*forward [5' to 3']*	*reverse [5' to 3']*
M2_5' (356 nt)	CTGGGTCTCTCTGAAAGCCCAGGG AGCTCTCTGCCCGTAGTTGTGTCTG AGGTCGCAAGTGTCTCTCCGGGAT CCAGTGCCACCCAGGATAATTCCAA GGTGGAGGCACAGCCCTTGTCTCC CTTGGATGAGAGGGCAAATGCGTT GGTGCAGTTCCTCTTAGTCAAGGAC CAAGCCAAGGTGCCTGTCCAGCGC TCGGAGATGGTGAAAGTCATCCTCC GAGAGTATAAAGATGAGTGCTTAGA TATCATCAACCGTGCCAACAATAAG CTGGAGTGTGCCTTTGGTTATCAAT TGAAAGAAATTGATACCAAAAACCA CGCCTATATTATCATCAACAAGCTG GGCTACCATACAGGGAATTTGGTGG CATCCTATTTAGACAGGCCC	GTACACCGATGG CCCAG	AACTTGAGACTGG ATTTGCAG
M2_3' (390 nt)	CTGGGTCTCTCTGAAAGCCCAGGG AGCTCTCTGCCCGTAGTTGTGTCTG AGGTCGCAAGTGTCTCTCCGGGAT CCAGTGCCACCCAGGATAATTCCAA GGTGGAGGCACAGCCCTTGTCTCC CTTGGATGAGAGGGCAAATGCGTT GGTGCAGTTCCTCTTAGTCAAGGAC CAAGCCAAGGTGCCTGTCCAGCGC TCGGAGATGGTGAAAGTCATCCTCC GAGAGTATAAAGATGAGTGCTTAGA TATCATCAACCGTGCCAACAATAAG CTGGAGTGTGCCTTTGGTTATCAAT TGAAAGAAATTGATACCAAAAACCA CGCCTATATTATCATCAACAAGCTG GGCTACCATACAGGGAATTTGGTGG CATCCTATTTAGACAGGCCC	CTGGGTCTCTCTG AAAGCCCAG	GGGCCTGTCTAA ATAGGATGCC

Table S4: DIG probe sequences and oligonucleotides employed for probe generation. Forward and reverse primers for PCR amplification are indicated.

**Target**	**Forward [5‘ to 3‘]**	**Reverse [5‘ to 3‘]**
**GAPDH**	CCATGGGGAAGGTGAAGGTC	AGTTAAAAGCAGCCCTGGTGA
**EID2**	GGCATCGCTCTGTCCAGTTA	GCTTGGACATCTCAGACCGT
**TBP**	GCAAGGGTTTCTGGTTTGCC	CAAGCCCTGAGCGTAAGGTG
**HPRT**	AGGCGAACCTCTCGGCTTTC	CTGGTTCATCATCACTAATCACGAC
**TUBB**	CAACCAGATCGGGGCCAAGTT	GAGGCACGTACTTGTGAGAAGA
**MAGEL2**	CAAGTTTGGCCTTCTGATGGTG	CGGACATCCAACCCTAACTTGA
**MKRN3**	CATTGAGTTTGTTCCAGGGCAG	TCTGCTTCCTCTCAGTCTCTGA
**SNORD115_1**	GAGAACCTTATATTATCCTGAAGAGA GGTG	GGCCTCAGCGTAATCCTATTGA
**SNORD116_29**	GGACCTCAGCTCACAGAAGTG	GGATCGATGATGACTTAAAAAAATGG AAAC
**SNORD116_08**	GGATCGATGATGAGTCCTCCAA	GGACCTCAGTTCCGATGAGAAC
**SNORD116_06**	GGATCGATGATGAGTCCTCCAA	GGACCTCAGTTCCGATGAGAAT

**SNORD116_06_C**	GTGGAAGTTGTCCCTCACTAGTA	CAAAATGAGTGAAAACTCATACCGTC
**SNORD116_29_C**	CAAACTGAACGCCCTCCAC	AAATGAGTGACCAAGACACTTCTG
**SNRPN**	ACTGTTGGCAAGAGTAGCAAGA	GGCCATCTTGCAGGATACATCT
**NDN**	TACTCCACGAGGGTGTTTTCTG	AGTTTGGAAGAAGTCACCAGCA
**SNORD109A**	GGATCGATGATGAGAATAATTGTC	GGACCTCAGATTGACATCTG
**SNORD11B**	CTGATGGCAATGATGATTTTTACAC	GATGGCATCAGATGAGGTAGT
**SNORD32**	CAGTGATGAGCAACATTCACCA	GAGCGGTGCATGGGGTTGAT
**SNORD100**	GCTGTACATGATGACAACTGGC	GTGACATGGCAGTTTCCTCATG

Table S5: Primers used in qPCR analysis. For quantification of gene expression by qPCR forward and reverse primers for respective genes are indicated.

**MAGEL2**			**MAGEL2 p.(Gln666Profs*47)**		
Position	Sequence	Score	Position	Sequence	Score
4-33	LSKNLGDSSPPAEAPKPPV YSRPTVLMRAP	4.3	4-33	LSKNLGDSSPPAEAPKPPV YSRPTVLMRAP	4.3
277-307	PMAKPPGPGVLMIHPPGAR APMTQPPASGAP	2.3	277-307	PMAKPPGPGVLMIHPPGAR APMTQPPASGAP	2.3
292-322	PGARAPMTQPPASGAPMA QPAAPPAQPMAPP	2.3	292-322	PGARAPMTQPPASGAPMA QPAAPPAQPMAPP	2.3
494-527	RQAPPPIRPAPQVLATQPPL WQALPPPPPLRQAP	3.1	494-527	RQAPPPIRPAPQVLATQPPL WQALPPPPPLRQAP	3.1
587-617	WQAPKGQPPVPHEIPTSM EFQEVQQTQALAW	2.5	587-617	WQAPKGQPPVPHEIPTSM EFQEVQQTQALAW	2.5
617-648	WQAQKAPTHIWQPLPAQE AQRQAPPLVQLEQP	2.8	617-648	WQAQKAPTHIWQPLPAQE AQRQAPPLVQLEQP	2.8
654-684	PSQKAVQIQLPPQQAQAS GPQAEVPTLPLQP	2.5	654-683	PSQKAVQIQLPPPAGPGIG SASGGAHTAAP	2.1
709-739	GSAKSLMTPSGECRASSID RRGSSKERRTSS	3.1			
728-760	RRGSSKERRTSSKERRAPS KDRMIFAATFCAPK	3.0			
735-764	RRTSSKERRAPSKDRMIFA ATFCAPKAVSA	2.5			
735-766	RRTSSKERRAPSKDRMIFA ATFCAPKAVSAAR	3.0			
735-770	RRTSSKERRAPSKDRMIFA ATFCAPKAVSAARAHLP	2.0			
742-774	RRAPSKDRMIFAATFCAPK	2.4			

	AVSAARAHLPAAWK				
802-832	NAFKGPSAASETPKSLPYA LQDPFACVEALP	2.7			
880-914	RRSGKATRKKKHLEAQEDS RGHTLAFHDWQGPRPW	2.9			
880-915	RRSGKATRKKKHLEAQEDS RGHTLAFHDWQGPRPWE	2.3			
881-915	RSGKATRKKKHLEAQEDS RGHTLAFHDWQGPRPWE	2.9			
887-916	RKKKHLEAQEDSRGHTLAF HDWQGPRPWEN	2.5			
952-979	SRILSGWEGPSASWALSA WEGPSTSRAL	2.4			
1161-1192	FVRQKYLEYRRIPYTEPAE YEFLWGPRAFLET	2.0			
1163-1197	RQKYLEYRRIPYTEPAEYE FLWGPRAFLETSKMLV	2.4			
883-893	GKATRKKKHLE	8.5			

Tab S6: Nuclear localization signal (NLS) motif prediction performed with the cNLS mapper online tool [3]**.** NLS are listed for MAGEL2 protein and the truncated MAGEL2 p.(Gln666Profs*47). Shown are amino acid position, NLS candidate sequence and cut-off score.

MAGEL2			MAGEL2 p.(Gln666Profs*47)		
Position	Sequence	Score	Position	Sequence	Score
65-79	WEAPQGQLPAPVVPM	0.024	65-79	WEAPQGQLPAPVVPM	0.022
235-249	PGVLMAQPLTPGVLM	0.011	235-249	PGVLMAQPLTPGVLM	0.124
236-250	GVLMAQPLTPGVLMV	0.068	236-250	GVLMAQPLTPGVLMV	0.255
592-606	GQPPVPHEIPTSMEF	0.042	592-606	GQPPVPHEIPTSMEF	0.031
668-682	AQASGPQAEVPTLPL	0.022			
694-708	QAQPGPPVAAANFPL	0.046			
842-856	MNASKASQAVPTFLM	0.017			
985-999	PGSSLPVVVSEVASV	0.074			
1016-1030	PLSPLDERANALVQF	0.109			
1018-1032	SPLDERANALVQFLL	0.144			
1019-1033	PLDERANALVQFLLV	0.186			
1103-1117	SYLDRPKFGLLMVVL	0.262			

1105-1119	LDRPKFGLLMVVLSL	0.204			
1106-1120	DRPKFGLLMVVLSLI	0.115			
1107-1121	RPKFGLLMVVLSLIF	0.473			
1108-1122	PKFGLLMVVLSLIFM	0.201			
1123-1137	KGNCVREDLIFNFLF	0.051			
1127-1141	VREDLIFNFLFKLGL	0.332			
1129-1143	EDLIFNFLFKLGLDV	0.238			
1146-1160	TNGLFGNTKKLITEV	0.007			
1148-1162	GLFGNTKKLITEVFV	0.005			
1183-1197	LWGPRAFLETSKMLV	0.015			
1184-1198	WGPRAFLETSKMLVL	0.186			
1186-1200	PRAFLETSKMLVLRF	0.084			

Table S7: Nuclear export signal (NES) motif prediction performed with the LocNES online sever tool [4]. NES are listed for MAGEL2 and the truncated MAGEL2 p.(Gln666Profs*47). Shown are amino acid position, NES candidate sequence and probability score.

Term	Genes
**Positive Regulation Of DNA-templated Transcription (GO:0045893)**	**HMGA1, SFPQ, TAF15, ILF3, HMGN1, YBX1**
**Regulation Of DNA-templated Transcription (GO:0006355)**	**FUS, ILF3, YBX1, HMGA1, SFPQ, TAF15**
**Regulation Of Nucleic Acid-Templated Transcription (GO:1903506)**	**FUS, HMGA1, SFPQ, YBX1**
**Regulation Of Transcription By RNA Polymerase II (GO:0006357)**	**FUS, YBX1, HMGA1, SFPQ**
Negative Regulation Of DNA-templated Transcription (GO:0045892)	HMGA1, SFPQ, ILF3
Negative Regulation Of Nucleic Acid-Templated Transcription (GO:1903507)	ILF3, HMGA1, SFPQ
Positive Regulation Of Nucleic Acid-Templated Transcription (GO:1903508)	ILF3, HMGA1, TAF15
Positive Regulation Of Transcription By RNA Polymerase II (GO:0045944)	HMGA1, SFPQ, YBX1
DNA-templated Transcription Initiation (GO:0006352)	ELOB
Negative Regulation Of Transcription By RNA Polymerase II (GO:0000122)	SFPQ
Positive Regulation Of DNA-templated Transcription, Elongation (GO:0032786)	HMGN1
Positive Regulation Of Transcription Regulatory Region DNA Binding (GO:2000679)	H1-0
Regulation Of DNA-templated Transcription Elongation (GO:0032784)	HMGN1
Regulation Of Transcription Regulatory Region DNA Binding (GO:2000677)	H1-0

Transcription By RNA Polymerase II (GO:0006366)	ELOB
Transcription Initiation At RNA Polymerase II Promoter (GO:0006367)	ELOB

Table S8: Mutual protein interaction partners of MAGEL2 and MAGEL2 p.(Gln666Profs*47) with implication in transcription.

Summarized term	GO terms included
Transcription	Transcription By RNA Polymerase II (GO:0006366)
	Transcription Initiation At RNA Polymerase II Promoter (GO:0006367)
	Transcription Preinitiation Complex Assembly (GO:0070897)
snoRNAs	Box C/D snoRNP Assembly (GO:0000492)
	Polyadenylation-Dependent snoRNA 3'-End Processing (GO:0071051)
	snoRNA Localization (GO:0048254)
mRNA stabilization	3'-UTR-mediated mRNA Stabilization (GO:0070935)
	CRD-mediated mRNA Stabilization (GO:0070934)
	mRNA Stabilization (GO:0048255)
	Regulation Of 3'-UTR-mediated mRNA Stabilization (GO:1905868)
Pyruvate metabolism and Gluconeogenesis	Gluconeogenesis (GO:0006094)
	Negative Regulation Of Gluconeogenesis (GO:0045721)
	Pyruvate Metabolic Process (GO:0006090)
	Positive Regulation Of Gluconeogenesis (GO:0045722)
	Regulation Of Gluconeogenesis (GO:0006111)

Table S9: GO terms significantly regulated in the two different approaches (BioID and GFP-CoIP).

Supplemental References

1. Chen, E.Y., Tan, C.M., Kou, Y., Duan, Q., Wang, Z., Meirelles, G.V., Clark, N.R., and Ma'ayan, A. (2013). Enrichr: interactive and collaborative HTML5 gene list enrichment analysis tool. BMC Bioinformatics *14*, 128. https://doi.org/10.1186/1471-2105-14-128.

2. Kassambara, A. (2023). rstatix: Pipe-Friendly Framework for Basic Statistical Tests.

3. Kosugi, S., Hasebe, M., Tomita, M., and Yanagawa, H. (2009). Systematic identification of cell cycle-dependent yeast nucleocytoplasmic shuttling proteins by prediction of composite motifs. Proc. Natl. Acad. Sci. U. S. A. *106*, 10171–10176. https://doi.org/10.1073/pnas.0900604106.

4. Xu, D., Marquis, K., Pei, J., Fu, S.-C., Cağatay, T., Grishin, N.V., and Chook, Y.M. (2015). LocNES: a computational tool for locating classical NESs in CRM1 cargo proteins. Bioinforma. Oxf. Engl. *31*, 1357–1365. https://doi.org/10.1093/bioinformatics/btu826.
